# Occupation and urinary phthalate metabolite concentrations in a national survey of adults in Canada

**DOI:** 10.1186/s12940-026-01297-5

**Published:** 2026-04-24

**Authors:** Patrick Hinton, Ryann E. Yeo, Joanne Kim, Daniel R.S. Middleton, Katherine Pullella, Victoria Arrandale, Nathan L. DeBono

**Affiliations:** 1 Dalla Lana School of Public Health, University of Toronto, Toronto, Canada; 2 Occupational Cancer Research Centre, Ontario Health, Toronto, Canada; 3 Environment and Lifestyle Epidemiology Branch, International Agency for Research on Cancer, WHO, Lyon, France; 4 Centre for Public Health, School of Medicine, Dentistry and Biomedical Sciences, Queen’s University Belfast, Belfast, UK; 5 Department of Nutritional Sciences, Temerty Faculty of Medicine, University of Toronto, Toronto, ON Canada

**Keywords:** Phthalates, Occupational exposure, Biomonitoring, Canadian Health Measures Survey

## Abstract

**Background:**

Phthalates are synthetic chemicals with widespread exposure linked to adverse health effects. Occupational determinants remain understudied despite the potential for substantially elevated workplace exposure levels. This study aimed to investigate the associations between occupation and urinary phthalate metabolite concentrations in a national sample of Canadian adults.

**Methods:**

We analyzed data from 4,259 adults, aged 20–79 years, from four cycles (2007–2019) of the cross-sectional Canadian Health Measures Survey, which quantified 11 urinary phthalate metabolites. We compared creatinine-corrected concentrations of individual metabolites and four summary measures of phthalates across occupation (10 broad- and 40 major-level groups) and industry (19 sectors). Multivariable linear regression models estimated geometric mean ratios (GMRs) and 95% confidence intervals (CIs). For four metabolites with low detection frequencies, multivariable logistic regression estimated odds ratios of detection. Models were adjusted for sociodemographic, lifestyle, and dietary confounders. Sensitivity analyses compared results using specific gravity-corrected concentrations.

**Results:**

Non-occupational factors, including female sex, Asian and Black ethnicity, and higher fruit and vegetable consumption, were associated with elevated phthalate concentrations. Workers in natural resources, agriculture, and related production had elevated concentrations of most metabolites, including mono(3-carboxypropyl) phthalate (MCPP) (GMR = 1.64, 95% CI: 1.15, 2.33) and monobenzyl phthalate (MBzP) (GMR = 1.41, 95% CI: 0.97, 2.03). Workers in trades, transport, and equipment operation also exhibited modestly increased exposures, particularly trades helpers and construction labourers for mono-n-butyl phthalate (MBP) (GMR = 1.41, 95% CI: 1.05, 1.89) and MCPP (GMR = 1.80, 95% CI: 1.22, 2.67). Elevated concentrations were also observed among workers in service support and other service occupations, with precise increases across several metabolites. Elevated concentrations in construction and industrial trades were largely driven by males, while females were the primary drivers of elevated di(2-ethylhexyl) phthalate (DEHP) metabolites in natural resources and agriculture, and of increases more broadly in service support occupations.

**Conclusion:**

This study identifies significantly elevated phthalate exposures among Canadian workers in agriculture, construction, and trades, as well as in service support roles. These findings highlight novel at-risk occupational groups beyond traditional manufacturing settings providing critical evidence for targeting exposure reduction and disease prevention strategies.

**Supplementary Information:**

The online version contains supplementary material available at 10.1186/s12940-026-01297-5.

## Background

Phthalates, also known as phthalate esters, are diesters of phthalic acid and represent a large class of synthetic aromatic chemicals with widespread application as plasticizers and stabilizers in industrial materials and consumer products [1]. Phthalates have been associated with a wide-range of adverse health outcomes [1]. High molecular weight phthalates (HMWPs), typically ≥ 250 g/mol with long carbon side chains, are used to increase the flexibility and durability of various materials, such as polyvinyl chloride (PVC) polymers, food packaging, and building supplies. Low molecular weight phthalates (LMWPs), those < 250 g/mol, are primarily used as solvents, adhesives, and fixatives in cosmetics, personal care products (e.g., fragrances, lotions), and pharmaceutical coatings [2, 3]. HMWPs and LMWPs also differ in their toxicological properties and, to some extent, their associated health endpoints [4, 5], for example, LMWPs have been associated with reproductive toxicity via anti-androgenic mechanisms [6, 7], whereas HMWPs, especially di(2-ethylhexyl) phthalate (DEHP), have been implicated in hormone-dependent cancers (e.g., breast, prostate cancer) [8]. In Canada, phthalate regulation focuses primarily on DEHP, which is restricted in children’s toys, vinyl-based products, medical devices, and cosmetics; however, restrictions on other phthalate esters remain comparatively limited in scope and application [9].

Since phthalates are not covalently bound to plastic polymer matrices, they can leech from their source products, resulting in ubiquitous exposure via multiple routes: ingestion, inhalation, dermal absorption, or intravenous [3, 4]. Once in the body, phthalates are rapidly metabolised into monoesters and excreted via urine, typically within two days [10]. In a nationally-representative human biomonitoring survey, select urinary phthalate metabolites were detected in 100% of sampled Canadians aged 6–79 years [11].

Among the studied determinants of exposure, dietary intake is an established influential source [12, 13]. Additional key determinants include the use of cosmetics and personal care products [14], socioeconomic factors [15–17], ethnicity [3, 4, 15, 16], smoking [10, 18, 19], and residential characteristics, such as the presence of PVC flooring and other phthalate-containing building materials [20]. In contrast, few human biomonitoring studies have systematically examined occupational risk factors for exposure [21].

Importantly, occupational phthalate exposure levels often far exceed background environmental levels. For example, among Taiwanese PVC manufacturing workers, the geometric mean (GM) urinary concentration (µg/g-creatinine) of mono-(2-ethyl-5-hydroxyhexyl) phthalate (MEHHP, a primary metabolite of DEHP) was more than four times higher than that observed in university graduate student controls [22]. Existing studies, however, have been limited to small samples from select occupational groups identified a priori as having high exposure, such as workers in plastics and PVC manufacturing, waste management, and cosmetic or beauty salons [21]. Data covering a broader range of occupations, including other potentially at-risk groups, remains scarce.

Using data from the Canadian Health Measures Survey (CHMS), this study aimed to: (1) quantify urinary concentrations of 11 phthalate metabolites and metabolite summary measures (sum of LMWPs [∑LMWP], HMWPs [∑HMWP], DEHP-devolving metabolites [∑DEHP], and total phthalates [∑Total phthalates]); (2) investigate associations between urinary phthalate concentrations with current occupation and industry of employment, while also examining relationships with non-occupational determinants of phthalate exposure, including dietary, sociodemographic, and lifestyle factors; and (3) evaluate sex-specific differences in occupational phthalate exposures.

## Methods

### Study population

The CHMS is a national and ongoing, stratified cross-sectional survey of Canadians aged 3–79 years; full design details are reported elsewhere [23–25]. The target population includes citizens and permanent residents, excluding residents of the territories (Cycles 3–6), certain remote regions, institutional settings, full-time military personnel, and individuals living on Indigenous reserves or settlements [23–25]. Data collection includes in-home interviews and subsequent visits to mobile examination centers (MECs). Household interviews capture demographic, lifestyle, socioeconomic, occupational, health, and environmental information [24]. At MECs, trained personnel collect biological specimens and anthropometric measurements, where selected environmental chemicals are quantified in serum and urine from an age-stratified subsample of each CHMS cycle [24]. Phthalate metabolites were measured in four cycles: Cycle 1 (2007–2009), Cycle 2 (2009–2011), Cycle 5 (2016–2017), and Cycle 6 (2018–2019).

For this study, the analytic sample was restricted to the *n* = 4,259 adults aged 20–79 years who had available measurements for at least one phthalate metabolite [11, 26–29]. Respondents were included if they were not pregnant at the time of sample collection. Additional exclusion criteria are listed in Supplemental Fig. 1.

### Urinary phthalate measurement and dilution adjustment

In each of the four cycles, spot urine samples, collected at participants’ examination times (morning or afternoon) and obtained immediately upon arrival to the MEC, were analyzed for monomethyl phthalate (MMP), monoethyl phthalate (MEP), mono-n-butyl phthalate (MnBP), monobenzyl phthalate (MBzP), mono-cyclohexyl phthalate (MCHP), mono(2-ethylhexyl) phthalate (MEHP), mono(2-ethyl-5-oxohexyl) phthalate (MEOHP), MEHHP, mono-octyl phthalate (MOP), mono(3-carboxypropyl) phthalate (MCPP), and mono-isononyl phthalate (MiNP) using standardized CHMS protocols [11, 24, 26–28, 30, 31]. To account for urinary dilution, creatinine concentrations were measured using the colorimetric Jaffe method [11, 26–28, 32], and urine specific gravity (SG) was measured from Cycle 2 onward [11, 27, 28].

For this study, metabolite concentrations were corrected for urinary creatinine (µg/g-creatinine), and grouped into summary metrics: ∑Total phthalates, ∑LMWPs (< 250 g/mol), ∑HMWPs (≥ 250 g/mol), and ∑DEHP-devolving metabolites (Supplemental Table 1). Summary groups were calculated as creatinine-corrected molar sums (nmol/g-creatinine). Summary metrics for other parent phthalates (e.g., di-n-butyl phthalate [DBP]) were not created because only one metabolite was measured, although the primary metabolite was available for most compounds. SG-corrected concentrations (µg/L for individual metabolites; nmol/L for summary groups) were computed using the Levine-Fahy equation [33, 34]. Creatinine standardization was used for primary analyses to maximize sample size across CHMS cycles and statistical precision for occupational subgroup comparisons, given that SG was not available in Cycle 1. SG-adjusted models were examined in sensitivity analyses for Cycles 2, 5, and 6. Despite mostly consistent analytical techniques, limits of detection (LODs) varied across cycles and metabolites (Supplemental Table 2). Concentrations below LODs were substituted with LOD/√2 in a metabolite- and cycle-specific manner [4, 35].

### Assessment and classification of occupation and industry

Occupational information was obtained during household interviews, including current employment status, employer type, and job title [23]. Information on duration of current employment was not collected in the CHMS cycles used for this analysis. For current employment at the time of interview, industry (employer type) and occupation (job title) codes were provided in standardized four-level hierarchical coding systems: North American Industry Classification System (NAICS) [36, 37] and the Canadian National Occupational Classification (NOC) [38, 39], respectively. These were harmonized to the NAICS 2017 and NOC 2016 revisions for comparability across cycles. To maximize statistical power, the current study focuses on the 10 broad- and 40 major-level occupational groups (1- and 2-digit NOC 2016, respectively), as well as the 19 sector-level industry groups (2-digit NAICS 2017); unemployed and retired participants (at the time of interview) were retained and classified as a separate group.

### Covariate data

Dietary data were obtained via non-quantitative food-frequency questionnaires (FFQs) during household interviews, capturing usual intake frequencies for various food and beverage items (Supplemental Table 3). The CHMS FFQ has been partially validated against 24-hour recalls from the Canadian Community Health Survey, with similar estimates for vegetables, fruits, and milk products but greater uncertainty for meat and alternatives and grain products [40, 41]. Consumption frequency of each dietary category was assessed annually. Total household income was self-reported in Cycles 1–2, derived from tax records in Cycles 5–6 [42–45], and categorized into cycle-specific quartiles. Ethnicity was modeled as White, Black, Asian, or other. Self-reported smoking status distinguished non/former from current smokers [10, 46]. Body mass index (BMI) was calculated from measured height and weight and categorized using standard cut-points [47]. Urban-rural residence was defined using postal code linkages to census metropolitan areas; household water source was classified as municipal or other (e.g., private drilled wells/cisterns), and drinking water type was classified as tap, bottled, or other. Oral medication use, given potential phthalate content [48], was grouped as 0, 1–2, 3–5, or > 5 unique products in the past month. Alcohol consumption was categorized as non-drinker, 1–4, 5–8, or > 8 drinks per week. Physical activity was converted to weekly minutes (assuming 30 min/session) and categorized into cycle-specific tertiles.

### Statistical analysis

Multivariable linear regression models were fitted to estimate adjusted associations between current occupation and industry of employment, respectively, with log-transformed and creatinine-corrected phthalate metabolite concentrations. Models estimated least-squares GMs and geometric mean ratios (GMRs), along with their 95% confidence intervals (CIs), for the high detection frequency (DFs ≥ 70%) metabolites (*n* = 7) and to ∑LMWP, ∑HMWP, ∑DEHP, and ∑Total. We calculated model-predicted GMs, rather than arithmetic means, to account for the log-normal distribution of phthalate concentrations and to maintain consistency with previous studies. Estimates were derived by exponentiating marginal means estimated using the *emmeans* package in R [49]. Occupation and industry of employment were represented as binary indicators comparing workers in the occupation of interest to all other participants, including those unemployed or retired. For metabolites with DFs < 70% (*n* = 4), we used multivariable logistic regression to estimate odds ratios (ORs) and 95% CIs for detection, adjusting for the same covariates as in the linear models. For these models, metabolites were categorized by detection status using the highest cycle- and metabolite-specific LOD as a common threshold across cycles [50].

Covariates were selected a priori using directed acyclic graphs (DAGs) (Supplemental Fig. 2) and prior evidence of established (strong, consistent association) and potential phthalate determinants [3, 4, 10, 12, 13, 15–19, 48, 50–54]. Established determinants (e.g., diet, ethnicity, smoking) were automatically selected for multivariable model adjustment assuming DAG-compliance. Crude bivariate associations between potential determinants and metabolite concentrations were assessed using two approaches: (1) qualitative examination of differences in summary group GMs across determinant categories, and (2) non-parametric ANOVA tests (Mann-Whitney for binary, Kruskal-Wallis for multi-category variables). To address multiple comparisons, we applied the Benjamini-Hochberg procedure [55] with a false discovery rate (FDR) of 0.25. Potential determinants were selected for multivariable modeling if they demonstrated substantial heterogeneity across summary group GMs and yielded FDR-adjusted significance (assuming uncorrected α = 0.05) in ≥ 4 of 7 metabolites with DF ≥ 70%.

Splines (3–5 knots) and functional forms of age were compared using the Akaike information criterion (AIC). The final adjusted model included age (continuous), sex, CHMS Cycle, ethnicity, household income, smoking status, six dietary groups (meat and alternatives, dairy, rice and grains, fruits and vegetables, seafood, bottled and canned beverages), drinking water type, and physical activity. All dietary groups were retained due to the established role of diet as a major exposure source [12, 13]. Although BMI displayed heterogeneity in bivariate analyses, it was excluded from the final model since most phthalate metabolites have limited bioaccumulation potential [46]. Physical activity was treated for as a confounder rather than a mediator; although occupation may influence activity levels, the cross-sectional design precludes temporal ordering, and activity primarily affects phthalate metabolite concentrations by elimination rather than exposure [10, 21, 56]. Model assumptions and multicollinearity were assessed using standard diagnostics.

Sensitivity analyses compared least-squares GMRs for summary phthalate groups using SG instead of creatinine to adjust for urinary dilution, to both assess the robustness of associations observed in the main analyses and evaluate the impact of the dilution method. CHMS survey weights were not incorporated in our analyses given that our objective was to estimate associations between occupational characteristics and urinary phthalate concentrations rather than to generate nationally-representative estimates. Analyses therefore prioritized internal validity and precision of effect estimates when pooling multiple cycles and examining occupational subgroups. All analyses were conducted using R Statistical Software, version 4.1.2 [57]. Due to Statistics Canada Research Data Centre disclosure requirements, sample sizes for some small occupational groups and descriptive categories are not presented.

## Results

The study population (*n* = 4,259) consisted mostly of participants who were White (78%), completed post-secondary education (68%), resided in urban areas (87%), and used municipal water at home (68%) (Table 1). Differences in the proportion of males and females were observed according to several sociodemographic and lifestyle factors: women were more often in the lowest income quartile, non-drinkers, and higher consumers of fruits and vegetables, whereas men were more likely to be current smokers, heavy alcohol consumers, and higher meat consumers. The distribution of broad- and major-level NOC occupations and sector-level NAICS industries are shown in Supplemental Tables 4 and 5, respectively. The largest broad-level occupational groups among CHMS participants were in *sales and service* (15%) and *business*,* finance*,* and administration* (13%). Men were more often employed in: *trades*,* transport*,* and equipment operation* (95% male), *manufacturing and utilities* (76% male), and *natural and applied sciences* (76% male); while women were more often employed in *health* (78% female), *education*,* law*,* and social*,* community and government services* (66% female), and *business*,* finance*,* and administration* (66% female). Over 25% of the analytic sample (*n* = 1,082) was not employed at the time of CHMS participation, with the majority being retired.

**Table 1:** Descriptive characteristics of CHMS participants with urinary phthalate measurements, 2007–2019

Characteristic	Males, % (*n* = 2,135)	Females, % (*n* = 2,124)	Overall, % (*n* = 4,259)
CHMS Cycle (Years)			
Cycle 1 (2007–2009)	27.9	27.8	27.9
Cycle 2 (2009–2011)	23.5	23.3	23.4
Cycle 5 (2016–2017)	25.2	25.0	25.1
Cycle 6 (2018–2019)	23.5	23.9	23.7
CHMS Year			
2007	10.5	11.3	10.9
2008	14.9	14.2	14.6
2009	6.4	6.3	6.3
2010	10.5	12.1	11.3
2011	9.0	7.2	8.1
2016	11.5	10.6	11.0
2017	13.7	14.4	14.0
2018	13.1	12.5	12.8
2019	10.4	11.4	10.9
Age at Clinic Visit (Years)			
20–39	41.5	40.6	41.1
40–59	36.0	35.6	35.8
60–79	22.5	23.7	23.1
Ethnicity			
White	77.7	79.0	78.4
Black	2.2	2.7	2.4
Asian	11.8	9.9	10.8
Other^a^	8.3	8.2	8.3
Education Level Attained			
Below Secondary Graduation	9.3	8.0	8.7
Secondary Graduation	17.5	17.6	17.5
Some Post-Secondary	4.8	5.0	4.9
Post-Secondary Graduation	67.5	68.6	68.1
Total Household Income^b^ (Quartiles)			
Q1 (lowest income)	19.8	26.6	23.2
Q2	24.3	25.5	24.9
Q3	27.6	23.0	25.3
Q4 (highest income)	28.3	24.8	26.6
Body Mass Index (kg/m²)			
< 18.5	1.2	1.9	1.6
≥ 18.5 to < 25.0	29.4	42.3	35.8
≥ 25.0 to < 30.0	44.1	28.0	36.0
≥ 30.0	24.7	27.4	26.1
Smoking Status^c^			
Current Smoker	23.3	17.9	20.6
Former Smoker	29.9	27.0	28.5
Non-Smoker	46.8	55.1	51.0
Alcohol Consumption (drinks/week)			
Non-Drinker	33.5	46.8	40.1
1 ≤ 4	24.1	28.7	26.4
5 ≤ 8	15.2	13.6	14.4
≥ 8	26.6	10.6	18.6
Oral Medication Use^d^ (medications/month)			
0	18.1	8.0	13.1
1 to 2	38.0	33.4	35.7
3 to 5	25.9	31.8	28.9
> 5	17.9	26.8	22.4
Meat Consumption^e^ (servings/week)			
≤ 5	21.5	32.2	26.8
5 ≤ 8	42.9	42.2	42.5
> 8	35.6	25.6	30.6
Fruit & Vegetable Consumption^e^ (servings/week)			
≤ 19	40.4	23.4	31.9
19 ≤ 30	37.1	39.0	38.1
> 30	22.5	37.6	30.0
Bottled & Canned Drinks^e^ (drinks/week)			
≤ 5	35.1	46.3	40.7
5 ≤ 10	32.8	33.3	33.1
> 10	32.1	20.4	26.3
Urban vs. Rural Status			
Urban (CMA)	86.7	86.8	86.7
Rural (non-CMA)	13.3	13.2	13.3
Primary Source of Drinking Water			
Tap Water	67.2	69.3	68.2
Bottled Water	27.4	28.1	27.8
Other	5.3	2.6	4.0

*Abbreviations*: *CHMS* Canadian Health Measures Survey, *CMA* Census metropolitan area

Note: Percentages calculated based on overall analytic sample size (n = 4,259) and by sex (n_male_ = 2135; n_female_ = 2124). Percentages by covariate may not sum up to 100%. Missing categories not shown. Maximum missingness for any covariate ≤0.8%

^a^'Other' ethnicities include: Arab, Latin American, Indigenous, other racial or cultural origin

^b^Cycle-specific household income quartiles (CAD), rounded to base 100, calculated to control for income inflation between CHMS cycles. Imputation of missing income performed by CHMS

Cycle 1: Q1 (<35,000), Q2 (35,000 to <60,000), Q3 (60,000 to <95,000), Q4 (≥95,000)

Cycle 2: Q1 (<36,400), Q2 (36,400 to <62,000), Q3 (62,000 to <100,000), Q4 (≥100,000)

Cycle 5: Q1 (<44,000), Q2 (44,000 to <84,000), Q3 (84,000 to <128,000), Q4 (≥128,000)

Cycle 6: Q1 (<45,000), Q2 (45,000 to <80,000), Q3 (80,000 to <135,000), Q4 (≥135,000)

^c^Smoking status categories

Non-Smoker = <100 cigarettes over lifetime and not currently a smoker

Former Smoker = ≥100 cigarettes over lifetime and not currently a smoker

Current Smoker = Occasional or Daily Smoker (at time of CHMS interview)

^d^Number of unique oral prescription and over-the-counter medications taken in last month

^e^See Supplemental Table 3 for construction of diet-related variables

Summary statistics for uncorrected and creatinine-corrected phthalate metabolite concentrations are presented in Table 2. Concentrations were generally higher after creatinine-correction, though distributional patterns remained consistent. Moderate to strong positive Spearman correlations (Supplemental Fig. 3) were observed between most metabolites (with DF ≥ 70%), particularly across DEHP-devolving metabolites (ρ ≈ 0.8–0.9). Correlations were invariably attenuated after creatinine-correction. Almost all participants had detectable levels of six metabolites (range: 98–100%), including all three DEHP-devolving metabolites (MEOHP, MEHHP, and MEHP). Concentrations of most phthalate metabolites decreased substantially across CHMS cycles, particularly for DEHP-devolving metabolites.

**Table 2:** Summary statistics for uncorrected and creatinine-corrected urinary phthalate concentrations, CHMS, 2007–2019

Parent Phthalate	Measured Phthalate Metabolite	# of Observations (*n*_total_ = 4,259)	*n* > LOD	DF %	Uncorrected concentrations, µg/L			Creatinine-corrected concentrations, µg/g creatinine		
					GM (GSD)	Median	IQR	GM (GSD)	Median	IQR
DMP	MMP	4,197	2,280	54.32	-	3.54	1.90, 3.54	-	2.75	1.55, 5.12
DEP	MEP	4,257	4,239	99.58	30.66 (4.53)	28.00	11.00, 75.00	32.59 (3.88)	28.21	12.16, 74.86
DBP	MnBP	4,238	4,233	99.88	14.62 (2.84)	15.00	7.67, 28.00	15.49 (2.20)	14.22	9.33, 23.46
DCHP	MCHP	4,243	451	10.63	-	0.18	0.14, 0.18	-	0.14	0.09, 0.29
DEHP	MEOHP	4,254	4,248	99.86	5.22 (3.39)	5.00	2.36, 11.00	5.54 (2.88)	4.97	2.66, 10.24
DEHP	MEHHP	4,258	4,253	99.88	8.42 (3.50)	7.90	3.70, 18.00	8.95 (2.96)	7.96	4.18, 17.08
DEHP	MEHP	4,188	4,144	98.95	1.55 (3.54)	1.50	0.68, 3.40	1.63 (3.18)	1.54	0.76, 3.21
DiNP	MiNP	4,246	872	20.54	-	0.26	0.21, 0.28	-	0.26	0.15, 0.52
DnOP	MOP	4,257	75	1.76	-	0.21	0.11, 0.49	-	0.19	0.09, 0.42
DnOP	MCPP	4,168	3,724	89.35	0.95 (3.63)	1.00	0.41, 2.16	1.00 (2.84)	0.92	0.49, 1.88
BzBP	MBzP	4,246	4,180	98.45	4.81 (3.92)	4.80	2.00, 12.00	5.11 (3.15)	5.03	2.33, 10.97
-	∑LMWP^a^	4,160	-	-	272.39 (3.45)	248.16	121.98, 552.50	287.45 (2.91)	250.01	136.10, 520.66
-	∑HMWP^a^	4,070	-	-	91.99 (3.18)	89.91	42.75, 192.84	96.25 (2.66)	89.98	47.62, 174.98
-	∑DEHP^a^	4,183	-	-	51.92 (3.38)	49.17	23.26, 108.85	54.72 (2.89)	48.60	25.85, 102.68
-	∑Total^a^	3,984	-	-	414.26 (3.23)	387.97	191.02, 831.01	430.11 (2.72)	384.78	213.49, 763.05

*Abbreviations*: – no data, *CHMS* Canadian Health Measures Survey, *MMP* Mono-methyl phthalate, *MEP* Mono-ethyl phthalate, *MnBP* Mono-n-butyl phthalate, *MCHP* Mono-cyclohexyl phthalate, *MEOHP* Mono-(2-ethyl-5-oxohexyl) phthalate, *MEHHP* Mono-(2-ethyl-5-hydroxyhexyl) phthalate, *MEHP* Mono-(2-ethylhexyl) phthalate, *MiNP* Mono-isononyl phthalate, *MOP* Mono-octyl phthalate, *MCPP* Mono-(3-carboxypropyl) phthalate, *MBzP* Mono-benzyl phthalate, *LMWP* Low molecular weight phthalate, *HMWP* High molecular weight phthalate, *DEHP* Di(2-ethylhexyl) phthalate, *DMP* Dimethyl phthalate, *DEP* Diethyl phthalate, *DBP* Dibutyl phthalate, *DCHP* Dicyclohexyl phthalate, *DEHP* Di(2-ethylhexyl) phthalate, *DiNP* Diisononyl phthalate, *DnOP* Di-n-octyl phthalate, *BzBP* Benzyl butyl phthalate, *LOD* Limit of detection, *DF %* Detection frequency percentage, *GM* Geometric mean, *GSD* Geometric standard deviation, *IQR* Interquartile range

Adapted from Allotey et al., 2021 [4]

LODs by metabolite and cycle are presented in Supplemental Table 2

^a^Units for ∑LMWP, ∑HMWP, ∑DEHP, and ∑Total concentrations: Uncorrected = nmol/L; Creatinine-corrected = nmol/g creatinine

∑LMWP = Molar sum of MMP + MEP + MnBP + MCHP

∑HMWP = Molar sum of MEOHP + MEHHP + MEHP + MiNP + MOP + MCPP + MBzP

∑DEHP = Molar sum of MEOHP + MEHHP + MEHP

∑Total = Molar sum of MMP + MEP + MnBP + MCHP + MEOHP + MEHHP + MEHP + MiNP + MOP + MCPP + MBzP

In adjusted models, females had considerably higher GM concentrations than males across all measured metabolites, with the largest contrast for MEP, which was 39% (95% CI: 29, 50%) higher in females (Table 3). Concentrations generally increased modestly with age, with elevated GMRs observed among older adults (60–79 years) for most metabolites. Asian participants exhibited higher levels of MnBP, MEOHP, MEHHP, MEHP, ∑DEHP, and ∑LMWP, while Black participants showed elevated concentrations for MEP, MEHP, and ∑LMWP relative to White participants. Higher fruit and vegetable consumption was associated with a monotonic increase in concentrations of most metabolites, particularly among DEHP-devolving metabolites, whereas greater consumption of bottled and canned drinks was linked to slightly elevated MEP and ∑LMWP. Household income was inversely associated with MEP, MnBP, MBzP, ∑LMWP, and ∑HMWP. Cigarette smoking showed modest positive associations with MBzP. Other factors were not meaningfully associated with metabolite concentrations.

**Table 3:** Associations between participant characteristics with creatinine-corrected phthalate concentrations (DF ≥ 70%) and summary groups, CHMS, 2007–2019

Characteristic	GMR^c^ (95% CI)										
	MEP^a^ (*n* = 4,239)	MnBP^a^ (*n* = 4,220)	MEOHP^a^ (*n* = 4,236)	MEHHP^a^ (*n* = 4,240)	MEHP^a^ (*n* = 4,170)	MCPP^a^ (*n* = 4,151)	MBzP^a^ (*n* = 4,228)	∑LMWP^b^ (*n* = 4,143)	∑HMWP^b^ (*n* = 4,053)	∑DEHP^b^ (*n* = 4,165)	∑Total^b^ (*n* = 3,968)
CHMS Cycle (Years)											
Cycle 1 (2007–2009)	Ref.	Ref.	Ref.	Ref.	Ref.	Ref.	Ref.	Ref.	Ref.	Ref.	Ref.
Cycle 2 (2009–2011)	0.61 (0.54, 0.68)	0.77 (0.72, 0.82)	0.42 (0.39, 0.45)	0.43 (0.40, 0.46)	0.42 (0.38, 0.45)	1.12 (1.03, 1.22)	0.70 (0.64, 0.76)	0.66 (0.61, 0.72)	0.50 (0.47, 0.54)	0.43 (0.40, 0.46)	0.58 (0.54, 0.63)
Cycle 5 (2016–2017)	0.27 (0.24, 0.30)	0.47 (0.44, 0.51)	0.20 (0.18, 0.21)	0.18 (0.16, 0.19)	0.22 (0.20, 0.24)	0.44 (0.41, 0.48)	0.35 (0.32, 0.39)	0.33 (0.30, 0.36)	0.25 (0.23, 0.26)	0.19 (0.18, 0.20)	0.29 (0.27, 0.31)
Cycle 6 (2018–2019)	0.25 (0.22, 0.28)	0.48 (0.45, 0.52)	0.18 (0.16, 0.19)	0.16 (0.15, 0.18)	0.20 (0.18, 0.22)	0.40 (0.36, 0.44)	0.27 (0.25, 0.30)	0.31 (0.29, 0.35)	0.21 (0.20, 0.23)	0.17 (0.16, 0.19)	0.27 (0.24, 0.29)
Sex											
Male	Ref.	Ref.	Ref.	Ref.	Ref.	Ref.	Ref.	Ref.	Ref.	Ref.	Ref.
Female	1.39 (1.29, 1.50)	1.36 (1.30, 1.42)	1.28 (1.21, 1.34)	1.19 (1.13, 1.25)	1.16 (1.09, 1.23)	1.20 (1.13, 1.27)	1.33 (1.25, 1.41)	1.36 (1.28, 1.44)	1.27 (1.21, 1.33)	1.21 (1.15, 1.28)	1.32 (1.25, 1.39)
Age at Clinic Visit, years											
20–39	Ref.	Ref.	Ref.	Ref.	Ref.	Ref.	Ref.	Ref.	Ref.	Ref.	Ref.
40–59	1.12 (1.03, 1.22)	1.03 (0.98, 1.08)	1.01 (0.95, 1.06)	1.03 (0.97, 1.09)	0.90 (0.84, 0.96)	1.05 (0.98, 1.12)	0.92 (0.86, 0.98)	1.09 (1.02, 1.16)	0.98 (0.93, 1.03)	1.01 (0.95, 1.06)	1.05 (0.99, 1.12)
60–79	1.50 (1.35, 1.66)	1.19 (1.12, 1.26)	1.16 (1.08, 1.24)	1.16 (1.09, 1.25)	0.82 (0.76, 0.89)	1.10 (1.01, 1.20)	0.87 (0.80, 0.94)	1.35 (1.24, 1.46)	1.02 (0.95, 1.09)	1.13 (1.05, 1.21)	1.25 (1.16, 1.35)
Ethnicity											
White	Ref.	Ref.	Ref.	Ref.	Ref.	Ref.	Ref.	Ref.	Ref.	Ref.	Ref.
Black	2.32 (1.82, 2.96)	1.08 (0.94, 1.25)	1.05 (0.90, 1.23)	1.07 (0.91, 1.26)	1.44 (1.20, 1.74)	0.76 (0.63, 0.91)	0.67 (0.55, 0.81)	1.87 (1.55, 2.26)	0.91 (0.79, 1.06)	1.12 (0.95, 1.31)	1.64 (1.38, 1.94)
Asian	1.06 (0.94, 1.20)	1.42 (1.32, 1.52)	1.14 (1.05, 1.23)	1.13 (1.04, 1.23)	1.51 (1.38, 1.67)	0.95 (0.87, 1.05)	0.64 (0.58, 0.71)	1.29 (1.17, 1.42)	1.00 (0.92, 1.08)	1.17 (1.08, 1.27)	1.22 (1.12, 1.33)
Other^d^	1.24 (1.08, 1.42)	1.11 (1.03, 1.20)	1.01 (0.92, 1.10)	1.01 (0.93, 1.11)	1.19 (1.07, 1.32)	0.93 (0.84, 1.04)	0.93 (0.83, 1.03)	1.20 (1.08, 1.33)	0.97 (0.89, 1.05)	1.03 (0.94, 1.12)	1.12 (1.02, 1.24)
Total Household Income^e^ (Quartiles)											
Q1 (lowest income)	Ref.	Ref.	Ref.	Ref.	Ref.	Ref.	Ref.	Ref.	Ref.	Ref.	Ref.
Q2	0.97 (0.87, 1.08)	0.92 (0.86, 0.98)	0.98 (0.91, 1.05)	0.99 (0.93, 1.07)	1.05 (0.96, 1.14)	0.99 (0.91, 1.08)	0.86 (0.79, 0.94)	0.95 (0.87, 1.03)	0.95 (0.89, 1.01)	0.99 (0.93, 1.07)	0.95 (0.88, 1.03)
Q3	0.83 (0.75, 0.93)	0.88 (0.82, 0.93)	0.99 (0.92, 1.06)	1.00 (0.93, 1.07)	1.02 (0.94, 1.11)	0.94 (0.87, 1.03)	0.81 (0.75, 0.89)	0.84 (0.77, 0.91)	0.92 (0.86, 0.98)	0.99 (0.92, 1.07)	0.87 (0.80, 0.93)
Q4 (highest income)	0.90 (0.81, 1.01)	0.80 (0.75, 0.85)	0.93 (0.86, 1.00)	0.94 (0.87, 1.01)	0.98 (0.90, 1.06)	0.99 (0.91, 1.08)	0.76 (0.69, 0.83)	0.86 (0.79, 0.94)	0.87 (0.82, 0.94)	0.94 (0.87, 1.01)	0.87 (0.80, 0.94)
Smoking Status^f^											
Non-Smoker / Former Smoker	Ref.	Ref.	Ref.	Ref.	Ref.	Ref.	Ref.	Ref.	Ref.	Ref.	Ref.
Current Smoker	1.04 (0.95, 1.15)	1.04 (0.98, 1.10)	0.98 (0.92, 1.04)	0.96 (0.90, 1.03)	0.99 (0.92, 1.07)	0.95 (0.89, 1.03)	1.14 (1.05, 1.23)	1.04 (0.96, 1.12)	1.03 (0.97, 1.09)	0.97 (0.91, 1.04)	1.04 (0.97, 1.11)
Meat Consumption^g^(servings/week)											
< 5	Ref.	Ref.	Ref.	Ref.	Ref.	Ref.	Ref.	Ref.	Ref.	Ref.	Ref.
5 < 8	0.91 (0.83, 1.00)	0.92 (0.88, 0.97)	0.95 (0.89, 1.01)	0.96 (0.91, 1.02)	1.01 (0.94, 1.08)	0.89 (0.83, 0.96)	1.05 (0.98, 1.13)	0.92 (0.86, 0.99)	0.98 (0.93, 1.04)	0.96 (0.91, 1.02)	0.94 (0.88, 1.01)
> 8	0.85 (0.77, 0.94)	0.93 (0.87, 0.98)	0.91 (0.85, 0.97)	0.91 (0.86, 0.98)	0.95 (0.88, 1.03)	0.90 (0.83, 0.97)	1.02 (0.94, 1.11)	0.90 (0.83, 0.97)	0.94 (0.88, 1.00)	0.92 (0.86, 0.98)	0.92 (0.85, 0.98)
Seafood Consumption^g^ (servings/week)											
0	Ref.	Ref.	Ref.	Ref.	Ref.	Ref.	Ref.	Ref.	Ref.	Ref.	Ref.
0 < 1	1.04 (0.93, 1.17)	0.96 (0.90, 1.03)	0.93 (0.86, 1.00)	0.94 (0.88, 1.02)	0.97 (0.89, 1.06)	0.94 (0.86, 1.03)	0.97 (0.89, 1.06)	1.01 (0.92, 1.10)	0.96 (0.89, 1.03)	0.94 (0.88, 1.01)	0.98 (0.91, 1.06)
> 1	1.04 (0.93, 1.16)	0.96 (0.90, 1.03)	0.95 (0.88, 1.02)	0.97 (0.90, 1.05)	1.06 (0.97, 1.15)	0.96 (0.88, 1.04)	0.84 (0.77, 0.92)	1.00 (0.92, 1.09)	0.95 (0.88, 1.01)	0.98 (0.91, 1.05)	0.99 (0.91, 1.07)
Dairy Consumption^g^(servings/week)											
< 8	Ref.	Ref.	Ref.	Ref.	Ref.	Ref.	Ref.	Ref.	Ref.	Ref.	Ref.
8 < 14	1.11 (1.01, 1.22)	1.03 (0.98, 1.09)	0.99 (0.94, 1.06)	0.99 (0.93, 1.05)	0.98 (0.91, 1.05)	1.05 (0.97, 1.13)	1.01 (0.94, 1.09)	1.07 (0.99, 1.15)	0.99 (0.94, 1.05)	0.99 (0.93, 1.05)	1.06 (0.99, 1.13)
> 14	1.05 (0.96, 1.16)	1.03 (0.98, 1.09)	0.95 (0.90, 1.02)	0.96 (0.90, 1.02)	0.93 (0.87, 1.01)	1.02 (0.95, 1.10)	1.03 (0.95, 1.11)	1.03 (0.96, 1.11)	0.98 (0.92, 1.04)	0.95 (0.89, 1.01)	1.02 (0.95, 1.09)
Grain & Rice Consumption^g^(servings/week)											
< 12	Ref.	Ref.	Ref.	Ref.	Ref.	Ref.	Ref.	Ref.	Ref.	Ref.	Ref.
12 < 16	0.93 (0.85, 1.02)	1.06 (1.00, 1.11)	1.04 (0.98, 1.10)	1.02 (0.97, 1.09)	1.08 (1.01, 1.16)	0.99 (0.92, 1.06)	1.01 (0.94, 1.09)	0.99 (0.92, 1.06)	1.04 (0.98, 1.10)	1.04 (0.98, 1.10)	1.00 (0.94, 1.06)
> 16	0.96 (0.87, 1.06)	1.08 (1.02, 1.14)	0.99 (0.93, 1.06)	0.99 (0.93, 1.06)	1.06 (0.98, 1.14)	1.03 (0.95, 1.11)	1.01 (0.94, 1.10)	1.01 (0.94, 1.10)	1.01 (0.95, 1.07)	1.00 (0.94, 1.07)	0.99 (0.93, 1.07)
Fruit & Vegetable Consumption^g^ (servings/week)											
< 19	Ref.	Ref.	Ref.	Ref.	Ref.	Ref.	Ref.	Ref.	Ref.	Ref.	Ref.
19 < 30	1.02 (0.93, 1.12)	1.04 (0.98, 1.09)	1.09 (1.03, 1.16)	1.08 (1.02, 1.15)	1.09 (1.02, 1.17)	1.01 (0.94, 1.09)	0.97 (0.90, 1.05)	1.03 (0.96, 1.10)	1.04 (0.99, 1.10)	1.09 (1.03, 1.16)	1.03 (0.97, 1.10)
> 30	1.05 (0.95, 1.16)	1.07 (1.01, 1.13)	1.16 (1.08, 1.23)	1.15 (1.07, 1.23)	1.19 (1.10, 1.29)	1.05 (0.97, 1.14)	0.95 (0.87, 1.03)	1.08 (0.99, 1.16)	1.07 (1.01, 1.14)	1.16 (1.08, 1.24)	1.07 (1.00, 1.15)
Bottled & Canned Drinks^g^(drinks/week)											
< 5	Ref.	Ref.	Ref.	Ref.	Ref.	Ref.	Ref.	Ref.	Ref.	Ref.	Ref.
5 < 10	1.08 (0.99, 1.18)	1.01 (0.96, 1.07)	0.96 (0.90, 1.01)	0.97 (0.92, 1.03)	0.92 (0.86, 0.99)	1.00 (0.93, 1.07)	1.01 (0.94, 1.09)	1.06 (0.99, 1.13)	0.96 (0.91, 1.02)	0.96 (0.90, 1.01)	1.04 (0.97, 1.10)
> 10	1.13 (1.02, 1.25)	0.96 (0.91, 1.02)	1.03 (0.96, 1.10)	1.05 (0.98, 1.12)	0.94 (0.87, 1.02)	1.04 (0.96, 1.12)	1.01 (0.94, 1.10)	1.08 (1.00, 1.17)	1.02 (0.96, 1.09)	1.03 (0.96, 1.10)	1.09 (1.01, 1.17)
Physical Activity (Tertiles)											
T1 (least active)	Ref.	Ref.	Ref.	Ref.	Ref.	Ref.	Ref.	Ref.	Ref.	Ref.	Ref.
T2	0.99 (0.90, 1.08)	1.00 (0.95, 1.06)	1.02 (0.96, 1.08)	1.02 (0.96, 1.08)	1.03 (0.96, 1.11)	0.99 (0.92, 1.06)	0.99 (0.92, 1.07)	1.00 (0.93, 1.08)	1.01 (0.96, 1.07)	1.02 (0.96, 1.08)	1.00 (0.94, 1.07)
T3 (most active)	0.93 (0.85, 1.02)	0.99 (0.94, 1.05)	0.98 (0.92, 1.04)	0.98 (0.93, 1.05)	1.04 (0.96, 1.12)	0.98 (0.91, 1.06)	0.96 (0.89, 1.03)	0.96 (0.89, 1.03)	0.97 (0.92, 1.03)	0.99 (0.93, 1.05)	0.95 (0.89, 1.02)
Primary Source of Drinking Water											
Tap Water	Ref.	Ref.	Ref.	Ref.	Ref.	Ref.	Ref.	Ref.	Ref.	Ref.	Ref.
Bottled Water	1.01 (0.93, 1.10)	0.96 (0.91, 1.00)	0.99 (0.94, 1.05)	0.96 (0.91, 1.02)	0.95 (0.89, 1.01)	0.98 (0.92, 1.05)	0.96 (0.89, 1.03)	0.99 (0.93, 1.06)	0.96 (0.91, 1.02)	0.97 (0.91, 1.02)	0.98 (0.93, 1.04)
Other	0.95 (0.79, 1.15)	1.06 (0.95, 1.19)	0.93 (0.82, 1.06)	0.94 (0.83, 1.06)	0.96 (0.83, 1.11)	0.86 (0.75, 1.00)	1.15 (0.99, 1.34)	1.00 (0.86, 1.16)	0.97 (0.86, 1.09)	0.93 (0.82, 1.06)	0.96 (0.84, 1.10)

*Abbreviations*: *CHMS* Canadian Health Measures Survey, *GMR* Geometric mean ratio, *CI* Confidence interval, *DF* Detection frequency, *MMP* Mono-methyl phthalate, *MEP* Mono-ethyl phthalate, *MnBP* Mono-n-butyl phthalate, *MCHP* Mono-cyclohexyl phthalate, *MEOHP* Mono-(2-ethyl-5-oxohexyl) phthalate, *MEHHP* Mono-(2-ethyl-5-hydroxyhexyl) phthalate, *MEHP* Mono-(2-ethylhexyl) phthalate, *MiNP* Mono-isononyl phthalate, *MOP* Mono-octyl phthalate, *MCPP* Mono-(3-carboxypropyl) phthalate, *MBzP* Mono-benzyl phthalate, *LMWP* Low molecular weight phthalate, *HMWP* High molecular weight phthalate, *DEHP* Di(2-ethylhexyl) phthalate

^a^Based on concentration units of µg/g creatinine

^b^Based on concentration units of nmol/g creatinine

∑LMWP = Molar sum of MMP + MEP + MnBP + MCHP

∑HMWP = Molar sum of MEOHP + MEHHP + MEHP + MiNP + MOP + MCPP + MBzP

∑DEHP = Molar sum of MEOHP + MEHHP + MEHP

∑Total = Molar sum of MMP + MEP + MnBP + MCHP + MEOHP + MEHHP + MEHP + MiNP + MOP + MCPP + MBzP

^c^Least-squares GMRs computed from multiple linear regression models adjusting for all variables listed in the table. Model sizes (row 1) assume a complete-case analysis approach

^d^‘Other’ ethnicities include: Arab, Latin American, Indigenous, other racial or cultural origin

^e^Cycle-specific household income quartiles (CAD), calculated to control for income inflation between CHMS cycles. Imputation of missing income performed by CHMS

Cycle 1: Q1 (< 35,000), Q2 (35,000 to < 60,000), Q3 (60,000 to < 95,000), Q4 (≥ 95,000)

Cycle 2: Q1 (< 36,400), Q2 (36,400 to < 62,000), Q3 (62,000 to < 100,000), Q4 (≥ 100,000)

Cycle 5: Q1 (< 44,000), Q2 (44,000 to < 84,000), Q3 (84,000 to < 128,000), Q4 (≥ 128,000)

Cycle 6: Q1 (< 45,000), Q2 (45,000 to < 80,000), Q3 (80,000 to < 135,000), Q4 (≥ 135,000)

^f^Smoking status categories

Non-Smoker: <100 cigarettes over lifetime and not currently a smoker

Former Smoker: ≥100 cigarettes over lifetime and not currently a smoker

Current Smoker: Occasional or Daily Smoker

^g^See Supplemental Table 3 for construction of diet-related variables

**Fig. 1:**
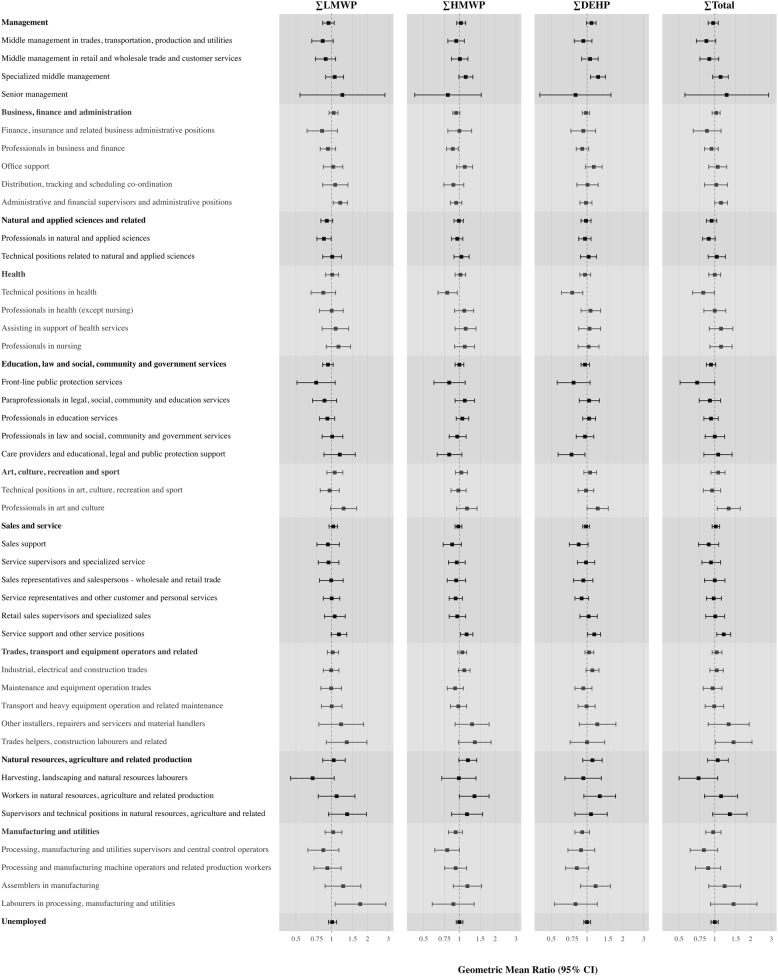
Associations between occupation with creatinine-corrected phthalate summary group concentrations, CHMS, 2007–2019. Abbreviations: GMR, Geometric mean ratio; CI, Confidence interval; MEP, Mono-ethyl phthalate; MnBP, Mono-n-butyl phthalate; MEOHP, Mono-(2-ethyl-5-oxohexyl) phthalate; MEHHP, Mono-(2-ethyl-5-hydroxyhexyl) phthalate; MEHP, Mono-(2-ethylhexyl) phthalate; MCPP, Mono-(3-carboxypropyl) phthalate; MBzP, Mono-benzyl phthalate; MMP, Mono-methyl phthalate; MCHP, Mono-cyclohexyl phthalate; MiNP, Mono-isononyl phthalate; MOP, Mono-octyl phthalate; LMWP, Low molecular weight phthalate; HMWP, High molecular weight phthalate; DEHP, Di(2-ethylhexyl) phthalate. Broad-level occupations = bolded; Major-level occupations = unbolded. Estimate based on geometric mean concentration units of nmol/g creatinine. ∑LMWP = Molar sum of MMP + MEP + MnBP + MCHP. ∑HMWP = Molar sum of MEOHP + MEHHP + MEHP + MiNP + MOP + MCPP + MBzP. ∑DEHP = Molar sum of MEOHP + MEHHP + MEHP. ∑Total = Molar Sum of MMP + MEP + MnBP + MCHP + MEOHP + MEHHP + MEHP + MiNP + MOP + MCPP + MBzP. Least-squares GMRs computed by multivariable linear regression, using occupational terms as binary indicators, adjusting for: age (continuous), CHMS cycle, ethnicity, household income, smoking status, six dietary consumptions groups (meat and alternatives, dairy, grains, fruits and vegetables, seafood, bottled/canned beverages), drinking water type, and physical activity

In occupational models, workers in *trades*,* transport and equipment operation* showed elevated GMRs for MCPP (1.15; 95% CI: 1.04, 1.27) and MEHP (1.10; 95% CI: 0.99, 1.22) (Fig. 1, Supplemental Table 6). Within this broad-level NOC group, *industrial*,* electrical*,* and construction trades* exhibited increased concentrations for DEHP-devolving metabolites, particularly MEHP (1.22; 95% CI: 1.05, 1.41). *Trades helpers and construction labourers* had elevated MnBP, MCPP, and ∑Total (GMR range: 1.41–1.80), and *other installers*,* repairers and servicers* and *material handlers* had GMRs > 1 across all metabolites (range: 1.15–1.33), though estimates were comparatively imprecise with wider confidence intervals.

Workers in *natural resources*,* agriculture and related production* also exhibited modestly increased GMRs across most metabolites, especially for MCPP (1.44; 95% CI: 1.15, 1.79) and MBzP (1.32; 95% CI: 1.05, 1.66). Notable major-level associations were observed for *workers in natural resources and agriculture* (GMR_MCPP_ = 1.64; 95% CI: 1.15, 2.33; GMR_∑HMWP_ = 1.34; 95% CI: 1.00, 1.78) and for *harvesting*,* landscaping and natural resource labourers* (GMR_MCPP_ = 1.58; 95% CI: 1.03, 2.41).

Across *health* occupations, broad-level estimates generally suggested little association, except for MBzP (1.26; 95% CI: 1.11, 1.43) driven by elevated GMRs for *nursing* and *non-nursing professionals*. GMRs for workers in *manufacturing and utilities* were largely null at the broad-level, although *assemblers in manufacturing* and *labourers in processing*,* manufacturing*,* and utilities* had elevated but imprecise estimates, including ∑LMWP (1.74; 95% CI: 1.07, 2.86) for the latter. Additional major-level occupations with modestly increased estimates included *professionals in art and culture* (GMR range: 0.97–1.31) and *service support and other service occupations* (GMR range: 1.11–1.19). The GMRs for *service support and other service occupations* were among the most precise (confidence limit ratio < 1.5) major-level estimates, reflecting this group’s large relative sample size.

Analyses of metabolites with low DFs (< 70%) identified several occupations with increased odds of detection (Supplemental Table 7). Workers in *natural resources*,* agriculture and related production* had the highest odds of MCHP detection, with increasing odds from 2.90 at the broad level to 3.86 among supervisors and technical positions. Despite wide CIs, *technical occupations in art*,* culture*,* recreation and sport* showed increased odds for MiNP (1.91; 95% CI: 0.85, 4.02) and MOP (2.77; 95% CI: 1.00, 6.47). Other occupations with notable associations included *office support and administrative* subgroups for MCHP and MiNP, and *sales and service* roles for MOP. Variability in CI width reflected small occupational sample sizes, with some groups having few workers above their respective LODs.

Sex differences were observed in the magnitude and pattern of several occupational associations (Table 4, Supplemental Tables 8 and 9). Elevated concentrations among males in *industrial*,* electrical*,* and construction trades* (e.g., GMR_MEHP_ = 1.23; 95% CI: 1.06, 1.44) and *trades helpers and construction labourers* (e.g., GMR_MCPP_ = 1.85; 95% CI: 1.24, 2.76) were not seen among the small number of females in these occupations. The ∑LMWP estimate among male *labourers in processing*,* manufacturing and utilities* (2.35; 95% CI: 1.25, 4.42) was also markedly higher than the corresponding estimate for females (0.99; 95% CI: 0.44, 2.22). Conversely, females in *office support* occupations had elevated GMRs for MEOHP (1.19; 95% CI: 1.00, 1.40) and MCPP (1.24; 95% CI: 1.01, 1.52), which were not observed among males. For workers in *natural resources*,* agriculture and related production*, elevated GMRs in males were driven by MCPP (1.43; 95% CI: 1.13, 1.82) and MBzP (1.34; 95% CI: 1.04, 1.72), whereas in females, elevations were primarily for DEHP-devolving metabolites, such as MEHHP (1.56; 95% CI: 1.01, 2.40). Females in *service support and other service* positions also showed increased concentrations for ∑DEHP and ∑Total (GMR range: 1.27–1.30), while estimates for males in these occupations were attenuated (GMR range: 1.02–1.11).

**Table 4:** Associations between occupation with creatinine-corrected phthalate summary group concentrations, by sex, CHMS, 2007–2019

Occupation (Broad = Bold; Major = Unbolded)	GMR^b^ (95% CI)							
	Males (*n* = 2,135)				Females (*n* = 2,124)			
	∑LMWP^a^	∑HMWP^a^	∑DEHP^a^	∑Total^a^	∑LMWP^a^	∑HMWP^a^	∑DEHP^a^	∑Total^a^
**Management**	0.92 (0.79, 1.06)	1.04 (0.93, 1.17)	1.07 (0.95, 1.21)	0.95 (0.83, 1.08)	0.99 (0.83, 1.18)	1.03 (0.90, 1.19)	1.13 (0.98, 1.32)	1.02 (0.87, 1.20)
Senior management	1.25 (0.54, 2.92)	0.81 (0.42, 1.56)	0.82 (0.41, 1.65)	1.29 (0.56, 2.99)	-	-	-	-
Specialized middle management	1.06 (0.84, 1.33)	1.29 (1.09, 1.54)	1.42 (1.18, 1.72)	1.19 (0.97, 1.46)	1.05 (0.80, 1.37)	0.95 (0.77, 1.17)	1.03 (0.82, 1.29)	1.01 (0.80, 1.28)
Middle management in retail and wholesale trade and customer services	0.85 (0.63, 1.16)	0.83 (0.65, 1.05)	0.82 (0.64, 1.06)	0.78 (0.58, 1.03)	0.94 (0.73, 1.22)	1.18 (0.96, 1.45)	1.29 (1.04, 1.60)	1.04 (0.83, 1.31)
Middle management in trades, transportation, production and utilities	0.82 (0.65, 1.03)	0.96 (0.80, 1.15)	0.94 (0.78, 1.13)	0.83 (0.67, 1.02)	0.97 (0.60, 1.57)	0.87 (0.58, 1.29)	0.95 (0.62, 1.46)	0.98 (0.63, 1.53)
**Business, finance and administration**	0.99 (0.86, 1.15)	0.89 (0.79, 0.99)	0.89 (0.79, 1.01)	0.97 (0.85, 1.11)	1.07 (0.96, 1.19)	0.97 (0.89, 1.05)	1.03 (0.94, 1.13)	1.06 (0.96, 1.17)
Professionals in business and finance	0.88 (0.71, 1.08)	0.83 (0.70, 0.97)	0.83 (0.70, 0.99)	0.85 (0.70, 1.02)	1.01 (0.82, 1.25)	0.92 (0.77, 1.09)	1.00 (0.84, 1.20)	1.05 (0.87, 1.27)
Administrative and financial supervisors and administrative positions	1.41 (1.04, 1.91)	1.01 (0.80, 1.27)	0.96 (0.76, 1.23)	1.33 (1.01, 1.74)	1.11 (0.95, 1.30)	0.92 (0.81, 1.04)	0.97 (0.85, 1.11)	1.07 (0.93, 1.23)
Finance, insurance and related business administrative positions	0.70 (0.39, 1.24)	1.01 (0.64, 1.61)	0.87 (0.54, 1.40)	0.74 (0.43, 1.25)	0.90 (0.64, 1.26)	0.97 (0.74, 1.27)	0.93 (0.70, 1.24)	0.91 (0.67, 1.24)
Office support	0.81 (0.45, 1.47)	0.90 (0.57, 1.42)	0.94 (0.58, 1.55)	0.92 (0.54, 1.57)	1.07 (0.88, 1.30)	1.15 (0.98, 1.35)	1.19 (1.00, 1.41)	1.09 (0.91, 1.30)
Distribution, tracking and scheduling co-ordination	1.09 (0.79, 1.49)	0.91 (0.71, 1.16)	0.99 (0.76, 1.29)	1.04 (0.79, 1.38)	1.05 (0.71, 1.55)	0.86 (0.62, 1.19)	1.04 (0.74, 1.47)	0.98 (0.69, 1.40)
**Natural and applied sciences and related**	0.88 (0.76, 1.00)	1.02 (0.91, 1.13)	1.00 (0.89, 1.11)	0.92 (0.81, 1.04)	0.99 (0.79, 1.23)	0.92 (0.77, 1.10)	0.93 (0.77, 1.12)	1.00 (0.82, 1.23)
Professionals in natural and applied sciences	0.88 (0.74, 1.03)	1.00 (0.88, 1.13)	0.97 (0.85, 1.11)	0.90 (0.78, 1.04)	0.81 (0.61, 1.08)	0.84 (0.67, 1.06)	0.91 (0.71, 1.15)	0.86 (0.67, 1.11)
Technical positions related to natural and applied sciences	0.90 (0.72, 1.12)	1.05 (0.88, 1.25)	1.05 (0.87, 1.26)	0.97 (0.79, 1.18)	1.29 (0.93, 1.81)	1.04 (0.78, 1.37)	0.97 (0.73, 1.30)	1.25 (0.92, 1.70)
**Health**	1.17 (0.90, 1.52)	1.08 (0.87, 1.33)	1.10 (0.88, 1.37)	1.17 (0.92, 1.50)	0.95 (0.82, 1.09)	1.00 (0.89, 1.12)	0.92 (0.81, 1.03)	0.94 (0.83, 1.07)
Professionals in nursing	2.35 (0.91, 6.04)	0.80 (0.29, 2.25)	0.91 (0.30, 2.76)	2.21 (0.68, 7.21)	1.04 (0.82, 1.32)	1.10 (0.90, 1.34)	1.01 (0.82, 1.24)	1.06 (0.85, 1.31)
Professionals in health (except nursing)	1.13 (0.78, 1.64)	1.31 (0.98, 1.75)	1.28 (0.94, 1.73)	1.28 (0.91, 1.80)	0.91 (0.68, 1.21)	0.97 (0.77, 1.24)	0.95 (0.74, 1.22)	0.84 (0.65, 1.10)
Technical positions in health	0.92 (0.58, 1.46)	0.74 (0.51, 1.06)	0.77 (0.52, 1.12)	0.81 (0.53, 1.24)	0.83 (0.63, 1.09)	0.81 (0.65, 1.01)	0.74 (0.58, 0.93)	0.80 (0.63, 1.02)
Assisting in support of health services	1.61 (0.74, 3.49)	1.43 (0.79, 2.60)	1.60 (0.85, 3.04)	1.73 (0.87, 3.43)	1.01 (0.77, 1.32)	1.10 (0.89, 1.37)	1.01 (0.80, 1.27)	1.06 (0.84, 1.35)
**Education, law and social, community and government services**	0.94 (0.79, 1.12)	0.84 (0.73, 0.97)	0.82 (0.71, 0.95)	0.90 (0.77, 1.05)	0.93 (0.83, 1.05)	1.11 (1.00, 1.23)	1.06 (0.95, 1.18)	0.96 (0.86, 1.07)
Professionals in education services	0.90 (0.70, 1.16)	0.87 (0.71, 1.07)	0.86 (0.69, 1.06)	0.88 (0.70, 1.11)	0.92 (0.77, 1.10)	1.17 (1.01, 1.36)	1.14 (0.98, 1.33)	0.96 (0.81, 1.13)
Professionals in law and social, community and government services	1.15 (0.83, 1.60)	0.88 (0.68, 1.13)	0.87 (0.66, 1.13)	1.06 (0.79, 1.42)	0.91 (0.70, 1.17)	1.03 (0.83, 1.29)	1.03 (0.82, 1.28)	0.93 (0.73, 1.19)
Paraprofessionals in legal, social, community and education services	0.77 (0.42, 1.40)	1.36 (0.86, 2.15)	1.34 (0.82, 2.20)	0.95 (0.56, 1.61)	0.92 (0.72, 1.18)	1.07 (0.88, 1.31)	1.01 (0.82, 1.26)	0.93 (0.74, 1.16)
Front-line public protection services	0.75 (0.51, 1.12)	0.75 (0.55, 1.02)	0.72 (0.52, 1.01)	0.68 (0.47, 0.97)	0.70 (0.20, 2.49)	10.24 (2.42, 43.31)	1.91 (0.64, 5.68)	3.38 (0.70, 16.41)
Care providers and educational, legal and public protection support	2.19 (0.85, 5.64)	0.33 (0.16, 0.69)	0.28 (0.13, 0.61)	1.60 (0.69, 3.70)	1.09 (0.79, 1.49)	0.96 (0.74, 1.24)	0.86 (0.66, 1.14)	1.03 (0.77, 1.36)
**Art, culture, recreation and sport**	1.03 (0.83, 1.28)	1.04 (0.87, 1.23)	1.06 (0.88, 1.27)	1.03 (0.85, 1.26)	1.11 (0.90, 1.37)	1.05 (0.89, 1.24)	1.06 (0.89, 1.27)	1.11 (0.92, 1.33)
Professionals in art and culture	1.54 (1.01, 2.36)	1.18 (0.84, 1.65)	1.27 (0.89, 1.80)	1.53 (1.04, 2.25)	1.18 (0.87, 1.60)	1.18 (0.92, 1.50)	1.24 (0.95, 1.60)	1.26 (0.96, 1.64)
Technical positions in art, culture, recreation and sport	0.89 (0.69, 1.15)	0.99 (0.82, 1.20)	0.99 (0.81, 1.22)	0.90 (0.72, 1.13)	1.05 (0.80, 1.39)	0.96 (0.77, 1.19)	0.94 (0.74, 1.18)	0.99 (0.77, 1.27)
**Sales and service**	1.05 (0.93, 1.18)	1.03 (0.93, 1.13)	1.05 (0.95, 1.16)	1.05 (0.94, 1.17)	1.03 (0.92, 1.15)	0.94 (0.86, 1.03)	0.93 (0.85, 1.02)	1.00 (0.91, 1.11)
Retail sales supervisors and specialized sales	1.11 (0.84, 1.46)	1.05 (0.84, 1.30)	1.12 (0.89, 1.40)	1.11 (0.86, 1.43)	1.03 (0.77, 1.38)	0.88 (0.69, 1.12)	0.95 (0.74, 1.22)	0.91 (0.70, 1.18)
Service supervisors and specialized service	0.85 (0.62, 1.18)	0.90 (0.70, 1.16)	0.98 (0.74, 1.28)	0.84 (0.63, 1.13)	0.99 (0.77, 1.27)	0.98 (0.80, 1.21)	0.98 (0.79, 1.22)	0.99 (0.80, 1.24)
Sales representatives and salespersons - wholesale and retail trade	1.10 (0.81, 1.49)	0.93 (0.73, 1.18)	0.98 (0.76, 1.26)	1.07 (0.81, 1.41)	0.92 (0.65, 1.30)	0.96 (0.73, 1.26)	0.88 (0.66, 1.17)	0.96 (0.71, 1.30)
Service representatives and other customer and personal services	0.87 (0.67, 1.13)	1.01 (0.81, 1.24)	1.01 (0.81, 1.26)	0.91 (0.71, 1.16)	1.11 (0.91, 1.35)	0.89 (0.76, 1.04)	0.85 (0.72, 1.01)	1.05 (0.88, 1.25)
Sales support	1.24 (0.84, 1.81)	1.24 (0.92, 1.68)	1.31 (0.95, 1.80)	1.32 (0.93, 1.86)	0.82 (0.62, 1.06)	0.72 (0.58, 0.90)	0.67 (0.53, 0.85)	0.73 (0.57, 0.92)
Service support and other service positions	1.13 (0.91, 1.40)	1.07 (0.91, 1.27)	1.02 (0.85, 1.22)	1.11 (0.92, 1.35)	1.16 (0.94, 1.43)	1.25 (1.05, 1.49)	1.30 (1.08, 1.56)	1.27 (1.05, 1.54)
**Trades, transport and equipment operators and related**	1.04 (0.93, 1.17)	1.05 (0.97, 1.15)	1.04 (0.95, 1.14)	1.06 (0.96, 1.17)	0.68 (0.46, 1.01)	1.04 (0.76, 1.43)	0.99 (0.71, 1.39)	0.78 (0.55, 1.10)
Industrial, electrical and construction trades	0.99 (0.85, 1.16)	1.11 (0.99, 1.25)	1.13 (1.00, 1.29)	1.03 (0.90, 1.19)	0.65 (0.29, 1.45)	0.77 (0.40, 1.48)	0.57 (0.29, 1.14)	0.72 (0.35, 1.46)
Maintenance and equipment operation trades	1.01 (0.82, 1.25)	0.91 (0.77, 1.07)	0.92 (0.77, 1.09)	0.98 (0.81, 1.18)	0.48 (0.20, 1.18)	1.44 (0.70, 2.96)	1.37 (0.63, 2.95)	0.66 (0.30, 1.46)
Other installers, repairers and servicers and material handlers	1.17 (0.73, 1.89)	1.30 (0.91, 1.85)	1.20 (0.82, 1.76)	1.32 (0.85, 2.03)	2.45 (0.69, 8.67)	1.04 (0.37, 2.88)	1.33 (0.45, 3.94)	1.94 (0.63, 5.92)
Transport and heavy equipment operation and related maintenance	1.05 (0.85, 1.30)	0.97 (0.82, 1.15)	0.97 (0.82, 1.16)	1.03 (0.85, 1.26)	0.65 (0.34, 1.22)	0.87 (0.52, 1.45)	1.01 (0.58, 1.75)	0.67 (0.38, 1.17)
Trades helpers, construction labourers and related	1.40 (0.92, 2.14)	1.27 (0.90, 1.77)	0.95 (0.67, 1.37)	1.46 (0.99, 2.14)	0.56 (0.16, 1.98)	2.42 (0.87, 6.73)	1.43 (0.48, 4.25)	1.00 (0.33, 3.08)
**Natural resources, agriculture and related production**	1.11 (0.86, 1.43)	1.12 (0.92, 1.37)	1.03 (0.83, 1.27)	1.09 (0.87, 1.37)	0.73 (0.45, 1.21)	1.46 (0.98, 2.19)	1.51 (0.98, 2.32)	0.90 (0.58, 1.40)
Supervisors and technical positions in natural resources, agriculture and related production	1.36 (0.91, 2.02)	1.09 (0.79, 1.49)	0.98 (0.71, 1.36)	1.29 (0.90, 1.85)	1.30 (0.37, 4.62)	3.19 (1.15, 8.84)	3.98 (1.34, 11.79)	2.10 (0.69, 6.42)
Workers in natural resources, agriculture and related production	1.43 (0.91, 2.27)	1.32 (0.92, 1.91)	1.21 (0.82, 1.79)	1.38 (0.91, 2.10)	0.65 (0.37, 1.15)	1.29 (0.82, 2.05)	1.34 (0.82, 2.18)	0.77 (0.47, 1.27)
Harvesting, landscaping and natural resources labourers	0.67 (0.43, 1.04)	0.99 (0.70, 1.40)	0.94 (0.65, 1.36)	0.71 (0.48, 1.06)	0.76 (0.13, 4.52)	1.03 (0.24, 4.36)	0.69 (0.15, 3.21)	0.77 (0.16, 3.73)
**Manufacturing and utilities**	1.09 (0.90, 1.33)	0.95 (0.81, 1.10)	0.93 (0.79, 1.10)	1.03 (0.86, 1.23)	0.87 (0.63, 1.20)	0.90 (0.68, 1.18)	0.87 (0.65, 1.15)	0.80 (0.59, 1.08)
Processing, manufacturing and utilities supervisors and central control operators	0.95 (0.68, 1.33)	0.80 (0.62, 1.05)	0.88 (0.67, 1.16)	0.89 (0.66, 1.21)	0.42 (0.19, 0.94)	0.85 (0.44, 1.62)	1.24 (0.62, 2.46)	0.49 (0.24, 0.98)
Processing and manufacturing machine operators and related production workers	0.94 (0.68, 1.30)	0.93 (0.73, 1.19)	0.80 (0.62, 1.04)	0.88 (0.66, 1.17)	0.83 (0.50, 1.36)	0.91 (0.60, 1.38)	0.86 (0.55, 1.35)	0.84 (0.53, 1.32)
Assemblers in manufacturing	1.21 (0.80, 1.84)	1.25 (0.91, 1.72)	1.33 (0.94, 1.88)	1.25 (0.87, 1.81)	1.35 (0.72, 2.55)	0.96 (0.56, 1.66)	0.83 (0.48, 1.44)	1.11 (0.61, 2.02)
Labourers in processing, manufacturing and utilities	2.35 (1.25, 4.42)	0.94 (0.58, 1.54)	0.92 (0.55, 1.56)	1.91 (1.09, 3.35)	0.99 (0.44, 2.22)	0.83 (0.40, 1.72)	0.67 (0.34, 1.34)	0.75 (0.34, 1.67)
**Unemployed**	1.00 (0.88, 1.14)	1.00 (0.90, 1.10)	1.01 (0.91, 1.13)	0.98 (0.88, 1.10)	1.03 (0.93, 1.13)	1.00 (0.92, 1.09)	0.99 (0.91, 1.08)	1.01 (0.92, 1.11)

*Abbreviations*: – no data, *CHMS* Canadian Health Measures Survey, *GMR* Geometric mean ratio, *CI* Confidence interval, *MEP* Mono-ethyl phthalate, *MnBP* Mono-n-butyl phthalate, *MEOHP* Mono-(2-ethyl-5-oxohexyl) phthalate, *MEHHP* Mono-(2-ethyl-5-hydroxyhexyl) phthalate, *MEHP* Mono-(2-ethylhexyl) phthalate, *MCPP* Mono-(3-carboxypropyl) phthalate, *MBzP* Mono-benzyl phthalate, *MMP* Mono-methyl phthalate, *MCHP* Mono-cyclohexyl phthalate, *MiNP* Mono-isononyl phthalate, *MOP* Mono-octyl phthalate, *LMWP* Low molecular weight phthalate, *HMWP* High molecular weight phthalate, *DEHP* Di(2-ethylhexyl) phthalate

^a^Based on geometric mean concentration units of nmol/g creatinine

∑LMWP = Molar sum of MMP + MEP + MnBP + MCHP

∑HMWP = Molar sum of MEOHP + MEHHP + MEHP + MiNP + MOP + MCPP + MBzP

∑DEHP = Molar sum of MEOHP + MEHHP + MEHP

∑Total = Molar Sum of MMP + MEP + MnBP + MCHP + MEOHP + MEHHP + MEHP + MiNP + MOP + MCPP + MBzP

^b^Least-squares GMRs computed by multivariable linear regression, using occupational terms as binary indicators, adjusting for: age (continuous), CHMS cycle, ethnicity, household income, smoking status, six dietary consumptions groups (meat and alternatives, dairy, grains, fruits and vegetables, seafood, bottled/canned beverages), drinking water type, and physical activity

In a sensitivity analysis of urinary dilution adjustment methods (Supplemental Table 10), SG-corrected GMRs were slightly attenuated relative to creatinine-corrected estimates, particularly for some high-exposure occupations, such as *assemblers in manufacturing* and *trades helpers*; however, the overall patterns of association remained consistent. Estimates for industry of employment largely corroborated occupational findings (Supplemental Table 11).

## Discussion

We identified multiple occupations with elevated urinary phthalate metabolite concentrations after adjustment for a range of sociodemographic, lifestyle, and dietary factors. Extending beyond the well-documented risks in plastics manufacturing, our analysis indicated modestly elevated exposures among workers in trades, transport, and equipment operation – particularly construction and industrial trades – and in natural resources, agriculture, and related production, groups previously underrepresented in the literature. Noteworthy elevations were also observed in specific health, service, manufacturing, and arts occupations, with marked sex-specific patterns. These findings indicate that occupational exposures, beyond established industrial settings, may contribute meaningfully to the phthalate body burden and highlight the critical role of population-based biomonitoring in identifying vulnerable worker subgroups. To our knowledge, this is one of the first studies to systematically investigate occupational determinants of phthalate exposure across a wide spectrum of jobs within a contemporary, general population sample in North America. At the same time, our analysis relied on a single spot urine sample, which may not reflect persistent exposures and is subject to day-to-day variability. This limitation should be considered as a potential source of measurement error when interpreting the observed associations.

Consistent with prior reports from biomonitoring studies [16, 18, 58, 59], including the CHMS [24, 29, 50], we found near-universal exposure to metabolites of DEHP, benzyl butyl phthalate (BzBP), diethyl phthalate (DEP), DBP, and di-n-octyl phthalate (DnOP). However, concentrations declined markedly from 2007 to 2019, especially for DEHP metabolites, coinciding with stricter regulations in Canada and elsewhere [60–62]. Notably, though, MiNP, a metabolite of the common DEHP substitute diisononyl phthalate (DiNP), showed attenuated reductions, particularly in Cycle 5 (2016–2017), possibly reflecting a relative shift toward DEHP substitutes, amid an overall decline in phthalate use. Over a comparable period, urinary phthalate concentrations were generally higher in NHANES than in the CHMS for most metabolites, particularly MEP and MnBP, while some HMWP metabolites like MEHHP and MEOHP show roughly comparable levels [63].

A notable finding of our investigation relates to the modestly elevated phthalate concentrations, across most metabolite measures, among workers in natural resources, agriculture, and related production. To our knowledge, this is the first North American study to identify this occupational group as potentially high-risk. Agricultural and forestry workers are known to encounter diverse contaminants [64], and a growing body of evidence from China [65, 66] and Europe [67, 68] indicates widespread phthalate contamination in agricultural soils, largely from plastic mulches and films. A recent pilot study in Western China found that > 60% of workers in agricultural plastic greenhouses faced significant phthalate exposure risks [69], with intake from plastic greenhouse-grown produce and inhalation in greenhouses constituting the primary exposure pathways. As such structures are commonly used to extend growing seasons in colder climates like Canada’s, these findings may have direct relevance to the exposures observed in our study population.

The elevated concentrations observed among workers in trades, transport, and equipment operation, particularly in construction and industrial trades, align with the known use of phthalate-laden materials in these sectors. Construction materials such as PVC piping, vinyl flooring, wiring cables, adhesives, sealants, and paints are well-documented sources of HMWPs like DEHP and DiNP [70–72]. Our findings of modestly increased MCPP (a metabolite of DBP and DnOP) and DEHP metabolites in these workers are highly consistent with this exposure profile. Prolonged time in vehicles among workers in transport, equipment operation, agriculture, and forestry may also contribute to exposure from synthetic materials used in vehicle interiors (e.g., floor mats, dashboards) [73, 74]. While direct biomonitoring evidence for phthalates in specific trades is limited, CAREX Canada has identified high occupational exposures to styrene, a plastic monomer that frequently co-occurs with phthalates in plastic products, among Canadian workers in automotive repair trades [75]. The pattern of higher exposure among males in these trades likely reflects both their greater representation in hands-on, material-intensive roles and potential differences in tasks assignments and safety practices.

In contrast to the well-documented elevations among plastics manufacturing workers [21], associations for manufacturing occupations were surprisingly modest, potentially reflecting aggregated job tasks and/or effective workplace controls. Nonetheless, we observed elevated concentrations in specific hands-on roles, such as assemblers and labourers in processing, consistent with higher exposure in production and material handling tasks [21].

Workers in service support and other service positions also demonstrated elevated concentrations, with increased and precise GMRs for several metabolites. These elevations may be, in part, attributable to cleaners, a prominent subgroup potentially exposed through phthalate-containing gloves and cleaning products, particularly those with fragrance formulations [76]. The higher GMRs observed for women (compared to men) in this group could reflect its gendered occupational distribution, as cleaners have historically been a predominantly female workforce. Another likely contributing subgroup are beauty salon attendants, who are known to encounter phthalate exposures in cosmetics and personal care products [21]. Though, our sample size precluded a granular analysis of these subgroups. Separately, professionals in art and culture exhibited increased concentrations, which may be associated with the handling of materials such as plastics, paints, and adhesives.

Demographic and lifestyle associations largely aligned with established literature: exposures were higher among females [16–18, 54], smokers [10, 18, 19], non-white ethnic groups [15, 16, 77], and those of lower socioeconomic status [10, 15, 16], but, contrary to some reports [78, 79], increased with age. Consumption of ultra-processed and high-fat foods (e.g., dairy, processed meats) is a known source of phthalate exposure [10, 80–82]. However, fruit and vegetable intake (including processed and canned items) showed the strongest and most consistent associations with higher phthalate concentrations, followed by bottled/canned beverage consumption. Similar patterns have been observed in NHANES [12, 83] and among older Chinese adults [18]. Although the pathways linking fruit and vegetable consumption to phthalate exposure remain poorly characterized, they may involve the water solubility of some phthalates [12], food processing and packaging methods [13], and uptake through agricultural pathways [65, 67, 84].

There exists some debate regarding optimal approaches for adjusting urinary [phthalate] biomarker concentrations for dilution, including whether creatinine (measured or predicted) should be included as a model covariate versus applying direct dilution standardization, with some biomonitoring studies favoring SG-standardization or adjustment when available [85]. In the present study, primary analyses used creatinine-standardized concentrations because urinary SG was only measured beginning in CHMS Cycle 2, whereas creatinine was available in all cycles included here (Cycles 1, 2, 5, and 6), thereby maximizing sample size (*n* = 3,072 vs. *n* = 4,259 participants) and statistical precision for stratified analyses across detailed occupational groups. Sensitivity analyses using SG-standardized concentrations produced similar results, with only minor attenuation of adjusted GMR estimates, supporting robustness to the dilution adjustment method. Creatinine standardization also aligns with most related phthalate studies [21] and facilitated consistent comparisons across cycles, given our objective of comparing relative exposure levels across occupations. We opted for direct standardization rather than covariate adjustment given that creatinine may be strongly associated with several predictors in our models (e.g., age, sex, ethnicity), and covariate adjustment can complicate interpretation when the exposure metric itself remains dilution-dependent.

This study has several limitations that should be considered. First, we assumed that a single spot urine measurement reflects an individual’s medium- to long-term phthalate exposure, which may introduce measurement error given the short biological half-lives of most phthalates [10]. Since samples were collected at scheduled MEC appointments, including weekdays and weekends, some samples may not have been collected after recent work activities and may not have captured occupational exposures. However, such non-differential measurement error would likely have introduced misclassification bias toward the null in our study. Second, for metabolites with low DFs, our substitution of values below the LOD may have led to overestimation of GMs in some groups that were in fact truly zero. However, these metabolites (i.e., MMP, MCHP, MiNP, MOP) contributed minimally to molar sum measures, and primary analyses focused on metabolites with DFs > 70%, reducing the potential impact of overestimation on our results. Third, despite the relatively large overall sample, small sample sizes within detailed occupational codes (three- and four-digit NOC levels) necessitated that analyses were limited to broader occupational groupings as to ensure adequate precision. This limitation also reduced the statistical power for subgroup analyses and increased the potential for unstable estimates within small groups. This limits the utility of the findings for prevention and intervention efforts, as such classifications may mask heterogeneity in exposures within finer occupational groups. Last, some non-occupational exposure sources, such as frequency of cosmetic and personal care product use (a known source of LMWPs), were not consistently available in the CHMS for adjustment, potentially leading to confounding.

Strengths of this study include the use of a national sample with high-quality biomonitoring data from a rigorous, well-established survey. The extensive measurement of urinary phthalate metabolites enabled the analysis of both individual and summary exposure metrics, including ‘newer’ compounds such as DiNP. The availability of both creatinine and specific gravity for dilution adjustment enhanced the robustness of our findings, permitting sensitivity analyses and facilitating comparison between correction approaches [33]. Furthermore, the comprehensive adjustment for a wide array of demographic, lifestyle, and dietary confounders strengthens the validity of the observed associations. Additional strengths include the standardized collection of biological specimens and near-uniform laboratory methods across CHMS cycles, rigorous laboratory quality assurance/control procedures that minimize analytical variability, and the multi-cycle structure of the survey, which provides stable exposure estimates across several years.

## Conclusion

Our findings indicate that occupational exposure to phthalates extends beyond traditional manufacturing settings to include sectors such as agriculture and natural resources, service, construction, and trades. This suggests that a broader range of workers may face potential health risks from these synthetic chemicals. Further research is warranted to better characterize these exposures and inform targeted protective measures for these occupational groups.

## Supplementary Information

Supplementary Material 1.

## Data Availability

The datasets analysed during the current study are not publicly available due to the regulations outlined in the Statistics Act of Canada, prohibiting the direct release of Canadian Health Measures Survey (CHMS) data and/or identification of the participating individuals. Requests for access can be directed to Statistics Canada through the Canadian Research Data Centre Network (CRDCN), [https://www.statcan.gc.ca/en/microdata/data-centres].

## Abbreviations

AIC: Akaike information criterion
BMI: Body mass index
BzBP: Benzyl butyl phthalate
CHMS: Canadian Health Measures Survey
CI: Confidence interval
DAG: Directed acyclic graph
DBP: Di-n-butyl phthalate
DEHP: Di(2-ethylhexyl) phthalate
DEP: Diethyl phthalate
DF: Detection frequency
DiNP: Diisononyl phthalate
DnOP: Di-n-octyl phthalate
FDR: False discovery rate
GM: Geometric mean
GMR: Geometric mean ratio
HMWP: High molecular weight phthalate
LMWP: Low molecular weight phthalate
LOD: Limit of detection
LODc: Highest observed limit of detection
MBzP: Monobenzyl phthalate
MCHP: Mono-cyclohexyl phthalate
MCPP: Mono(3-carboxypropyl) phthalate
MEC: Mobile examination center
MEHHP: Mono-(2-ethyl-5-hydroxyhexyl) phthalate
MEHP: Mono(2-ethylhexyl) phthalate
MEOHP: Mono(2-ethyl-5-oxohexyl) phthalate
MEP: Monoethyl phthalate
MiNP: Mono-isononyl phthalate
MMP: Monomethyl phthalate
MnBP: Mono-n-butyl phthalate
MOP: Mono-octyl phthalate
NAICS: North American Industry Classification System
NOC: National Occupational Classification
OR: Odds ratio
PVC: Polyvinyl chloride
SG: Specific gravity
∑DEHP: Sum of DEHP-devolving metabolites
∑HMWP: Sum of HMWPs
∑LMWP: Sum of LMWPs
∑Total phthalates: Sum of total phthalates
