# Supplementary material for: Occupation and urinary phthalate metabolite concentrations in a national survey of adults in Canada
Source: Environ Health. 2026 Apr 24;25:50. doi: 10.1186/s12940-026-01297-5 (PMC13248431; doi:10.1186/s12940-026-01297-5)
Supplement: Supplementary file 1 — Supplementary Material 1. [file 12940_2026_1297_MOESM1_ESM.docx]

**Occupation and Urinary Phthalate Metabolite Concentrations in a National Survey of Adults in Canada**

*** SUPPLEMENTAL MATERIAL ***

Patrick Hinton^1,2^, Ryann E. Yeo^2^, Joanne Kim^3^, Daniel R.S. Middleton^4^, Katherine Pullella^5^, Victoria Arrandale^1^, Nathan L. DeBono^1,2^

**Affiliations:**

1. Dalla Lana School of Public Health, University of Toronto, Toronto, Canada.
2. Occupational Cancer Research Centre, Ontario Health, Toronto, Canada.
3. Environment and Lifestyle Epidemiology Branch, International Agency for Research on Cancer, WHO, Lyon, France.
4. Centre for Public Health, School of Medicine, Dentistry and Biomedical Sciences, Queen’s University Belfast, Belfast, UK.
5. Department of Nutritional Sciences, Temerty Faculty of Medicine, University of Toronto, Toronto, Ontario, Canada.

**Corresponding Author:**

Patrick Hinton

[patrick.hinton@queensu.ca](mailto:patrick.hinton@queensu.ca)

**Supplemental Figure 1.** Selection of the analytic sample from the CHMS; Cycles 1, 2, 5, and 6 (2007-2019).

Abbreviations: CHMS, Canadian Health Measures Survey; MEC; Mobile Examination Centre.

*Detailed sample sizes for listed inclusions not reportable due to Statistics Canada Research Data Centre requirements.

**Supplemental Table 1.** Molecular weight classification of eleven measured phthalate metabolites across CHMS Cycles 1, 2, 5, and 6.

| **Parent phthalates** | **Abbreviations** | **Urinary metabolites** | **Abbreviations** | **Molecular Weight (g/mol)** |
| --- | --- | --- | --- | --- |
|  |  |  |  |  |
| **Low Molecular Weight Phthalates^a^** | **LMWPs** |  |  |  |
| Dimethyl phthalate | DMP | Mono-methyl phthalate | MMP | 194.19 |
| Diethyl phthalate | DEP | Mono-ethyl phthalate | MEP | 222.12 |
| Di-n-butyl phthalate | DBP | Mono-n-butyl phthalate | MnBP | 222.28 |
| Dicyclohexyl phthalate | DCHP | Mono-cyclohexyl phthalate | MCHP | 248.27 |
|  |  |  |  |  |
| **High Molecular Weight Phthalates^a^** | **HMWPs** |  |  |  |
| Di(2-ethylhexyl) phthalate | **DEHP** |  |  |  |
|  |  | Mono-(2-ethyl-5-oxohexyl) phthalate | MEOHP | 306.34 |
|  |  | Mono-(2-ethyl-5-hydroxyhexyl) phthalate | MEHHP | 308.35 |
|  |  | Mono-(2-ethylhexyl) phthalate | MEHP | 278.34 |
|  |  |  |  |  |
| Diisononyl phthalate | DiNP | Mono-isononyl phthalate | MiNP | 312-320^b^ |
|  |  |  |  |  |
| Di-n-octyl phthalate | DnOP | Mono-octyl phthalate | MOP | 278.34 |
|  |  | Mono-(3-carboxypropyl) phthalate | MCPP | 252.23 |
|  |  |  |  |  |
| Benzyl butyl phthalate | BzBP | Mono-benzyl phthalate | MBzP | 256.26 |
|  |  |  |  |  |

Abbreviations: CHMS, Canadian Health Measures Survey.

Adapted from Liu et al., 2022 (1).

^a^High molecular weight phthalates (HMWPs) include those with molecular weight ≥250 g/mol and low molecular weight phthalates (LMWPs) are those with molecular weight <250 g/mol.

^b^Varies due to mixed isomers.

**Supplemental Table 2**. Cycle-specific analytical LODs for eleven measured phthalate metabolites in CHMS Cycles 1, 2, 5, and 6.

|  | **LOD (µg/L)** | | | |
| --- | --- | --- | --- | --- |
| **Phthalate Metabolite** | **Cycle 1** | **Cycle 2** | **Cycle 5** | **Cycle 6** |
| Mono-methyl phthalate (MMP) | 5.00 | 5.00 | 0.21 | 0.21 |
| Mono-ethyl phthalate (MEP) | 0.50 | 0.30 | 0.98 | 0.76 |
| Mono-(3-carboxypropyl) phthalate (MCPP) | 0.20 | 0.06 | 0.14 | 0.14 |
| Mono-n-butyl phthalate (MnBP) | 0.20 | 0.20 | 0.60 | 0.60 |
| Mono-cyclohexyl phthalate (MCHP) | 0.20 | 0.09 | 0.25 | 0.25 |
| Mono-benzyl phthalate (MBzP) | 0.20 | 0.05 | 0.37 | 0.14 |
| Mono-(2-ethylhexyl) phthalate (MEHP) | 0.20 | 0.08 | 0.11 | 0.08 |
| Mono-(2-ethyl-5-oxohexyl) phthalate (MEOHP) | 0.20 | 0.10 | 0.17 | 0.17 |
| Mono-(2-ethyl-5-hydroxyhexyl) phthalate (MEHHP) | 0.40 | 0.40 | 0.22 | 0.22 |
| Mono-octyl phthalate (MOP) | 0.70 | 0.30 | 0.16 | 0.16 |
| Mono-isononyl phthalate (MiNP) | 0.40 | 0.30 | 0.37 | 0.15 |

Abbreviations: CHMS, Canadian Health Measures Survey; LOD, Limit of detection.

For linear models: Metabolite concentrations <LOD are imputed by $LOD/\surd2$ in a metabolite and cycle-specific manner.

For logistic models: Metabolite concentrations <LOD_c_ (the highest observed LOD across cycles for a given metabolite) imputed by $LODc/\surd2$.

**
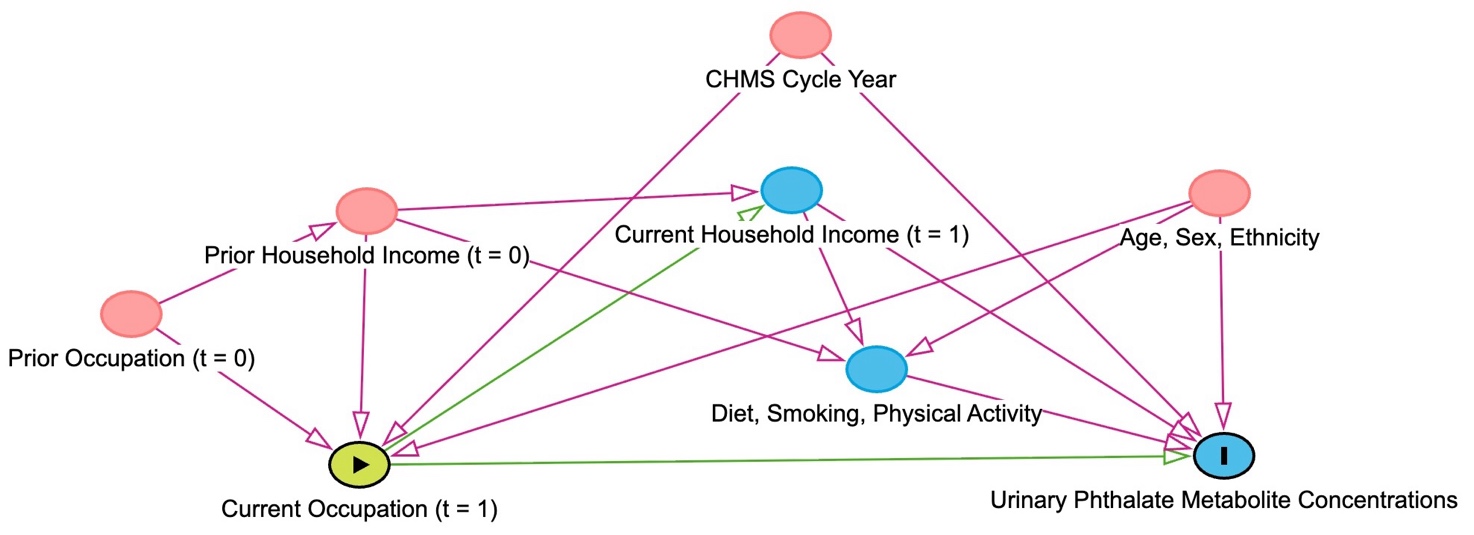
Supplemental Figure 2.** Directed acyclic graph illustrating the relationship between occupation (exposure) and urinary phthalate metabolite concentrations (outcome) in the CHMS.

Abbreviations: CHMS, Canadian Health Measures Survey.

**Supplemental Table 3**. CHMS Cycle 1 food groups and intake questions, with changes introduced in Cycles 2, 5, and 6.

| Food Groups | Dietary Components of Past-year Intake Assessed via Food Frequency Questionnaire (FFQ) | Cycle & Corresponding Additions / Modifications | |
| --- | --- | --- | --- |
|  |  | Cycle | Addition/Modification |
| Meat (& Alternatives) | Red Meat  Liver (including all types of liver such as beef, veal, port or chicken, but excluding liverwurst and liver pâté)  Organ meats such as kidneys, heart or giblets  Beef or pork hotdogs  Sausage or bacon (including all types of sausages such as breakfast, pepperoni and Kielbasa but excluding low-fat, light or turkey varieties)  Eggs/egg dishes including the yolk (excluding all egg dishes made with only egg whites) Egg dishes could include such things as eggs, omelets, frittata or quiche. | 5,6 | Eggs omega-3 enriched |
| Dairy Products | Milk or enriched milk substitutes or use them on cereal  Cottage cheese  Yogurt, excluding frozen yogurt  Ice cream or frozen yogurt | 5,6 | Processed cheese  Other types of cheese |
| Grain & Rice Products | Hot or cold cereal  Brown bread, including bagels, rolls, pita bread or tortillas  White bread, including bagels, rolls, pita bread or tortillas  Any kind of pasta (including spaghetti, noodles, macaroni & cheese or pasta salad)  Any kind of rice | 5,6  2,5,6 | Whole grain bread  *Rice altered to specify:*  Instant, seasoned, brown, white or wild rice |
| Bottled & Canned Drinks | Regular soft drinks  Diet soft drinks  Sports drinks  Fruit juices  Fruit flavored drinks  Vegetable juice | 5,6 | *Fruit juice altered to specify:*  Orange or grapefruit juice  Other juice |
| Fruit & Vegetables | Tomatoes or tomato sauce, including salsa, tomato soup and spaghetti sauce but excluding tomato paste, ketchup or pizza sauce  Lettuce or green leafy salad with or without other vegetables  Spinach, mustard greens or collards excluding kale  Other potatoes including baked, boiled, mashed or in potato salad, but excluding sweet potatoes  All other types of vegetables, excluding those already mentioned  Fruit (fresh, frozen, or canned) | 2,5,6 | *Fruit altered to specify:*  Citrus Fruits  Strawberries (in peak season and out of season)  Other types of fruits |
| Seafood Products | Saltwater fish such as salmon, tuna or fish sticks  Fresh water fish such as trout, walleye or pickerel.  Shellfish, such as shrimp, mussels, scallops, lobster, clams, oysters or crab | 2  5,6 | *Updated to only include:*  Atlantic Cod, Trout, Rainbow Trout, Lake Trout, Pike, Jackfish, Pickerel, Walleye, Halibut, Tilefish, Sablefish, Black Cod, Bass [Sea, Small, Large Mouth], Grouper, Escolar, Orange Roughy, Swordfish, Marlin, Shark, Fresh Or Frozen Salmon, Smoked Salmon, Canned Or Pouched Salmon, Tuna Steak/Fillet, Canned Tuna , Fish Sticks Or General Fresh Or Salt Water Fish,  Calamari or Squid, Oysters, Scallops, Mussels, Shrimp, Lobster, Surimi or Imitation Crab, Other shellfish  *Updated to only include:*  Tuna (can or pouch, steak or fillets), Salmon (can or pouch, fresh, frozen or smoked), Shark, Marlin, Swordfish, Mackerel, Herring, Other Fish, Calamari or Squid, Oysters, Scallops, Mussels, Shrimp, Lobster, Surimi or Imitation Crab, Other shellfish |

Abbreviations: CHMS, Canadian Health Measures Survey; FFQ, Food frequency questionnaire.

Adapted from Pullella, 2024 (2) and Hosseini et al., 2019 (3).

**Supplemental Table 4**. Number of workers across broad- and major-level occupations, overall (n = 4,259) and by sex (n_male_ = 2,135, n_female_ = 2,124), CHMS, 2007-2019.

| **Occupation**  **(Broad = Bold; Major = Unbolded)** | **Number of Workers** | | |
| --- | --- | --- | --- |
|  | **Overall (n = 4,259)** | **Male (n = 2,135)** | **Female (n = 2,124)** |
| **Management** | 308 | 192 | 116 |
| Senior management | - | - | - |
| Specialized middle management | - | - | - |
| Middle management in retail and wholesale trade and customer services | - | - | - |
| Middle management in trades, transportation, production and utilities | - | - | - |
| **Business, finance and administration** | 555 | 189 | 366 |
| Professionals in business and finance | 169 | 90 | 79 |
| Administrative and financial supervisors and administrative positions | 187 | 42 | 145 |
| Finance, insurance and related business administrative positions | 41 | - | - |
| Office support | 101 | - | - |
| Distribution, tracking and scheduling co-ordination | 57 | 36 | 21 |
| **Natural and applied sciences and related** | 300 | 228 | 72 |
| Professionals in natural and applied sciences | 195 | 152 | 43 |
| Technical positions related to natural and applied sciences | 105 | 76 | 29 |
| **Health** | 247 | 54 | 193 |
| Professionals in nursing | 64 | - | - |
| Professionals in health (except nursing) | 68 | - | - |
| Technical positions in health | 62 | - | - |
| Assisting in support of health services | 53 | - | - |
| **Education, law and social, community and government services** | 381 | 128 | 253 |
| Professionals in education services | 166 | 57 | 109 |
| Professionals in law and social, community and government services | 86 | 34 | 52 |
| Paraprofessionals in legal, social, community and education services | 67 | - | - |
| Front-line public protection services | 25 | - | - |
| Care providers and educational, legal and public protection support | 37 | - | - |
| **Art, culture, recreation and sport** | 161 | 80 | 81 |
| Professionals in art and culture | 56 | 20 | 36 |
| Technical positions in art, culture, recreation and sport | 105 | 60 | 45 |
| **Sales and service** | 622 | 286 | 336 |
| Retail sales supervisors and specialized sales | 88 | 50 | 38 |
| Service supervisors and specialized service | 88 | 34 | 54 |
| Sales representatives and salespersons - wholesale and retail trade | 70 | 41 | 29 |
| Service representatives and other customer and personal services | 142 | 53 | 89 |
| Sales support | 74 | 25 | 49 |
| Service support and other service positions | 160 | 83 | 77 |
| **Trades, transport and equipment operators and related** | 400 | 378 | 22 |
| Industrial, electrical and construction trades | 172 | - | - |
| Maintenance and equipment operation trades | 92 | - | - |
| Other installers, repairers and servicers and material handlers | 20 | - | - |
| Transport and heavy equipment operation and related maintenance | 94 | - | - |
| Trades helpers, construction labourers and related | 22 | - | - |
| **Natural resources, agriculture and related production** | 72 | - | - |
| Supervisors and technical positions in natural resources, agriculture and related production | 26 | - | - |
| Workers in natural resources, agriculture and related production | 27 | - | - |
| Harvesting, landscaping and natural resources labourers | 19 | - | - |
| **Manufacturing and utilities** | 131 | 100 | 31 |
| Processing, manufacturing and utilities supervisors and central control operators | - | - | - |
| Processing and manufacturing machine operators and related production workers | - | - | - |
| Assemblers in manufacturing | - | - | - |
| Labourers in processing, manufacturing and utilities | - | - | - |
| **Unemployed** | 1082 | 441 | 641 |

Abbreviations: CHMS, Canadian Health Measures Survey.

Note: Due to Statistics Canada Research Data Centre requirements, sample sizes for some small occupational groups are not presented (denoted by ‘-’).

**Supplemental Table 5**. Number of workers across sector-level industry, overall (n = 4,259) and by sex (n_male_ = 2,135, n_female_ = 2,124), CHMS, 2007-2019.

| **Industry of Employment**  **(Sector-level)** | **Number of Workers** | | |
| --- | --- | --- | --- |
|  | **Overall (n = 4,259)** | **Male (n = 2,135)** | **Female (n = 2,124)** |
| Agriculture, forestry, fishing and hunting | 62 | - | - |
| Mining, quarrying, and oil and gas extraction | 42 | - | - |
| Utilities | 26 | - | - |
| Construction | 221 | 193 | 28 |
| Manufacturing | 310 | 244 | 66 |
| Wholesale trade | 117 | 86 | 31 |
| Retail trade | 272 | 119 | 153 |
| Transportation and warehousing | 159 | 107 | 52 |
| Information and cultural industries | 77 | 49 | 28 |
| Finance and insurance | 152 | 81 | 71 |
| Real estate and rental and leasing | 50 | 29 | 21 |
| Professional, scientific and technical services | 292 | 168 | 124 |
| Administrative and support, waste management and remediation services | 146 | 63 | 83 |
| Educational services | 249 | 85 | 164 |
| Health care and social assistance | 399 | 85 | 314 |
| Arts, entertainment and recreation | 82 | 45 | 37 |
| Accommodation and food services | 184 | 84 | 100 |
| Other services (except public administration) | 134 | 54 | 80 |
| Public administration | 203 | 102 | 101 |
| Unemployed | 1082 | 441 | 641 |

Abbreviations: CHMS, Canadian Health Measures Survey.

Note: Due to Statistics Canada Research Data Centre requirements, sample sizes for some small industry groups are not presented (denoted by ‘-’).

**Supplemental Figure 3.** Spearman correlation matrix of urinary phthalate metabolite concentrations (DF ≥70%), uncorrected (top) and creatinine-corrected (bottom), analytic CHMS sample (n = 4,259), 2007-2019.

Abbreviations: CHMS, Canadian Health Measures Survey; DF, Detection frequency; MMP, Mono-methyl phthalate; MEP, Mono-ethyl phthalate; MnBP, Mono-n-butyl phthalate; MCHP, Mono-cyclohexyl phthalate; MEOHP, Mono-(2-ethyl-5-oxohexyl) phthalate; MEHHP, Mono-(2-ethyl-5-hydroxyhexyl) phthalate; MEHP, Mono-(2-ethylhexyl) phthalate; MiNP, Mono-isononyl phthalate; MOP, Mono-octyl phthalate; MCPP, Mono-(3-carboxypropyl) phthalate; MBzP, Mono-benzyl phthalate.

**Supplemental Table 6.** Association between occupations with creatinine-corrected concentrations of phthalate metabolites (DF ≥70%) and summary groups, CHMS (n = 4,259), 2007-2019.

| **Occupation**  **(Broad = Bold; Major = Unbolded)** | **GMR**^c^ **(95% CI)** | | | | | | | | | | |
| --- | --- | --- | --- | --- | --- | --- | --- | --- | --- | --- | --- |
|  | **MEP**^a^ | **MnBP**^a^ | **MEOHP**^a^ | **MEHHP**^a^ | **MEHP**^a^ | **MCPP**^a^ | **MBzP**^a^ | **∑LMWP**^b^ | **∑HMWP^b^** | **∑DEHP**^b^ | **∑Total**^b^ |
| **Management** | 0.98 (0.85, 1.14) | 0.89 (0.82, 0.97) | 1.08 (0.98, 1.19) | 1.09 (1.00, 1.20) | 1.04 (0.93, 1.16) | 1.00 (0.90, 1.12) | 0.93 (0.83, 1.04) | 0.94 (0.84, 1.05) | 1.03 (0.95, 1.13) | 1.09 (0.99, 1.19) | 0.97 (0.88, 1.07) |
| Senior management | 1.54 (0.58, 4.08) | 0.91 (0.49, 1.68) | 0.81 (0.43, 1.52) | 0.78 (0.41, 1.48) | 0.78 (0.34, 1.76) | 0.74 (0.35, 1.58) | 0.91 (0.42, 1.99) | 1.23 (0.54, 2.81) | 0.80 (0.42, 1.53) | 0.80 (0.40, 1.59) | 1.26 (0.56, 2.84) |
| Specialized middle management | 1.08 (0.87, 1.34) | 0.96 (0.85, 1.09) | 1.20 (1.04, 1.38) | 1.25 (1.08, 1.44) | 1.26 (1.06, 1.49) | 1.10 (0.93, 1.30) | 0.90 (0.76, 1.07) | 1.06 (0.89, 1.26) | 1.13 (0.99, 1.30) | 1.24 (1.07, 1.43) | 1.12 (0.96, 1.30) |
| Middle management in retail and wholesale trade and customer services | 0.97 (0.75, 1.25) | 0.85 (0.74, 0.99) | 1.06 (0.90, 1.25) | 1.07 (0.90, 1.26) | 0.98 (0.81, 1.19) | 0.96 (0.78, 1.17) | 0.94 (0.77, 1.15) | 0.89 (0.73, 1.08) | 1.01 (0.86, 1.18) | 1.06 (0.90, 1.24) | 0.90 (0.75, 1.08) |
| Middle management in trades, transportation, production and utilities | 0.85 (0.66, 1.11) | 0.86 (0.74, 1.00) | 0.96 (0.81, 1.13) | 0.94 (0.79, 1.11) | 0.85 (0.70, 1.05) | 0.94 (0.77, 1.15) | 0.97 (0.79, 1.20) | 0.84 (0.68, 1.03) | 0.94 (0.80, 1.10) | 0.93 (0.78, 1.10) | 0.85 (0.70, 1.02) |
| **Business, finance and administration** | 1.09 (0.97, 1.21) | 0.94 (0.89, 1.01) | 0.99 (0.92, 1.07) | 0.98 (0.91, 1.06) | 0.94 (0.87, 1.03) | 0.99 (0.90, 1.07) | 0.84 (0.77, 0.92) | 1.04 (0.95, 1.13) | 0.94 (0.88, 1.01) | 0.98 (0.91, 1.05) | 1.03 (0.95, 1.11) |
| Professionals in business and finance | 0.89 (0.74, 1.08) | 0.97 (0.87, 1.08) | 0.93 (0.82, 1.05) | 0.91 (0.80, 1.03) | 0.89 (0.77, 1.03) | 0.95 (0.82, 1.10) | 0.77 (0.66, 0.90) | 0.93 (0.80, 1.08) | 0.88 (0.78, 0.98) | 0.91 (0.81, 1.03) | 0.94 (0.82, 1.07) |
| Administrative and financial supervisors and administrative positions | 1.28 (1.07, 1.54) | 0.94 (0.84, 1.04) | 0.99 (0.88, 1.12) | 0.98 (0.87, 1.11) | 0.94 (0.82, 1.08) | 1.03 (0.89, 1.18) | 0.84 (0.73, 0.97) | 1.18 (1.03, 1.36) | 0.94 (0.84, 1.05) | 0.98 (0.87, 1.10) | 1.13 (1.00, 1.28) |
| Finance, insurance and related business administrative positions | 0.83 (0.57, 1.21) | 0.98 (0.79, 1.22) | 0.93 (0.73, 1.19) | 0.93 (0.73, 1.19) | 0.95 (0.71, 1.26) | 0.80 (0.60, 1.08) | 1.31 (0.97, 1.77) | 0.83 (0.62, 1.12) | 1.00 (0.80, 1.27) | 0.93 (0.73, 1.18) | 0.86 (0.66, 1.13) |
| Office support | 1.10 (0.86, 1.40) | 0.98 (0.86, 1.13) | 1.15 (0.98, 1.34) | 1.12 (0.96, 1.32) | 1.04 (0.87, 1.26) | 1.16 (0.96, 1.40) | 0.92 (0.76, 1.11) | 1.03 (0.85, 1.24) | 1.11 (0.95, 1.29) | 1.14 (0.97, 1.34) | 1.06 (0.89, 1.26) |
| Distribution, tracking and scheduling co-ordination | 1.20 (0.87, 1.65) | 0.88 (0.73, 1.05) | 0.99 (0.80, 1.21) | 1.03 (0.83, 1.27) | 1.01 (0.79, 1.29) | 0.84 (0.66, 1.07) | 0.82 (0.64, 1.06) | 1.07 (0.84, 1.37) | 0.89 (0.74, 1.09) | 1.01 (0.82, 1.24) | 1.03 (0.82, 1.28) |
| **Natural and applied sciences and related** | 0.85 (0.73, 0.98) | 0.94 (0.87, 1.03) | 0.97 (0.88, 1.07) | 0.99 (0.90, 1.08) | 1.01 (0.90, 1.13) | 0.89 (0.79, 0.99) | 0.98 (0.87, 1.10) | 0.91 (0.81, 1.02) | 0.99 (0.90, 1.08) | 0.98 (0.89, 1.08) | 0.94 (0.85, 1.04) |
| Professionals in natural and applied sciences | 0.82 (0.69, 0.98) | 0.87 (0.79, 0.97) | 0.95 (0.84, 1.07) | 0.96 (0.86, 1.08) | 0.97 (0.84, 1.11) | 0.85 (0.74, 0.98) | 0.93 (0.81, 1.07) | 0.86 (0.75, 0.99) | 0.96 (0.86, 1.07) | 0.96 (0.85, 1.08) | 0.89 (0.79, 1.01) |
| Technical positions related to natural and applied sciences | 0.92 (0.73, 1.16) | 1.09 (0.95, 1.24) | 1.02 (0.87, 1.19) | 1.03 (0.88, 1.20) | 1.08 (0.90, 1.30) | 0.97 (0.81, 1.16) | 1.08 (0.89, 1.30) | 1.01 (0.84, 1.21) | 1.04 (0.90, 1.21) | 1.03 (0.88, 1.20) | 1.04 (0.88, 1.23) |
| **Health** | 1.11 (0.94, 1.30) | 0.90 (0.82, 0.99) | 0.98 (0.88, 1.09) | 0.96 (0.86, 1.06) | 0.99 (0.88, 1.12) | 0.99 (0.88, 1.13) | 1.26 (1.11, 1.43) | 1.01 (0.89, 1.14) | 1.02 (0.92, 1.13) | 0.96 (0.87, 1.07) | 1.00 (0.89, 1.12) |
| Professionals in nursing | 1.25 (0.93, 1.70) | 0.95 (0.80, 1.14) | 1.05 (0.86, 1.27) | 1.02 (0.84, 1.25) | 1.08 (0.85, 1.38) | 1.07 (0.85, 1.36) | 1.34 (1.05, 1.71) | 1.14 (0.90, 1.44) | 1.11 (0.91, 1.34) | 1.03 (0.84, 1.26) | 1.13 (0.91, 1.40) |
| Professionals in health (except nursing) | 1.02 (0.76, 1.38) | 0.98 (0.82, 1.16) | 1.09 (0.90, 1.32) | 1.05 (0.87, 1.28) | 1.16 (0.93, 1.46) | 0.92 (0.73, 1.15) | 1.37 (1.08, 1.73) | 1.00 (0.79, 1.25) | 1.10 (0.91, 1.32) | 1.07 (0.89, 1.30) | 1.00 (0.81, 1.24) |
| Technical positions in health | 0.96 (0.70, 1.30) | 0.81 (0.68, 0.97) | 0.77 (0.63, 0.94) | 0.75 (0.61, 0.91) | 0.74 (0.59, 0.94) | 0.91 (0.71, 1.15) | 1.02 (0.80, 1.31) | 0.85 (0.67, 1.08) | 0.79 (0.66, 0.96) | 0.75 (0.61, 0.92) | 0.80 (0.65, 0.99) |
| Assisting in support of health services | 1.19 (0.86, 1.66) | 0.89 (0.74, 1.08) | 1.07 (0.86, 1.33) | 1.05 (0.85, 1.31) | 1.03 (0.80, 1.33) | 1.12 (0.87, 1.45) | 1.23 (0.94, 1.61) | 1.08 (0.83, 1.39) | 1.13 (0.92, 1.38) | 1.05 (0.85, 1.30) | 1.13 (0.90, 1.42) |
| **Education, law and social, community and government services** | 0.87 (0.76, 0.99) | 1.09 (1.01, 1.17) | 0.95 (0.88, 1.04) | 0.96 (0.88, 1.05) | 0.99 (0.90, 1.10) | 1.04 (0.94, 1.16) | 1.11 (1.00, 1.24) | 0.93 (0.84, 1.03) | 1.00 (0.92, 1.09) | 0.96 (0.89, 1.05) | 0.93 (0.85, 1.02) |
| Professionals in education services | 0.81 (0.67, 0.98) | 1.16 (1.04, 1.30) | 1.02 (0.90, 1.16) | 1.02 (0.90, 1.16) | 1.09 (0.94, 1.27) | 1.00 (0.86, 1.16) | 1.20 (1.03, 1.40) | 0.92 (0.79, 1.06) | 1.06 (0.94, 1.20) | 1.04 (0.92, 1.18) | 0.93 (0.81, 1.07) |
| Professionals in law and social, community and government services | 1.07 (0.82, 1.39) | 0.99 (0.85, 1.15) | 0.96 (0.81, 1.14) | 0.99 (0.83, 1.18) | 1.00 (0.82, 1.22) | 1.06 (0.86, 1.30) | 0.92 (0.75, 1.14) | 1.01 (0.83, 1.24) | 0.96 (0.82, 1.14) | 0.96 (0.81, 1.14) | 1.00 (0.83, 1.21) |
| Paraprofessionals in legal, social, community and education services | 0.78 (0.58, 1.05) | 1.05 (0.88, 1.24) | 1.02 (0.84, 1.23) | 1.05 (0.86, 1.27) | 1.02 (0.81, 1.29) | 1.10 (0.87, 1.38) | 1.28 (1.01, 1.63) | 0.87 (0.69, 1.10) | 1.11 (0.92, 1.34) | 1.04 (0.86, 1.27) | 0.91 (0.74, 1.12) |
| Front-line public protection services | 0.66 (0.41, 1.07) | 0.95 (0.72, 1.26) | 0.77 (0.56, 1.05) | 0.74 (0.54, 1.01) | 0.79 (0.54, 1.14) | 0.97 (0.66, 1.41) | 0.89 (0.61, 1.31) | 0.74 (0.51, 1.07) | 0.82 (0.61, 1.12) | 0.77 (0.56, 1.06) | 0.71 (0.51, 1.00) |
| Care providers and educational, legal and public protection support | 1.19 (0.80, 1.77) | 1.09 (0.87, 1.37) | 0.75 (0.58, 0.97) | 0.75 (0.58, 0.97) | 0.73 (0.54, 0.99) | 1.12 (0.82, 1.51) | 1.01 (0.74, 1.38) | 1.17 (0.86, 1.58) | 0.82 (0.65, 1.05) | 0.74 (0.57, 0.96) | 1.07 (0.81, 1.40) |
| **Art, culture, recreation and sport** | 1.02 (0.84, 1.23) | 1.07 (0.96, 1.19) | 1.05 (0.93, 1.19) | 1.06 (0.94, 1.21) | 1.09 (0.94, 1.26) | 1.08 (0.93, 1.25) | 0.98 (0.84, 1.14) | 1.06 (0.91, 1.24) | 1.04 (0.93, 1.17) | 1.06 (0.94, 1.20) | 1.07 (0.93, 1.22) |
| Professionals in art and culture | 1.18 (0.86, 1.63) | 1.20 (1.00, 1.45) | 1.25 (1.01, 1.54) | 1.22 (0.98, 1.50) | 1.29 (1.01, 1.65) | 1.13 (0.88, 1.45) | 0.97 (0.75, 1.25) | 1.26 (0.98, 1.62) | 1.16 (0.95, 1.41) | 1.23 (1.00, 1.51) | 1.31 (1.05, 1.64) |
| Technical positions in art, culture, recreation and sport | 0.94 (0.74, 1.19) | 1.00 (0.87, 1.14) | 0.96 (0.82, 1.12) | 0.99 (0.84, 1.15) | 0.99 (0.83, 1.19) | 1.05 (0.87, 1.26) | 0.99 (0.82, 1.19) | 0.96 (0.80, 1.16) | 0.98 (0.85, 1.14) | 0.98 (0.84, 1.14) | 0.95 (0.80, 1.12) |
| **Sales and service** | 1.08 (0.97, 1.20) | 0.99 (0.93, 1.05) | 0.97 (0.91, 1.04) | 0.99 (0.92, 1.06) | 0.97 (0.90, 1.06) | 0.97 (0.89, 1.05) | 1.00 (0.92, 1.09) | 1.03 (0.95, 1.12) | 0.98 (0.92, 1.05) | 0.98 (0.92, 1.05) | 1.02 (0.95, 1.10) |
| Retail sales supervisors and specialized sales | 1.10 (0.85, 1.43) | 0.98 (0.84, 1.13) | 0.99 (0.84, 1.18) | 1.02 (0.86, 1.21) | 1.12 (0.92, 1.37) | 1.00 (0.82, 1.22) | 0.83 (0.67, 1.02) | 1.06 (0.87, 1.30) | 0.96 (0.82, 1.13) | 1.03 (0.87, 1.22) | 1.01 (0.84, 1.21) |
| Service supervisors and specialized service | 1.01 (0.78, 1.31) | 0.93 (0.80, 1.07) | 0.99 (0.84, 1.17) | 1.00 (0.84, 1.18) | 0.93 (0.76, 1.14) | 0.96 (0.79, 1.17) | 0.84 (0.69, 1.04) | 0.94 (0.77, 1.15) | 0.95 (0.81, 1.12) | 0.98 (0.83, 1.16) | 0.93 (0.78, 1.12) |
| Sales representatives and salespersons - wholesale and retail trade | 1.10 (0.82, 1.46) | 0.90 (0.77, 1.06) | 0.93 (0.77, 1.12) | 0.93 (0.77, 1.12) | 0.94 (0.75, 1.17) | 0.80 (0.64, 1.00) | 1.00 (0.79, 1.26) | 0.99 (0.79, 1.25) | 0.94 (0.79, 1.13) | 0.93 (0.77, 1.12) | 1.00 (0.82, 1.22) |
| Service representatives and other customer and personal services | 1.10 (0.89, 1.34) | 0.92 (0.82, 1.03) | 0.90 (0.79, 1.03) | 0.91 (0.79, 1.04) | 0.88 (0.75, 1.03) | 0.98 (0.84, 1.15) | 1.04 (0.88, 1.22) | 1.00 (0.85, 1.17) | 0.93 (0.82, 1.06) | 0.90 (0.79, 1.03) | 0.98 (0.85, 1.14) |
| Sales support | 0.93 (0.70, 1.24) | 1.00 (0.85, 1.18) | 0.86 (0.72, 1.04) | 0.88 (0.73, 1.06) | 0.77 (0.62, 0.96) | 0.82 (0.66, 1.02) | 1.10 (0.87, 1.38) | 0.93 (0.75, 1.16) | 0.87 (0.73, 1.04) | 0.85 (0.71, 1.02) | 0.89 (0.73, 1.08) |
| Service support and other service positions | 1.11 (0.91, 1.34) | 1.14 (1.02, 1.28) | 1.11 (0.98, 1.26) | 1.13 (1.00, 1.29) | 1.16 (1.00, 1.35) | 1.13 (0.97, 1.31) | 1.15 (0.99, 1.34) | 1.15 (0.99, 1.34) | 1.15 (1.02, 1.30) | 1.15 (1.01, 1.30) | 1.19 (1.04, 1.36) |
| **Trades, transport and equipment operators and related** | 1.02 (0.90, 1.17) | 1.03 (0.96, 1.12) | 1.05 (0.96, 1.14) | 1.05 (0.96, 1.14) | 1.10 (0.99, 1.22) | 1.15 (1.04, 1.27) | 1.06 (0.95, 1.18) | 1.02 (0.92, 1.14) | 1.06 (0.97, 1.15) | 1.04 (0.96, 1.14) | 1.04 (0.95, 1.15) |
| Industrial, electrical and construction trades | 0.98 (0.81, 1.18) | 1.05 (0.94, 1.17) | 1.12 (0.99, 1.27) | 1.11 (0.98, 1.25) | 1.22 (1.05, 1.41) | 1.08 (0.93, 1.25) | 1.11 (0.95, 1.29) | 0.99 (0.85, 1.15) | 1.10 (0.98, 1.23) | 1.11 (0.98, 1.26) | 1.04 (0.91, 1.18) |
| Maintenance and equipment operation trades | 1.03 (0.79, 1.33) | 0.96 (0.82, 1.11) | 0.93 (0.79, 1.10) | 0.94 (0.79, 1.11) | 1.03 (0.84, 1.25) | 1.06 (0.87, 1.30) | 0.87 (0.71, 1.07) | 0.99 (0.81, 1.21) | 0.92 (0.79, 1.08) | 0.93 (0.79, 1.10) | 0.96 (0.80, 1.15) |
| Other installers, repairers and servicers and material handlers | 1.33 (0.78, 2.26) | 1.15 (0.83, 1.59) | 1.22 (0.86, 1.73) | 1.22 (0.86, 1.74) | 1.30 (0.85, 1.98) | 1.25 (0.82, 1.88) | 1.19 (0.78, 1.83) | 1.20 (0.78, 1.86) | 1.28 (0.92, 1.78) | 1.22 (0.86, 1.75) | 1.31 (0.88, 1.95) |
| Transport and heavy equipment operation and related maintenance | 1.04 (0.80, 1.33) | 0.97 (0.84, 1.12) | 0.98 (0.83, 1.16) | 1.01 (0.86, 1.19) | 0.92 (0.76, 1.12) | 1.12 (0.92, 1.36) | 1.04 (0.85, 1.28) | 1.00 (0.82, 1.22) | 0.98 (0.84, 1.15) | 0.99 (0.84, 1.17) | 0.99 (0.83, 1.19) |
| Trades helpers, construction labourers and related | 1.02 (0.61, 1.69) | 1.41 (1.05, 1.89) | 1.02 (0.73, 1.42) | 1.00 (0.72, 1.40) | 0.97 (0.65, 1.44) | 1.80 (1.22, 2.67) | 1.35 (0.90, 2.03) | 1.34 (0.90, 1.98) | 1.35 (0.98, 1.85) | 1.00 (0.72, 1.41) | 1.44 (1.01, 2.06) |
| **Natural resources, agriculture and related production** | 1.04 (0.78, 1.38) | 1.11 (0.94, 1.30) | 1.10 (0.91, 1.33) | 1.12 (0.93, 1.35) | 1.02 (0.82, 1.27) | 1.44 (1.15, 1.79) | 1.32 (1.05, 1.66) | 1.04 (0.84, 1.30) | 1.18 (0.99, 1.40) | 1.11 (0.92, 1.34) | 1.06 (0.87, 1.30) |
| Supervisors and technical positions in natural resources, agriculture and related production | 1.47 (0.91, 2.38) | 1.14 (0.87, 1.50) | 1.06 (0.78, 1.45) | 1.08 (0.79, 1.48) | 1.16 (0.80, 1.67) | 1.14 (0.78, 1.64) | 1.16 (0.79, 1.70) | 1.35 (0.94, 1.96) | 1.16 (0.86, 1.57) | 1.08 (0.79, 1.48) | 1.34 (0.96, 1.87) |
| Workers in natural resources, agriculture and related production | 1.06 (0.67, 1.69) | 1.06 (0.81, 1.38) | 1.25 (0.93, 1.69) | 1.30 (0.96, 1.76) | 1.04 (0.72, 1.48) | 1.64 (1.15, 2.33) | 1.41 (0.97, 2.03) | 1.10 (0.77, 1.57) | 1.34 (1.00, 1.78) | 1.28 (0.94, 1.74) | 1.13 (0.82, 1.56) |
| Harvesting, landscaping and natural resources labourers | 0.63 (0.36, 1.09) | 1.12 (0.81, 1.53) | 0.95 (0.66, 1.36) | 0.94 (0.65, 1.35) | 0.85 (0.55, 1.29) | 1.58 (1.03, 2.41) | 1.39 (0.89, 2.15) | 0.69 (0.45, 1.05) | 0.99 (0.71, 1.38) | 0.93 (0.65, 1.32) | 0.73 (0.50, 1.06) |
| **Manufacturing and utilities** | 1.02 (0.82, 1.26) | 1.00 (0.89, 1.13) | 0.90 (0.79, 1.04) | 0.90 (0.78, 1.03) | 0.94 (0.80, 1.11) | 0.84 (0.71, 1.00) | 1.00 (0.84, 1.18) | 1.03 (0.88, 1.22) | 0.93 (0.81, 1.06) | 0.91 (0.79, 1.05) | 0.97 (0.84, 1.13) |
| Processing, manufacturing and utilities supervisors and central control operators | 0.85 (0.58, 1.25) | 0.90 (0.72, 1.13) | 0.87 (0.68, 1.12) | 0.88 (0.68, 1.13) | 0.85 (0.63, 1.16) | 0.67 (0.50, 0.91) | 0.77 (0.56, 1.04) | 0.85 (0.63, 1.15) | 0.79 (0.62, 1.00) | 0.89 (0.69, 1.16) | 0.81 (0.62, 1.06) |
| Processing and manufacturing machine operators and related production workers | 0.91 (0.65, 1.28) | 0.99 (0.81, 1.21) | 0.80 (0.64, 1.00) | 0.80 (0.64, 1.01) | 0.99 (0.76, 1.29) | 0.88 (0.68, 1.15) | 1.09 (0.83, 1.43) | 0.92 (0.71, 1.20) | 0.93 (0.75, 1.15) | 0.82 (0.66, 1.03) | 0.88 (0.69, 1.12) |
| Assemblers in manufacturing | 1.24 (0.79, 1.93) | 1.07 (0.83, 1.38) | 1.21 (0.91, 1.62) | 1.18 (0.88, 1.58) | 1.13 (0.80, 1.59) | 1.04 (0.73, 1.47) | 1.14 (0.80, 1.63) | 1.25 (0.88, 1.76) | 1.17 (0.89, 1.54) | 1.18 (0.88, 1.57) | 1.21 (0.89, 1.65) |
| Labourers in processing, manufacturing and utilities | 1.66 (0.87, 3.14) | 1.19 (0.82, 1.72) | 0.84 (0.55, 1.28) | 0.81 (0.54, 1.24) | 0.72 (0.44, 1.18) | 0.92 (0.55, 1.54) | 1.17 (0.70, 1.95) | 1.74 (1.07, 2.86) | 0.89 (0.59, 1.33) | 0.80 (0.53, 1.22) | 1.44 (0.92, 2.27) |
| **Unemployed** | 0.97 (0.88, 1.07) | 1.09 (1.03, 1.16) | 1.01 (0.95, 1.08) | 1.00 (0.93, 1.06) | 0.99 (0.91, 1.07) | 0.97 (0.89, 1.05) | 0.97 (0.89, 1.05) | 1.01 (0.94, 1.10) | 1.00 (0.94, 1.07) | 1.00 (0.94, 1.07) | 1.00 (0.93, 1.07) |

Abbreviations: CHMS, Canadian Health Measures Survey; GMR, Geometric mean ratio; CI, Confidence interval; DF, Detection frequency; MEP, Mono-ethyl phthalate; MnBP, Mono-n-butyl phthalate; MEOHP, Mono-(2-ethyl-5-oxohexyl) phthalate; MEHHP, Mono-(2-ethyl-5-hydroxyhexyl) phthalate; MEHP, Mono-(2-ethylhexyl) phthalate; MCPP, Mono-(3-carboxypropyl) phthalate; MBzP, Mono-benzyl phthalate; MMP, Mono-methyl phthalate; MCHP, Mono-cyclohexyl phthalate; MiNP, Mono-isononyl phthalate; MOP, Mono-octyl phthalate; LMWP, Low molecular weight phthalate; HMWP, High molecular weight phthalate; DEHP, Di(2-ethylhexyl) phthalate.

^a^Based on geometric mean concentration units of μg/g creatinine.

^b^Based on geometric mean concentration units of nmol/g creatinine.

∑LMWP = Molar sum of MMP + MEP + MnBP + MCHP.

∑HMWP = Molar sum of MEOHP + MEHHP + MEHP + MiNP + MOP + MCPP + MBzP.

∑DEHP = Molar sum of MEOHP + MEHHP + MEHP.

∑Total = Molar Sum of MMP + MEP + MnBP + MCHP + MEOHP + MEHHP + MEHP + MiNP + MOP + MCPP + MBzP.

^c^Least-squares GMRs computed by multivariable linear regression, using occupational terms as binary indicators, adjusting for: age (continuous), sex, CHMS cycle, ethnicity, household income, smoking status, six dietary consumptions groups (meat and alternatives, dairy, grains, fruits and vegetables, seafood, bottled/canned beverages), drinking water type, and physical activity.

**Supplemental Table 7**. Association between occupations with creatinine-corrected concentrations of phthalate metabolites (DF <70%), CHMS (n = 4,259), 2007-2019.

| **Occupation**  **(Broad = Bold; Major = Unbolded)** | **OR**^a^ **(95% CI)** | | | |
| --- | --- | --- | --- | --- |
|  | **MMP** | **MCHP** | **MiNP** | **MOP** |
| **Management** | 0.99 (0.72, 1.33) | 0.97 (0.60, 1.50) | 0.74 (0.45, 1.18) | 0.51 (0.12, 1.41) |
| Senior management | 2.14 (0.29, 11.41) | 2.45 (0.12, 16.60) | - | - |
| Specialized middle management | 0.86 (0.52, 1.38) | 0.82 (0.38, 1.58) | 1.10 (0.48, 2.25) | 0.40 (0.02, 1.92) |
| Middle management in retail and wholesale trade and customer services | 1.23 (0.70, 2.04) | 0.94 (0.36, 2.04) | 1.11 (0.53, 2.14) | 0.64 (0.04, 3.17) |
| Middle management in trades, transportation, production and utilities | 0.87 (0.48, 1.50) | 1.17 (0.48, 2.44) | 0.19 (0.03, 0.62) | 0.62 (0.03, 3.08) |
| **Business, finance and administration** | 0.67 (0.50, 0.87) | 1.09 (0.77, 1.51) | 1.02 (0.70, 1.45) | 0.62 (0.27, 1.28) |
| Professionals in business and finance | 0.69 (0.43, 1.07) | 1.10 (0.58, 1.93) | 1.33 (0.74, 2.27) | 0.31 (0.02, 1.51) |
| Administrative and financial supervisors and administrative positions | 0.65 (0.40, 1.00) | 0.63 (0.32, 1.14) | 1.01 (0.53, 1.80) | 1.12 (0.32, 2.93) |
| Finance, insurance and related business administrative positions | 0.45 (0.11, 1.26) | 2.15 (0.78, 5.00) | - | - |
| Office support | 0.90 (0.48, 1.57) | 1.65 (0.88, 2.91) | 1.11 (0.41, 2.59) | 1.22 (0.29, 3.58) |
| Distribution, tracking and scheduling co-ordination | 0.71 (0.31, 1.44) | 0.89 (0.26, 2.24) | 1.17 (0.37, 3.03) | - |
| **Natural and applied sciences and related** | 1.27 (0.94, 1.70) | 1.00 (0.59, 1.59) | 1.15 (0.75, 1.72) | 1.07 (0.43, 2.33) |
| Professionals in natural and applied sciences | 1.17 (0.80, 1.68) | 0.89 (0.46, 1.58) | 1.10 (0.64, 1.83) | 1.44 (0.53, 3.35) |
| Technical positions related to natural and applied sciences | 1.39 (0.86, 2.17) | 1.20 (0.53, 2.40) | 1.20 (0.60, 2.23) | 0.43 (0.02, 2.11) |
| **Health** | 0.79 (0.53, 1.15) | 0.56 (0.28, 1.01) | 1.15 (0.71, 1.79) | 0.89 (0.26, 2.30) |
| Professionals in nursing | 0.88 (0.40, 1.73) | 0.38 (0.06, 1.26) | 1.06 (0.41, 2.43) | 2.19 (0.34, 8.13) |
| Professionals in health (except nursing) | 0.62 (0.26, 1.29) | 0.78 (0.19, 2.19) | 1.13 (0.50, 2.34) | - |
| Technical positions in health | 0.85 (0.39, 1.68) | 0.84 (0.25, 2.10) | 0.89 (0.32, 2.08) | 0.81 (0.04, 4.23) |
| Assisting in support of health services | 0.89 (0.38, 1.83) | 0.39 (0.06, 1.28) | 1.74 (0.59, 4.48) | 0.81 (0.04, 4.19) |
| **Education, law and social, community and government services** | 1.14 (0.85, 1.50) | 0.64 (0.39, 1.01) | 1.16 (0.78, 1.68) | 0.76 (0.28, 1.69) |
| Professionals in education services | 1.18 (0.77, 1.76) | 0.56 (0.25, 1.10) | 1.25 (0.69, 2.18) | 0.24 (0.01, 1.13) |
| Professionals in law and social, community and government services | 0.79 (0.40, 1.42) | 0.49 (0.12, 1.34) | 0.88 (0.39, 1.78) | 0.80 (0.04, 4.02) |
| Paraprofessionals in legal, social, community and education services | 1.48 (0.79, 2.61) | 0.77 (0.23, 1.93) | 2.05 (0.93, 4.28) | 1.92 (0.30, 7.08) |
| Front-line public protection services | 1.21 (0.43, 2.95) | 1.10 (0.17, 3.89) | 0.42 (0.02, 2.31) | 1.82 (0.10, 10.41) |
| Care providers and educational, legal and public protection support | 1.05 (0.39, 2.41) | 1.08 (0.25, 3.13) | 0.58 (0.09, 2.12) | 1.14 (0.06, 6.28) |
| **Art, culture, recreation and sport** | 0.97 (0.62, 1.46) | 1.10 (0.59, 1.89) | 1.52 (0.76, 2.86) | 2.82 (1.24, 5.79) |
| Professionals in art and culture | 1.03 (0.46, 2.03) | 0.82 (0.24, 2.05) | 0.91 (0.21, 2.81) | 2.59 (0.59, 7.96) |
| Technical positions in art, culture, recreation and sport | 0.94 (0.55, 1.55) | 1.27 (0.61, 2.40) | 1.91 (0.85, 4.02) | 2.77 (1.00, 6.47) |
| **Sales and service** | 1.14 (0.91, 1.42) | 0.92 (0.65, 1.28) | 0.91 (0.65, 1.25) | 1.13 (0.58, 2.08) |
| Retail sales supervisors and specialized sales | 0.83 (0.43, 1.46) | 0.86 (0.30, 1.96) | 0.69 (0.29, 1.42) | 1.74 (0.27, 6.29) |
| Service supervisors and specialized service | 0.97 (0.53, 1.66) | 0.41 (0.10, 1.11) | 1.72 (0.80, 3.49) | 2.26 (0.63, 6.23) |
| Sales representatives and salespersons - wholesale and retail trade | 1.23 (0.67, 2.14) | 0.93 (0.28, 2.33) | 1.29 (0.60, 2.54) | 4.26 (0.64, 16.26) |
| Service representatives and other customer and personal services | 1.24 (0.80, 1.86) | 1.04 (0.52, 1.89) | 1.12 (0.58, 2.03) | 1.23 (0.29, 3.60) |
| Sales support | 0.92 (0.47, 1.66) | 1.12 (0.46, 2.35) | 0.60 (0.14, 1.83) | 0.50 (0.03, 2.45) |
| Service support and other service positions | 1.32 (0.88, 1.93) | 1.10 (0.59, 1.88) | 0.46 (0.19, 0.97) | 0.25 (0.01, 1.17) |
| **Trades, transport and equipment operators and related** | 0.84 (0.63, 1.11) | 0.67 (0.40, 1.07) | 0.84 (0.54, 1.26) | 1.37 (0.61, 2.84) |
| Industrial, electrical and construction trades | 0.88 (0.58, 1.30) | 0.68 (0.31, 1.30) | 0.84 (0.41, 1.57) | 1.81 (0.65, 4.31) |
| Maintenance and equipment operation trades | 0.83 (0.46, 1.41) | 0.29 (0.05, 0.94) | 0.47 (0.16, 1.13) | 0.93 (0.14, 3.33) |
| Other installers, repairers and servicers and material handlers | 0.67 (0.16, 2.03) | - | 1.23 (0.34, 3.61) | 12.08 (0.51, 109.80) |
| Transport and heavy equipment operation and related maintenance | 0.76 (0.42, 1.30) | 1.32 (0.57, 2.67) | 0.77 (0.30, 1.68) | 0.56 (0.03, 2.76) |
| Trades helpers, construction labourers and related | 1.49 (0.55, 3.66) | 0.77 (0.04, 3.84) | 3.26 (0.89, 11.27) | - |
| **Natural resources, agriculture and related production** | 1.50 (0.85, 2.55) | 2.90 (1.43, 5.47) | 1.67 (0.70, 3.68) | 0.62 (0.03, 3.06) |
| Supervisors and technical positions in natural resources, agriculture and related production | 1.69 (0.64, 3.99) | 3.86 (1.23, 10.16) | 1.75 (0.45, 5.77) | - |
| Workers in natural resources, agriculture and related production | 1.75 (0.71, 3.97) | 3.05 (0.99, 7.84) | 1.36 (0.29, 4.75) | 2.34 (0.13, 12.50) |
| Harvesting, landscaping and natural resources labourers | 0.96 (0.27, 2.71) | 1.45 (0.22, 5.33) | 2.03 (0.27, 10.89) | - |
| **Manufacturing and utilities** | 1.38 (0.90, 2.06) | 1.28 (0.65, 2.29) | 1.04 (0.53, 1.93) | 0.38 (0.02, 1.81) |
| Processing, manufacturing and utilities supervisors and central control operators | 1.21 (0.53, 2.50) | 0.89 (0.14, 3.05) | 1.12 (0.40, 2.71) | - |
| Processing and manufacturing machine operators and related production workers | 1.42 (0.71, 2.67) | 1.95 (0.78, 4.22) | 1.05 (0.29, 3.02) | 1.19 (0.07, 6.06) |
| Assemblers in manufacturing | 1.26 (0.49, 2.88) | 0.82 (0.13, 2.82) | 0.37 (0.02, 2.04) | - |
| Labourers in processing, manufacturing and utilities | 1.85 (0.50, 5.69) | 0.84 (0.05, 4.45) | 3.04 (0.36, 20.34) | - |
| **Unemployed** | 1.03 (0.83, 1.29) | 1.45 (1.04, 2.00) | 0.93 (0.70, 1.24) | 1.23 (0.55, 2.55) |

Abbreviations: –, no data; CHMS, Canadian Health Measures Survey; OR, Odds ratio; CI, Confidence interval; DF, Detection frequency; MMP, Mono-methyl phthalate; MCHP, Mono-cyclohexyl phthalate; MiNP, Mono-isononyl phthalate; MOP, Mono-octyl phthalate.

^a^ORs computed by unconditional multivariable logistic regression, using above/below limit of detection as binary outcomes, adjusting for: age (continuous), sex, CHMS cycle, ethnicity, household income, smoking status, six dietary consumptions groups (meat and alternatives, dairy, grains, fruits and vegetables, seafood, bottled/canned beverages; categorical), drinking water type, and physical activity.

**Supplemental Table 8**. Association between occupations with creatinine-corrected concentrations of phthalate metabolites (DF ≥70%) and summary groups, among males (n = 2,135), CHMS, 2007-2019.

| **Occupation**  **(Broad = Bold; Major = Unbolded)** | **GMR**^c^ **(95% CI)** | | | | | | | | | | |
| --- | --- | --- | --- | --- | --- | --- | --- | --- | --- | --- | --- |
|  | **MEP**^a^ | **MnBP**^a^ | **MEOHP**^a^ | **MEHHP**^a^ | **MEHP**^a^ | **MCPP**^a^ | **MBzP**^a^ | **∑LMWP**^b^ | **∑HMWP**^b^ | **∑DEHP**^b^ | **∑Total**^b^ |
| **Management** | 0.97 (0.81, 1.17) | 0.89 (0.81, 0.99) | 1.06 (0.94, 1.19) | 1.07 (0.95, 1.21) | 1.08 (0.93, 1.24) | 1.05 (0.92, 1.21) | 0.97 (0.84, 1.12) | 0.92 (0.79, 1.06) | 1.04 (0.93, 1.17) | 1.07 (0.95, 1.21) | 0.95 (0.83, 1.08) |
| Senior management | 1.57 (0.58, 4.26) | 0.92 (0.50, 1.69) | 0.82 (0.43, 1.55) | 0.80 (0.42, 1.53) | 0.82 (0.36, 1.89) | 0.77 (0.37, 1.60) | 0.90 (0.41, 1.94) | 1.25 (0.54, 2.92) | 0.81 (0.42, 1.56) | 0.82 (0.41, 1.65) | 1.29 (0.56, 2.99) |
| Specialized middle management | 1.13 (0.84, 1.51) | 0.98 (0.83, 1.16) | 1.34 (1.12, 1.62) | 1.42 (1.18, 1.71) | 1.50 (1.20, 1.88) | 1.22 (0.99, 1.51) | 0.91 (0.73, 1.14) | 1.06 (0.84, 1.33) | 1.29 (1.09, 1.54) | 1.42 (1.18, 1.72) | 1.19 (0.97, 1.46) |
| Middle management in retail and wholesale trade and customer services | 0.96 (0.65, 1.42) | 0.80 (0.64, 1.00) | 0.82 (0.64, 1.05) | 0.82 (0.64, 1.06) | 0.88 (0.65, 1.18) | 0.95 (0.71, 1.28) | 0.99 (0.73, 1.35) | 0.85 (0.63, 1.16) | 0.83 (0.65, 1.05) | 0.82 (0.64, 1.06) | 0.78 (0.58, 1.03) |
| Middle management in trades, transportation, production and utilities | 0.82 (0.61, 1.10) | 0.88 (0.75, 1.04) | 0.97 (0.81, 1.17) | 0.94 (0.78, 1.14) | 0.87 (0.70, 1.09) | 0.97 (0.78, 1.20) | 1.02 (0.81, 1.28) | 0.82 (0.65, 1.03) | 0.96 (0.80, 1.15) | 0.94 (0.78, 1.13) | 0.83 (0.67, 1.02) |
| **Business, finance and administration** | 1.02 (0.85, 1.23) | 0.90 (0.81, 1.00) | 0.92 (0.81, 1.03) | 0.89 (0.79, 1.01) | 0.85 (0.74, 0.98) | 0.87 (0.76, 1.00) | 0.87 (0.75, 1.00) | 0.99 (0.86, 1.15) | 0.89 (0.79, 0.99) | 0.89 (0.79, 1.01) | 0.97 (0.85, 1.11) |
| Professionals in business and finance | 0.78 (0.60, 1.01) | 1.02 (0.87, 1.18) | 0.87 (0.73, 1.03) | 0.82 (0.69, 0.98) | 0.76 (0.62, 0.94) | 0.91 (0.75, 1.10) | 0.80 (0.65, 0.98) | 0.88 (0.71, 1.08) | 0.83 (0.70, 0.97) | 0.83 (0.70, 0.99) | 0.85 (0.70, 1.02) |
| Administrative and financial supervisors and administrative positions | 1.64 (1.12, 2.39) | 0.93 (0.75, 1.15) | 1.00 (0.78, 1.27) | 0.98 (0.76, 1.25) | 0.92 (0.69, 1.23) | 1.00 (0.76, 1.32) | 0.98 (0.73, 1.31) | 1.41 (1.04, 1.91) | 1.01 (0.80, 1.27) | 0.96 (0.76, 1.23) | 1.33 (1.01, 1.74) |
| Finance, insurance and related business administrative positions | 0.68 (0.32, 1.42) | 0.74 (0.49, 1.12) | 0.90 (0.56, 1.44) | 0.84 (0.52, 1.36) | 0.92 (0.52, 1.61) | 0.57 (0.33, 1.01) | 1.54 (0.87, 2.72) | 0.70 (0.39, 1.24) | 1.01 (0.64, 1.61) | 0.87 (0.54, 1.40) | 0.74 (0.43, 1.25) |
| Office support | 0.88 (0.41, 1.90) | 0.71 (0.46, 1.09) | 1.00 (0.61, 1.64) | 0.92 (0.56, 1.52) | 0.86 (0.48, 1.56) | 0.76 (0.43, 1.34) | 0.74 (0.41, 1.34) | 0.81 (0.45, 1.47) | 0.90 (0.57, 1.42) | 0.94 (0.58, 1.55) | 0.92 (0.54, 1.57) |
| Distribution, tracking and scheduling co-ordination | 1.31 (0.87, 1.98) | 0.78 (0.62, 0.98) | 0.97 (0.74, 1.26) | 1.01 (0.77, 1.31) | 1.02 (0.75, 1.40) | 0.83 (0.62, 1.12) | 0.85 (0.62, 1.17) | 1.09 (0.79, 1.49) | 0.91 (0.71, 1.16) | 0.99 (0.76, 1.29) | 1.04 (0.79, 1.38) |
| **Natural and applied sciences and related** | 0.83 (0.69, 0.98) | 0.91 (0.82, 1.00) | 0.98 (0.88, 1.10) | 1.00 (0.90, 1.12) | 1.02 (0.89, 1.16) | 0.92 (0.80, 1.04) | 1.00 (0.87, 1.14) | 0.88 (0.76, 1.00) | 1.02 (0.91, 1.13) | 1.00 (0.89, 1.11) | 0.92 (0.81, 1.04) |
| Professionals in natural and applied sciences | 0.83 (0.68, 1.03) | 0.89 (0.79, 1.00) | 0.96 (0.84, 1.10) | 0.98 (0.85, 1.12) | 0.97 (0.83, 1.14) | 0.89 (0.76, 1.04) | 1.01 (0.86, 1.19) | 0.88 (0.74, 1.03) | 1.00 (0.88, 1.13) | 0.97 (0.85, 1.11) | 0.90 (0.78, 1.04) |
| Technical positions related to natural and applied sciences | 0.84 (0.63, 1.12) | 0.96 (0.82, 1.13) | 1.02 (0.85, 1.23) | 1.05 (0.87, 1.26) | 1.10 (0.88, 1.37) | 0.99 (0.80, 1.22) | 0.98 (0.79, 1.23) | 0.90 (0.72, 1.12) | 1.05 (0.88, 1.25) | 1.05 (0.87, 1.26) | 0.97 (0.79, 1.18) |
| **Health** | 1.29 (0.92, 1.81) | 0.97 (0.80, 1.17) | 1.13 (0.91, 1.41) | 1.10 (0.89, 1.37) | 1.10 (0.84, 1.42) | 1.03 (0.80, 1.33) | 1.24 (0.95, 1.60) | 1.17 (0.90, 1.52) | 1.08 (0.87, 1.33) | 1.10 (0.88, 1.37) | 1.17 (0.92, 1.50) |
| Professionals in nursing | 4.26 (1.26, 14.35) | 0.70 (0.35, 1.38) | 1.15 (0.53, 2.51) | 1.11 (0.51, 2.46) | 0.56 (0.15, 2.07) | 0.81 (0.33, 1.97) | 1.89 (0.74, 4.85) | 2.35 (0.91, 6.04) | 0.80 (0.29, 2.25) | 0.91 (0.30, 2.76) | 2.21 (0.68, 7.21) |
| Professionals in health (except nursing) | 1.15 (0.71, 1.85) | 1.00 (0.77, 1.31) | 1.32 (0.97, 1.78) | 1.28 (0.94, 1.74) | 1.30 (0.90, 1.86) | 1.21 (0.85, 1.72) | 1.38 (0.96, 1.99) | 1.13 (0.78, 1.64) | 1.31 (0.98, 1.75) | 1.28 (0.94, 1.73) | 1.28 (0.91, 1.80) |
| Technical positions in health | 1.07 (0.59, 1.94) | 0.90 (0.64, 1.25) | 0.77 (0.53, 1.13) | 0.75 (0.51, 1.11) | 0.86 (0.55, 1.36) | 0.91 (0.58, 1.43) | 1.02 (0.65, 1.62) | 0.92 (0.58, 1.46) | 0.74 (0.51, 1.06) | 0.77 (0.52, 1.12) | 0.81 (0.53, 1.24) |
| Assisting in support of health services | 1.55 (0.57, 4.21) | 1.27 (0.73, 2.22) | 1.67 (0.88, 3.16) | 1.65 (0.86, 3.15) | 1.27 (0.59, 2.72) | 0.89 (0.43, 1.85) | 0.94 (0.43, 2.02) | 1.61 (0.74, 3.49) | 1.43 (0.79, 2.60) | 1.60 (0.85, 3.04) | 1.73 (0.87, 3.43) |
| **Education, law and social, community and government services** | 0.89 (0.71, 1.11) | 1.03 (0.91, 1.17) | 0.80 (0.70, 0.93) | 0.82 (0.71, 0.95) | 0.85 (0.71, 1.01) | 0.92 (0.77, 1.08) | 0.95 (0.79, 1.13) | 0.94 (0.79, 1.12) | 0.84 (0.73, 0.97) | 0.82 (0.71, 0.95) | 0.90 (0.77, 1.05) |
| Professionals in education services | 0.82 (0.59, 1.13) | 1.05 (0.88, 1.27) | 0.83 (0.67, 1.03) | 0.85 (0.69, 1.06) | 0.92 (0.71, 1.19) | 0.93 (0.73, 1.18) | 1.02 (0.79, 1.32) | 0.90 (0.70, 1.16) | 0.87 (0.71, 1.07) | 0.86 (0.69, 1.06) | 0.88 (0.70, 1.11) |
| Professionals in law and social, community and government services | 1.21 (0.79, 1.85) | 1.12 (0.88, 1.42) | 0.84 (0.64, 1.10) | 0.90 (0.69, 1.19) | 0.83 (0.60, 1.14) | 0.95 (0.69, 1.30) | 0.87 (0.63, 1.21) | 1.15 (0.83, 1.60) | 0.88 (0.68, 1.13) | 0.87 (0.66, 1.13) | 1.06 (0.79, 1.42) |
| Paraprofessionals in legal, social, community and education services | 0.70 (0.32, 1.50) | 0.82 (0.54, 1.27) | 1.34 (0.82, 2.20) | 1.39 (0.84, 2.29) | 1.34 (0.74, 2.41) | 1.21 (0.69, 2.12) | 1.25 (0.69, 2.26) | 0.77 (0.42, 1.40) | 1.36 (0.86, 2.15) | 1.34 (0.82, 2.20) | 0.95 (0.56, 1.61) |
| Front-line public protection services | 0.67 (0.40, 1.11) | 0.95 (0.71, 1.27) | 0.72 (0.52, 1.00) | 0.69 (0.50, 0.96) | 0.78 (0.52, 1.16) | 0.95 (0.65, 1.38) | 0.86 (0.58, 1.28) | 0.75 (0.51, 1.12) | 0.75 (0.55, 1.02) | 0.72 (0.52, 1.01) | 0.68 (0.47, 0.97) |
| Care providers and educational, legal and public protection support | 2.59 (0.77, 8.73) | 0.93 (0.47, 1.83) | 0.30 (0.14, 0.65) | 0.28 (0.13, 0.61) | 0.28 (0.11, 0.70) | 0.28 (0.12, 0.69) | 0.62 (0.24, 1.59) | 2.19 (0.85, 5.64) | 0.33 (0.16, 0.69) | 0.28 (0.13, 0.61) | 1.60 (0.69, 3.70) |
| **Art, culture, recreation and sport** | 1.02 (0.77, 1.35) | 1.04 (0.89, 1.21) | 1.05 (0.88, 1.25) | 1.07 (0.89, 1.28) | 1.03 (0.83, 1.28) | 1.03 (0.84, 1.27) | 0.95 (0.77, 1.18) | 1.03 (0.83, 1.28) | 1.04 (0.87, 1.23) | 1.06 (0.88, 1.27) | 1.03 (0.85, 1.26) |
| Professionals in art and culture | 1.74 (1.00, 3.00) | 1.16 (0.86, 1.58) | 1.27 (0.89, 1.80) | 1.27 (0.89, 1.82) | 1.28 (0.84, 1.94) | 0.92 (0.61, 1.39) | 1.00 (0.66, 1.53) | 1.54 (1.01, 2.36) | 1.18 (0.84, 1.65) | 1.27 (0.89, 1.80) | 1.53 (1.04, 2.25) |
| Technical positions in art, culture, recreation and sport | 0.85 (0.62, 1.17) | 1.00 (0.83, 1.19) | 0.98 (0.80, 1.21) | 1.00 (0.81, 1.23) | 0.96 (0.75, 1.23) | 1.07 (0.84, 1.35) | 0.93 (0.73, 1.20) | 0.89 (0.69, 1.15) | 0.99 (0.82, 1.20) | 0.99 (0.81, 1.22) | 0.90 (0.72, 1.13) |
| **Sales and service** | 1.07 (0.92, 1.25) | 1.02 (0.93, 1.11) | 1.02 (0.93, 1.13) | 1.05 (0.95, 1.16) | 1.05 (0.93, 1.18) | 1.03 (0.92, 1.15) | 1.00 (0.89, 1.13) | 1.05 (0.93, 1.18) | 1.03 (0.93, 1.13) | 1.05 (0.95, 1.16) | 1.05 (0.94, 1.17) |
| Retail sales supervisors and specialized sales | 1.09 (0.77, 1.55) | 0.97 (0.80, 1.19) | 1.05 (0.84, 1.32) | 1.10 (0.87, 1.38) | 1.23 (0.93, 1.61) | 1.03 (0.80, 1.35) | 0.86 (0.66, 1.13) | 1.11 (0.84, 1.46) | 1.05 (0.84, 1.30) | 1.12 (0.89, 1.40) | 1.11 (0.86, 1.43) |
| Service supervisors and specialized service | 0.92 (0.60, 1.40) | 0.99 (0.78, 1.25) | 1.02 (0.78, 1.33) | 0.98 (0.74, 1.28) | 0.94 (0.68, 1.29) | 0.87 (0.64, 1.18) | 0.72 (0.52, 1.00) | 0.85 (0.62, 1.18) | 0.90 (0.70, 1.16) | 0.98 (0.74, 1.28) | 0.84 (0.63, 1.13) |
| Sales representatives and salespersons - wholesale and retail trade | 1.20 (0.82, 1.76) | 0.89 (0.72, 1.11) | 0.97 (0.76, 1.24) | 1.00 (0.78, 1.28) | 0.91 (0.68, 1.23) | 0.80 (0.60, 1.07) | 0.98 (0.73, 1.32) | 1.10 (0.81, 1.49) | 0.93 (0.73, 1.18) | 0.98 (0.76, 1.26) | 1.07 (0.81, 1.41) |
| Service representatives and other customer and personal services | 0.86 (0.61, 1.21) | 0.94 (0.78, 1.14) | 0.97 (0.78, 1.21) | 0.98 (0.79, 1.22) | 1.01 (0.77, 1.32) | 1.08 (0.84, 1.38) | 1.05 (0.81, 1.37) | 0.87 (0.67, 1.13) | 1.01 (0.81, 1.24) | 1.01 (0.81, 1.26) | 0.91 (0.71, 1.16) |
| Sales support | 1.42 (0.87, 2.32) | 1.13 (0.85, 1.48) | 1.32 (0.96, 1.82) | 1.39 (1.01, 1.91) | 1.02 (0.70, 1.49) | 1.05 (0.73, 1.50) | 1.23 (0.84, 1.80) | 1.24 (0.84, 1.81) | 1.24 (0.92, 1.68) | 1.31 (0.95, 1.80) | 1.32 (0.93, 1.86) |
| Service support and other service positions | 1.09 (0.83, 1.44) | 1.15 (0.99, 1.34) | 0.98 (0.82, 1.17) | 1.00 (0.84, 1.20) | 1.09 (0.88, 1.35) | 1.18 (0.97, 1.45) | 1.17 (0.95, 1.45) | 1.13 (0.91, 1.40) | 1.07 (0.91, 1.27) | 1.02 (0.85, 1.22) | 1.11 (0.92, 1.35) |
| **Trades, transport and equipment operators and related** | 1.03 (0.90, 1.19) | 1.05 (0.97, 1.14) | 1.05 (0.96, 1.15) | 1.05 (0.95, 1.15) | 1.09 (0.98, 1.21) | 1.17 (1.06, 1.30) | 1.07 (0.96, 1.19) | 1.04 (0.93, 1.17) | 1.05 (0.97, 1.15) | 1.04 (0.95, 1.14) | 1.06 (0.96, 1.17) |
| Industrial, electrical and construction trades | 0.95 (0.78, 1.16) | 1.07 (0.96, 1.20) | 1.14 (1.01, 1.30) | 1.13 (0.99, 1.28) | 1.23 (1.06, 1.44) | 1.09 (0.95, 1.27) | 1.13 (0.97, 1.32) | 0.99 (0.85, 1.16) | 1.11 (0.99, 1.25) | 1.13 (1.00, 1.29) | 1.03 (0.90, 1.19) |
| Maintenance and equipment operation trades | 1.04 (0.80, 1.36) | 0.97 (0.84, 1.13) | 0.92 (0.78, 1.10) | 0.92 (0.77, 1.10) | 1.01 (0.82, 1.24) | 1.07 (0.88, 1.31) | 0.88 (0.71, 1.08) | 1.01 (0.82, 1.25) | 0.91 (0.77, 1.07) | 0.92 (0.77, 1.09) | 0.98 (0.81, 1.18) |
| Other installers, repairers and servicers and material handlers | 1.31 (0.74, 2.33) | 1.07 (0.76, 1.51) | 1.19 (0.82, 1.73) | 1.22 (0.84, 1.77) | 1.25 (0.80, 1.97) | 1.29 (0.85, 1.97) | 1.26 (0.81, 1.97) | 1.17 (0.73, 1.89) | 1.30 (0.91, 1.85) | 1.20 (0.82, 1.76) | 1.32 (0.85, 2.03) |
| Transport and heavy equipment operation and related maintenance | 1.10 (0.84, 1.44) | 0.99 (0.85, 1.15) | 0.97 (0.81, 1.15) | 1.00 (0.84, 1.19) | 0.87 (0.71, 1.08) | 1.13 (0.93, 1.38) | 1.04 (0.84, 1.28) | 1.05 (0.85, 1.30) | 0.97 (0.82, 1.15) | 0.97 (0.82, 1.16) | 1.03 (0.85, 1.26) |
| Trades helpers, construction labourers and related | 1.05 (0.61, 1.81) | 1.43 (1.05, 1.94) | 0.97 (0.68, 1.37) | 0.94 (0.66, 1.35) | 0.97 (0.63, 1.49) | 1.85 (1.24, 2.76) | 1.23 (0.81, 1.88) | 1.40 (0.92, 2.14) | 1.27 (0.90, 1.77) | 0.95 (0.67, 1.37) | 1.46 (0.99, 2.14) |
| **Natural resources, agriculture and related production** | 1.11 (0.80, 1.54) | 1.13 (0.94, 1.35) | 1.03 (0.84, 1.27) | 1.03 (0.84, 1.28) | 0.93 (0.72, 1.20) | 1.43 (1.13, 1.82) | 1.34 (1.04, 1.72) | 1.11 (0.86, 1.43) | 1.12 (0.92, 1.37) | 1.03 (0.83, 1.27) | 1.09 (0.87, 1.37) |
| Supervisors and technical positions in natural resources, agriculture and related production | 1.47 (0.88, 2.45) | 1.11 (0.83, 1.48) | 0.97 (0.70, 1.35) | 0.99 (0.71, 1.37) | 1.01 (0.68, 1.50) | 1.09 (0.75, 1.58) | 1.14 (0.77, 1.70) | 1.36 (0.91, 2.02) | 1.09 (0.79, 1.49) | 0.98 (0.71, 1.36) | 1.29 (0.90, 1.85) |
| Workers in natural resources, agriculture and related production | 1.41 (0.78, 2.55) | 1.19 (0.85, 1.65) | 1.19 (0.82, 1.74) | 1.20 (0.82, 1.76) | 0.93 (0.58, 1.48) | 1.86 (1.21, 2.87) | 1.51 (0.95, 2.38) | 1.43 (0.91, 2.27) | 1.32 (0.92, 1.91) | 1.21 (0.82, 1.79) | 1.38 (0.91, 2.10) |
| Harvesting, landscaping and natural resources labourers | 0.61 (0.34, 1.08) | 1.08 (0.78, 1.49) | 0.97 (0.67, 1.40) | 0.95 (0.65, 1.38) | 0.85 (0.55, 1.32) | 1.52 (0.99, 2.31) | 1.41 (0.90, 2.19) | 0.67 (0.43, 1.04) | 0.99 (0.70, 1.40) | 0.94 (0.65, 1.36) | 0.71 (0.48, 1.06) |
| **Manufacturing and utilities** | 1.04 (0.81, 1.33) | 1.04 (0.91, 1.20) | 0.92 (0.79, 1.08) | 0.92 (0.78, 1.08) | 0.93 (0.76, 1.12) | 0.85 (0.70, 1.02) | 1.04 (0.86, 1.26) | 1.09 (0.90, 1.33) | 0.95 (0.81, 1.10) | 0.93 (0.79, 1.10) | 1.03 (0.86, 1.23) |
| Processing, manufacturing and utilities supervisors and central control operators | 0.97 (0.64, 1.48) | 0.94 (0.74, 1.19) | 0.85 (0.65, 1.12) | 0.86 (0.65, 1.13) | 0.84 (0.60, 1.17) | 0.66 (0.48, 0.91) | 0.91 (0.66, 1.27) | 0.95 (0.68, 1.33) | 0.80 (0.62, 1.05) | 0.88 (0.67, 1.16) | 0.89 (0.66, 1.21) |
| Processing and manufacturing machine operators and related production workers | 0.88 (0.59, 1.33) | 1.08 (0.86, 1.37) | 0.78 (0.60, 1.02) | 0.79 (0.60, 1.03) | 0.91 (0.66, 1.24) | 0.93 (0.69, 1.26) | 1.12 (0.82, 1.54) | 0.94 (0.68, 1.30) | 0.93 (0.73, 1.19) | 0.80 (0.62, 1.04) | 0.88 (0.66, 1.17) |
| Assemblers in manufacturing | 1.09 (0.64, 1.86) | 1.08 (0.80, 1.46) | 1.37 (0.97, 1.94) | 1.34 (0.94, 1.89) | 1.28 (0.85, 1.92) | 1.12 (0.76, 1.66) | 1.07 (0.70, 1.61) | 1.21 (0.80, 1.84) | 1.25 (0.91, 1.72) | 1.33 (0.94, 1.88) | 1.25 (0.87, 1.81) |
| Labourers in processing, manufacturing and utilities | 2.17 (0.96, 4.90) | 1.21 (0.77, 1.91) | 0.98 (0.58, 1.65) | 0.95 (0.56, 1.62) | 0.70 (0.37, 1.30) | 0.78 (0.43, 1.42) | 1.15 (0.61, 2.16) | 2.35 (1.25, 4.42) | 0.94 (0.58, 1.54) | 0.92 (0.55, 1.56) | 1.91 (1.09, 3.35) |
| **Unemployed** | 1.01 (0.86, 1.19) | 1.09 (0.99, 1.20) | 1.04 (0.94, 1.16) | 1.01 (0.90, 1.12) | 0.99 (0.88, 1.13) | 0.92 (0.81, 1.03) | 0.95 (0.84, 1.08) | 1.00 (0.88, 1.14) | 1.00 (0.90, 1.10) | 1.01 (0.91, 1.13) | 0.98 (0.88, 1.10) |

Abbreviations: CHMS, Canadian Health Measures Survey; GMR, Geometric mean ratio; CI, Confidence interval; DF, Detection frequency; MEP, Mono-ethyl phthalate; MnBP, Mono-n-butyl phthalate; MEOHP, Mono-(2-ethyl-5-oxohexyl) phthalate; MEHHP, Mono-(2-ethyl-5-hydroxyhexyl) phthalate; MEHP, Mono-(2-ethylhexyl) phthalate; MCPP, Mono-(3-carboxypropyl) phthalate; MBzP, Mono-benzyl phthalate; MMP, Mono-methyl phthalate; MCHP, Mono-cyclohexyl phthalate; MiNP, Mono-isononyl phthalate; MOP, Mono-octyl phthalate; LMWP, Low molecular weight phthalate; HMWP, High molecular weight phthalate; DEHP, Di(2-ethylhexyl) phthalate.

^a^Based on geometric mean concentration units of μg/g creatinine.

^b^Based on geometric mean concentration units of nmol/g creatinine.

∑LMWP = Molar sum of MMP + MEP + MnBP + MCHP.

∑HMWP = Molar sum of MEOHP + MEHHP + MEHP + MiNP + MOP + MCPP + MBzP.

∑DEHP = Molar sum of MEOHP + MEHHP + MEHP.

∑Total = Molar Sum of MMP + MEP + MnBP + MCHP + MEOHP + MEHHP + MEHP + MiNP + MOP + MCPP + MBzP.

^c^Least-squares GMRs computed by multivariable linear regression, using occupational terms as binary indicators, adjusting for: age (continuous), CHMS cycle, ethnicity, household income, smoking status, six dietary consumptions groups (meat and alternatives, dairy, grains, fruits and vegetables, seafood, bottled/canned beverages), drinking water type, and physical activity.

**Supplemental Table 9**. Association between occupations with creatinine-corrected concentrations of phthalate metabolites (DF ≥70%) and summary groups, among females (n = 2,124), CHMS, 2007-2019.

| **Occupation**  **(Broad = Bold; Major = Unbolded)** | **GMR**^c^ **(95% CI)** | | | | | | | | | | |
| --- | --- | --- | --- | --- | --- | --- | --- | --- | --- | --- | --- |
|  | **MEP**^a^ | **MnBP**^a^ | **MEOHP**^a^ | **MEHHP**^a^ | **MEHP**^a^ | **MCPP**^a^ | **MBzP**^a^ | **∑LMWP**^b^ | **∑HMWP**^b^ | **∑DEHP**^b^ | **∑Total**^b^ |
| **Management** | 1.03 (0.82, 1.29) | 0.89 (0.77, 1.01) | 1.13 (0.98, 1.32) | 1.15 (0.99, 1.33) | 1.02 (0.86, 1.22) | 0.94 (0.78, 1.13) | 0.88 (0.73, 1.05) | 0.99 (0.83, 1.18) | 1.03 (0.90, 1.19) | 1.13 (0.98, 1.32) | 1.02 (0.87, 1.20) |
| Senior management | - | - | - | - | - | - | - | - | - | - | - |
| Specialized middle management | 1.02 (0.72, 1.42) | 0.92 (0.75, 1.13) | 1.03 (0.82, 1.28) | 1.04 (0.83, 1.30) | 1.00 (0.77, 1.30) | 0.94 (0.72, 1.24) | 0.90 (0.68, 1.20) | 1.05 (0.80, 1.37) | 0.95 (0.77, 1.17) | 1.03 (0.82, 1.29) | 1.01 (0.80, 1.28) |
| Middle management in retail and wholesale trade and customer services | 1.01 (0.73, 1.41) | 0.90 (0.74, 1.09) | 1.30 (1.04, 1.61) | 1.31 (1.05, 1.63) | 1.09 (0.85, 1.41) | 0.96 (0.73, 1.25) | 0.90 (0.68, 1.18) | 0.94 (0.73, 1.22) | 1.18 (0.96, 1.45) | 1.29 (1.04, 1.60) | 1.04 (0.83, 1.31) |
| Middle management in trades, transportation, production and utilities | 1.12 (0.60, 2.08) | 0.75 (0.52, 1.09) | 0.94 (0.62, 1.43) | 0.96 (0.63, 1.45) | 0.84 (0.51, 1.38) | 0.87 (0.52, 1.44) | 0.74 (0.44, 1.25) | 0.97 (0.60, 1.57) | 0.87 (0.58, 1.29) | 0.95 (0.62, 1.46) | 0.98 (0.63, 1.53) |
| **Business, finance and administration** | 1.13 (0.99, 1.30) | 0.96 (0.89, 1.04) | 1.04 (0.95, 1.13) | 1.04 (0.95, 1.14) | 1.00 (0.90, 1.11) | 1.06 (0.95, 1.18) | 0.81 (0.73, 0.91) | 1.07 (0.96, 1.19) | 0.97 (0.89, 1.05) | 1.03 (0.94, 1.13) | 1.06 (0.96, 1.17) |
| Professionals in business and finance | 1.05 (0.80, 1.38) | 0.91 (0.77, 1.07) | 1.00 (0.83, 1.20) | 1.00 (0.84, 1.20) | 1.05 (0.85, 1.30) | 0.99 (0.79, 1.24) | 0.72 (0.57, 0.90) | 1.01 (0.82, 1.25) | 0.92 (0.77, 1.09) | 1.00 (0.84, 1.20) | 1.05 (0.87, 1.27) |
| Administrative and financial supervisors and administrative positions | 1.18 (0.97, 1.45) | 0.94 (0.83, 1.05) | 0.98 (0.86, 1.12) | 0.98 (0.86, 1.12) | 0.94 (0.80, 1.10) | 1.04 (0.88, 1.23) | 0.80 (0.67, 0.94) | 1.11 (0.95, 1.30) | 0.92 (0.81, 1.04) | 0.97 (0.85, 1.11) | 1.07 (0.93, 1.23) |
| Finance, insurance and related business administrative positions | 0.92 (0.60, 1.41) | 1.06 (0.82, 1.36) | 0.92 (0.69, 1.23) | 0.96 (0.72, 1.27) | 0.94 (0.68, 1.31) | 0.88 (0.62, 1.26) | 1.20 (0.84, 1.71) | 0.90 (0.64, 1.26) | 0.97 (0.74, 1.27) | 0.93 (0.70, 1.24) | 0.91 (0.67, 1.24) |
| Office support | 1.16 (0.90, 1.49) | 1.01 (0.87, 1.17) | 1.19 (1.00, 1.40) | 1.17 (0.99, 1.39) | 1.09 (0.90, 1.33) | 1.24 (1.01, 1.52) | 0.94 (0.76, 1.16) | 1.07 (0.88, 1.30) | 1.15 (0.98, 1.35) | 1.19 (1.00, 1.41) | 1.09 (0.91, 1.30) |
| Distribution, tracking and scheduling co-ordination | 1.01 (0.61, 1.69) | 1.08 (0.80, 1.46) | 1.03 (0.73, 1.45) | 1.07 (0.76, 1.50) | 0.96 (0.64, 1.45) | 0.86 (0.57, 1.30) | 0.75 (0.49, 1.15) | 1.05 (0.71, 1.55) | 0.86 (0.62, 1.19) | 1.04 (0.74, 1.47) | 0.98 (0.69, 1.40) |
| **Natural and applied sciences and related** | 0.90 (0.68, 1.19) | 1.07 (0.90, 1.26) | 0.95 (0.78, 1.14) | 0.92 (0.76, 1.11) | 1.00 (0.80, 1.24) | 0.81 (0.64, 1.02) | 0.96 (0.76, 1.22) | 0.99 (0.79, 1.23) | 0.92 (0.77, 1.10) | 0.93 (0.77, 1.12) | 1.00 (0.82, 1.23) |
| Professionals in natural and applied sciences | 0.78 (0.54, 1.12) | 0.85 (0.68, 1.05) | 0.91 (0.72, 1.16) | 0.89 (0.70, 1.14) | 0.96 (0.73, 1.28) | 0.74 (0.54, 0.99) | 0.75 (0.56, 1.01) | 0.81 (0.61, 1.08) | 0.84 (0.67, 1.06) | 0.91 (0.71, 1.15) | 0.86 (0.67, 1.11) |
| Technical positions related to natural and applied sciences | 1.11 (0.72, 1.72) | 1.48 (1.14, 1.91) | 1.00 (0.75, 1.34) | 0.96 (0.71, 1.28) | 1.04 (0.74, 1.46) | 0.93 (0.65, 1.33) | 1.39 (0.96, 2.02) | 1.29 (0.93, 1.81) | 1.04 (0.78, 1.37) | 0.97 (0.73, 1.30) | 1.25 (0.92, 1.70) |
| **Health** | 1.04 (0.87, 1.25) | 0.87 (0.79, 0.97) | 0.93 (0.82, 1.04) | 0.91 (0.81, 1.03) | 0.96 (0.83, 1.10) | 0.97 (0.83, 1.12) | 1.25 (1.08, 1.45) | 0.95 (0.82, 1.09) | 1.00 (0.89, 1.12) | 0.92 (0.81, 1.03) | 0.94 (0.83, 1.07) |
| Professionals in nursing | 1.10 (0.81, 1.49) | 0.97 (0.80, 1.16) | 1.01 (0.82, 1.23) | 0.99 (0.81, 1.22) | 1.09 (0.86, 1.39) | 1.07 (0.83, 1.37) | 1.29 (1.00, 1.67) | 1.04 (0.82, 1.32) | 1.10 (0.90, 1.34) | 1.01 (0.82, 1.24) | 1.06 (0.85, 1.31) |
| Professionals in health (except nursing) | 0.94 (0.65, 1.36) | 0.96 (0.77, 1.21) | 0.95 (0.74, 1.22) | 0.92 (0.72, 1.18) | 1.07 (0.80, 1.43) | 0.76 (0.56, 1.03) | 1.35 (0.99, 1.84) | 0.91 (0.68, 1.21) | 0.97 (0.77, 1.24) | 0.95 (0.74, 1.22) | 0.84 (0.65, 1.10) |
| Technical positions in health | 0.93 (0.65, 1.33) | 0.78 (0.63, 0.96) | 0.76 (0.60, 0.96) | 0.74 (0.58, 0.94) | 0.68 (0.52, 0.90) | 0.89 (0.67, 1.20) | 1.01 (0.75, 1.36) | 0.83 (0.63, 1.09) | 0.81 (0.65, 1.01) | 0.74 (0.58, 0.93) | 0.80 (0.63, 1.02) |
| Assisting in support of health services | 1.16 (0.82, 1.64) | 0.83 (0.68, 1.02) | 1.01 (0.80, 1.28) | 1.01 (0.80, 1.28) | 1.03 (0.79, 1.35) | 1.14 (0.86, 1.52) | 1.27 (0.95, 1.70) | 1.01 (0.77, 1.32) | 1.10 (0.89, 1.37) | 1.01 (0.80, 1.27) | 1.06 (0.84, 1.35) |
| **Education, law and social, community and government services** | 0.86 (0.73, 1.01) | 1.12 (1.02, 1.23) | 1.05 (0.95, 1.17) | 1.05 (0.95, 1.17) | 1.08 (0.96, 1.23) | 1.12 (0.98, 1.28) | 1.22 (1.07, 1.39) | 0.93 (0.83, 1.05) | 1.11 (1.00, 1.23) | 1.06 (0.95, 1.18) | 0.96 (0.86, 1.07) |
| Professionals in education services | 0.80 (0.64, 1.01) | 1.23 (1.08, 1.41) | 1.13 (0.97, 1.32) | 1.12 (0.96, 1.31) | 1.19 (0.99, 1.42) | 1.03 (0.85, 1.25) | 1.30 (1.08, 1.58) | 0.92 (0.77, 1.10) | 1.17 (1.01, 1.36) | 1.14 (0.98, 1.33) | 0.96 (0.81, 1.13) |
| Professionals in law and social, community and government services | 0.94 (0.68, 1.31) | 0.92 (0.76, 1.12) | 1.04 (0.84, 1.30) | 1.05 (0.84, 1.31) | 1.13 (0.87, 1.46) | 1.15 (0.87, 1.52) | 0.96 (0.72, 1.26) | 0.91 (0.70, 1.17) | 1.03 (0.83, 1.29) | 1.03 (0.82, 1.28) | 0.93 (0.73, 1.19) |
| Paraprofessionals in legal, social, community and education services | 0.83 (0.60, 1.13) | 1.10 (0.91, 1.33) | 0.98 (0.80, 1.21) | 1.01 (0.82, 1.25) | 0.98 (0.76, 1.26) | 1.09 (0.84, 1.41) | 1.27 (0.98, 1.65) | 0.92 (0.72, 1.18) | 1.07 (0.88, 1.31) | 1.01 (0.82, 1.26) | 0.93 (0.74, 1.16) |
| Front-line public protection services | 0.60 (0.11, 3.10) | 1.05 (0.40, 2.79) | 2.20 (0.74, 6.59) | 1.89 (0.63, 5.66) | 1.18 (0.33, 4.23) | 3.07 (0.46, 20.45) | 1.64 (0.42, 6.47) | 0.70 (0.20, 2.49) | 10.24 (2.42, 43.31) | 1.91 (0.64, 5.68) | 3.38 (0.70, 16.41) |
| Care providers and educational, legal and public protection support | 1.12 (0.74, 1.69) | 1.10 (0.86, 1.40) | 0.87 (0.66, 1.14) | 0.87 (0.66, 1.14) | 0.85 (0.62, 1.17) | 1.33 (0.95, 1.86) | 1.12 (0.80, 1.58) | 1.09 (0.79, 1.49) | 0.96 (0.74, 1.24) | 0.86 (0.66, 1.14) | 1.03 (0.77, 1.36) |
| **Art, culture, recreation and sport** | 1.03 (0.79, 1.34) | 1.10 (0.93, 1.28) | 1.05 (0.88, 1.25) | 1.06 (0.88, 1.26) | 1.17 (0.95, 1.43) | 1.11 (0.89, 1.38) | 1.01 (0.81, 1.26) | 1.11 (0.90, 1.37) | 1.05 (0.89, 1.24) | 1.06 (0.89, 1.27) | 1.11 (0.92, 1.33) |
| Professionals in art and culture | 1.01 (0.68, 1.50) | 1.22 (0.96, 1.53) | 1.26 (0.97, 1.64) | 1.21 (0.93, 1.57) | 1.35 (1.00, 1.83) | 1.22 (0.89, 1.69) | 0.97 (0.70, 1.35) | 1.18 (0.87, 1.60) | 1.18 (0.92, 1.50) | 1.24 (0.95, 1.60) | 1.26 (0.96, 1.64) |
| Technical positions in art, culture, recreation and sport | 1.04 (0.73, 1.48) | 1.00 (0.81, 1.23) | 0.90 (0.71, 1.14) | 0.94 (0.74, 1.19) | 1.03 (0.78, 1.35) | 1.01 (0.75, 1.35) | 1.04 (0.77, 1.40) | 1.05 (0.80, 1.39) | 0.96 (0.77, 1.19) | 0.94 (0.74, 1.18) | 0.99 (0.77, 1.27) |
| **Sales and service** | 1.10 (0.96, 1.27) | 0.96 (0.88, 1.04) | 0.93 (0.85, 1.02) | 0.94 (0.86, 1.04) | 0.91 (0.82, 1.02) | 0.91 (0.81, 1.02) | 1.00 (0.89, 1.12) | 1.03 (0.92, 1.15) | 0.94 (0.86, 1.03) | 0.93 (0.85, 1.02) | 1.00 (0.91, 1.11) |
| Retail sales supervisors and specialized sales | 1.13 (0.77, 1.66) | 1.00 (0.80, 1.26) | 0.95 (0.73, 1.22) | 0.96 (0.74, 1.23) | 1.01 (0.75, 1.36) | 0.98 (0.72, 1.34) | 0.78 (0.56, 1.07) | 1.03 (0.77, 1.38) | 0.88 (0.69, 1.12) | 0.95 (0.74, 1.22) | 0.91 (0.70, 1.18) |
| Service supervisors and specialized service | 1.08 (0.79, 1.50) | 0.86 (0.71, 1.04) | 0.97 (0.78, 1.20) | 1.01 (0.82, 1.26) | 0.92 (0.71, 1.18) | 1.00 (0.77, 1.31) | 0.91 (0.70, 1.19) | 0.99 (0.77, 1.27) | 0.98 (0.80, 1.21) | 0.98 (0.79, 1.22) | 0.99 (0.80, 1.24) |
| Sales representatives and salespersons - wholesale and retail trade | 1.06 (0.69, 1.65) | 0.90 (0.69, 1.16) | 0.89 (0.66, 1.19) | 0.86 (0.64, 1.15) | 1.00 (0.71, 1.40) | 0.79 (0.56, 1.13) | 0.99 (0.69, 1.43) | 0.92 (0.65, 1.30) | 0.96 (0.73, 1.26) | 0.88 (0.66, 1.17) | 0.96 (0.71, 1.30) |
| Service representatives and other customer and personal services | 1.30 (1.00, 1.67) | 0.91 (0.78, 1.06) | 0.86 (0.72, 1.01) | 0.87 (0.73, 1.03) | 0.81 (0.66, 0.99) | 0.91 (0.74, 1.13) | 1.03 (0.83, 1.27) | 1.11 (0.91, 1.35) | 0.89 (0.76, 1.04) | 0.85 (0.72, 1.01) | 1.05 (0.88, 1.25) |
| Sales support | 0.75 (0.53, 1.06) | 0.96 (0.78, 1.18) | 0.69 (0.55, 0.87) | 0.69 (0.55, 0.87) | 0.66 (0.50, 0.86) | 0.73 (0.55, 0.97) | 1.02 (0.77, 1.36) | 0.82 (0.62, 1.06) | 0.72 (0.58, 0.90) | 0.67 (0.53, 0.85) | 0.73 (0.57, 0.92) |
| Service support and other service positions | 1.10 (0.84, 1.44) | 1.14 (0.97, 1.34) | 1.27 (1.06, 1.52) | 1.29 (1.07, 1.54) | 1.24 (1.00, 1.54) | 1.05 (0.84, 1.32) | 1.15 (0.92, 1.44) | 1.16 (0.94, 1.43) | 1.25 (1.05, 1.49) | 1.30 (1.08, 1.56) | 1.27 (1.05, 1.54) |
| **Trades, transport and equipment operators and related** | 0.67 (0.40, 1.13) | 0.85 (0.63, 1.15) | 1.00 (0.71, 1.41) | 0.98 (0.70, 1.38) | 1.09 (0.73, 1.62) | 1.05 (0.69, 1.60) | 1.09 (0.71, 1.67) | 0.68 (0.46, 1.01) | 1.04 (0.76, 1.43) | 0.99 (0.71, 1.39) | 0.78 (0.55, 1.10) |
| Industrial, electrical and construction trades | 0.68 (0.24, 1.94) | 0.71 (0.38, 1.33) | 0.59 (0.30, 1.19) | 0.55 (0.27, 1.11) | 0.65 (0.29, 1.47) | 1.01 (0.43, 2.36) | 1.16 (0.48, 2.76) | 0.65 (0.29, 1.45) | 0.77 (0.40, 1.48) | 0.57 (0.29, 1.14) | 0.72 (0.35, 1.46) |
| Maintenance and equipment operation trades | 0.45 (0.14, 1.43) | 0.71 (0.36, 1.42) | 1.31 (0.60, 2.83) | 1.38 (0.63, 3.00) | 1.56 (0.63, 3.85) | 1.38 (0.54, 3.58) | 1.01 (0.38, 2.67) | 0.48 (0.20, 1.18) | 1.44 (0.70, 2.96) | 1.37 (0.63, 2.95) | 0.66 (0.30, 1.46) |
| Other installers, repairers and servicers and material handlers | 2.98 (0.57, 15.47) | 2.09 (0.79, 5.54) | 1.39 (0.46, 4.16) | 1.27 (0.42, 3.80) | 1.50 (0.42, 5.41) | 1.00 (0.26, 3.83) | 0.56 (0.14, 2.22) | 2.45 (0.69, 8.67) | 1.04 (0.37, 2.88) | 1.33 (0.45, 3.94) | 1.94 (0.63, 5.92) |
| Transport and heavy equipment operation and related maintenance | 0.63 (0.28, 1.45) | 0.79 (0.49, 1.29) | 1.00 (0.58, 1.73) | 0.99 (0.57, 1.72) | 1.26 (0.66, 2.39) | 0.97 (0.49, 1.90) | 0.87 (0.44, 1.73) | 0.65 (0.34, 1.22) | 0.87 (0.52, 1.45) | 1.01 (0.58, 1.75) | 0.67 (0.38, 1.17) |
| Trades helpers, construction labourers and related | 0.47 (0.09, 2.43) | 1.02 (0.38, 2.70) | 1.53 (0.51, 4.61) | 1.54 (0.51, 4.64) | 0.73 (0.20, 2.63) | 0.97 (0.25, 3.74) | 5.32 (1.35, 21.00) | 0.56 (0.16, 1.98) | 2.42 (0.87, 6.73) | 1.43 (0.48, 4.25) | 1.00 (0.33, 3.08) |
| **Natural resources, agriculture and related production** | 0.70 (0.36, 1.34) | 0.98 (0.67, 1.45) | 1.48 (0.96, 2.27) | 1.56 (1.01, 2.40) | 1.47 (0.89, 2.43) | 1.43 (0.85, 2.43) | 1.35 (0.79, 2.32) | 0.73 (0.45, 1.21) | 1.46 (0.98, 2.19) | 1.51 (0.98, 2.32) | 0.90 (0.58, 1.40) |
| Supervisors and technical positions in natural resources, agriculture and related production | 1.54 (0.30, 8.00) | 1.49 (0.56, 3.93) | 3.88 (1.30, 11.58) | 3.80 (1.27, 11.37) | 6.51 (1.82, 23.33) | 1.99 (0.52, 7.60) | 1.87 (0.48, 7.37) | 1.30 (0.37, 4.62) | 3.19 (1.15, 8.84) | 3.98 (1.34, 11.79) | 2.10 (0.69, 6.42) |
| Workers in natural resources, agriculture and related production | 0.60 (0.29, 1.27) | 0.86 (0.56, 1.33) | 1.30 (0.79, 2.12) | 1.40 (0.86, 2.30) | 1.20 (0.67, 2.13) | 1.25 (0.69, 2.29) | 1.23 (0.67, 2.28) | 0.65 (0.37, 1.15) | 1.29 (0.82, 2.05) | 1.34 (0.82, 2.18) | 0.77 (0.47, 1.27) |
| Harvesting, landscaping and natural resources labourers | 0.60 (0.06, 6.22) | 1.62 (0.41, 6.43) | 0.74 (0.16, 3.51) | 0.71 (0.15, 3.37) | 0.55 (0.09, 3.38) | 2.81 (0.42, 18.72) | 1.70 (0.24, 11.83) | 0.76 (0.13, 4.52) | 1.03 (0.24, 4.36) | 0.69 (0.15, 3.21) | 0.77 (0.16, 3.73) |
| **Manufacturing and utilities** | 0.93 (0.61, 1.42) | 0.91 (0.71, 1.17) | 0.89 (0.67, 1.18) | 0.85 (0.64, 1.13) | 1.00 (0.72, 1.39) | 0.88 (0.62, 1.27) | 0.94 (0.66, 1.34) | 0.87 (0.63, 1.20) | 0.90 (0.68, 1.18) | 0.87 (0.65, 1.15) | 0.80 (0.59, 1.08) |
| Processing, manufacturing and utilities supervisors and central control operators | 0.38 (0.13, 1.07) | 0.68 (0.37, 1.27) | 1.28 (0.64, 2.56) | 1.24 (0.62, 2.49) | 1.28 (0.57, 2.88) | 0.87 (0.37, 2.05) | 0.26 (0.11, 0.63) | 0.42 (0.19, 0.94) | 0.85 (0.44, 1.62) | 1.24 (0.62, 2.46) | 0.49 (0.24, 0.98) |
| Processing and manufacturing machine operators and related production workers | 0.92 (0.48, 1.76) | 0.82 (0.56, 1.21) | 0.86 (0.56, 1.33) | 0.82 (0.53, 1.26) | 1.19 (0.70, 2.01) | 0.77 (0.44, 1.33) | 1.05 (0.61, 1.80) | 0.83 (0.50, 1.36) | 0.91 (0.60, 1.38) | 0.86 (0.55, 1.35) | 0.84 (0.53, 1.32) |
| Assemblers in manufacturing | 1.69 (0.74, 3.85) | 1.07 (0.66, 1.75) | 0.87 (0.50, 1.50) | 0.84 (0.49, 1.46) | 0.76 (0.40, 1.45) | 0.85 (0.41, 1.74) | 1.45 (0.73, 2.88) | 1.35 (0.72, 2.55) | 0.96 (0.56, 1.66) | 0.83 (0.48, 1.44) | 1.11 (0.61, 2.02) |
| Labourers in processing, manufacturing and utilities | 0.94 (0.33, 2.67) | 1.25 (0.67, 2.32) | 0.70 (0.35, 1.40) | 0.65 (0.32, 1.31) | 0.79 (0.35, 1.77) | 1.49 (0.57, 3.86) | 1.28 (0.53, 3.06) | 0.99 (0.44, 2.22) | 0.83 (0.40, 1.72) | 0.67 (0.34, 1.34) | 0.75 (0.34, 1.67) |
| **Unemployed** | 0.94 (0.83, 1.07) | 1.09 (1.01, 1.18) | 0.99 (0.91, 1.08) | 0.98 (0.90, 1.07) | 0.98 (0.89, 1.09) | 1.01 (0.91, 1.12) | 0.98 (0.88, 1.09) | 1.03 (0.93, 1.13) | 1.00 (0.92, 1.09) | 0.99 (0.91, 1.08) | 1.01 (0.92, 1.11) |

Abbreviations: –, no data; CHMS, Canadian Health Measures Survey; GMR, Geometric mean ratio; CI, Confidence interval; DF, Detection frequency; MEP, Mono-ethyl phthalate; MnBP, Mono-n-butyl phthalate; MEOHP, Mono-(2-ethyl-5-oxohexyl) phthalate; MEHHP, Mono-(2-ethyl-5-hydroxyhexyl) phthalate; MEHP, Mono-(2-ethylhexyl) phthalate; MCPP, Mono-(3-carboxypropyl) phthalate; MBzP, Mono-benzyl phthalate; MMP, Mono-methyl phthalate; MCHP, Mono-cyclohexyl phthalate; MiNP, Mono-isononyl phthalate; MOP, Mono-octyl phthalate; LMWP, Low molecular weight phthalate; HMWP, High molecular weight phthalate; DEHP, Di(2-ethylhexyl) phthalate.

^a^Based on geometric mean concentration units of μg/g creatinine.

^b^Based on geometric mean concentration units of nmol/g creatinine.

∑LMWP = Molar sum of MMP + MEP + MnBP + MCHP.

∑HMWP = Molar sum of MEOHP + MEHHP + MEHP + MiNP + MOP + MCPP + MBzP.

∑DEHP = Molar sum of MEOHP + MEHHP + MEHP.

∑Total = Molar Sum of MMP + MEP + MnBP + MCHP + MEOHP + MEHHP + MEHP + MiNP + MOP + MCPP + MBzP.

^c^Least-squares GMRs computed by multivariable linear regression, using occupational terms as binary indicators, adjusting for: age (continuous), CHMS cycle, ethnicity, household income, smoking status, six dietary consumptions groups (meat and alternatives, dairy, grains, fruits and vegetables, seafood, bottled/canned beverages), drinking water type, and physical activity.

**Supplemental Table 10**. Association between occupations with creatinine- and specific gravity-corrected concentrations of phthalate metabolite summary groups, CHMS (n = 3,072), 2009-2019.

| **Occupation**  **(Broad = Bold; Major = Unbolded)** | **GMR**^c^ **(95% CI)** | | | | | | | |
| --- | --- | --- | --- | --- | --- | --- | --- | --- |
|  | **Creatinine Corrected** | | | | **Specific Gravity Corrected** | | | |
|  | **∑LMWP**^a^ | **∑HMWP**^a^ | **∑DEHP**^a^ | **∑Total**^a^ | **∑LMWP**^b^ | **∑HMWP**^b^ | **∑DEHP**^b^ | **∑Total**^b^ |
| **Management** | 0.92 (0.80, 1.05) | 0.97 (0.88, 1.08) | 1.05 (0.95, 1.16) | 0.93 (0.83, 1.05) | 0.93 (0.81, 1.07) | 0.99 (0.90, 1.10) | 1.07 (0.96, 1.18) | 0.95 (0.83, 1.08) |
| Senior management | 0.85 (0.30, 2.38) | 0.62 (0.28, 1.36) | 0.62 (0.28, 1.37) | 0.86 (0.28, 2.62) | 1.02 (0.35, 3.00) | 0.71 (0.32, 1.58) | 0.71 (0.32, 1.58) | 1.16 (0.36, 3.74) |
| Specialized middle management | 1.04 (0.84, 1.29) | 1.10 (0.94, 1.30) | 1.25 (1.06, 1.48) | 1.10 (0.91, 1.34) | 1.04 (0.83, 1.30) | 1.10 (0.94, 1.30) | 1.25 (1.06, 1.48) | 1.09 (0.89, 1.34) |
| Middle management in retail and wholesale trade and customer services | 0.86 (0.69, 1.09) | 0.87 (0.73, 1.04) | 0.93 (0.78, 1.10) | 0.83 (0.67, 1.03) | 0.89 (0.70, 1.13) | 0.90 (0.75, 1.08) | 0.95 (0.80, 1.13) | 0.86 (0.69, 1.07) |
| Middle management in trades, transportation, production and utilities | 0.86 (0.68, 1.08) | 0.96 (0.80, 1.14) | 0.99 (0.82, 1.18) | 0.87 (0.70, 1.07) | 0.87 (0.68, 1.11) | 0.98 (0.82, 1.17) | 1.00 (0.84, 1.20) | 0.88 (0.71, 1.10) |
| **Business, finance and administration** | 1.03 (0.93, 1.15) | 0.94 (0.87, 1.02) | 1.00 (0.92, 1.08) | 1.03 (0.94, 1.13) | 1.03 (0.92, 1.14) | 0.93 (0.86, 1.01) | 0.99 (0.91, 1.07) | 1.02 (0.92, 1.12) |
| Professionals in business and finance | 0.95 (0.80, 1.13) | 0.89 (0.79, 1.02) | 0.96 (0.84, 1.09) | 0.96 (0.83, 1.12) | 0.97 (0.82, 1.16) | 0.92 (0.80, 1.05) | 0.99 (0.87, 1.13) | 0.98 (0.84, 1.16) |
| Administrative and financial supervisors and administrative positions | 1.13 (0.95, 1.33) | 0.95 (0.84, 1.08) | 0.99 (0.87, 1.13) | 1.08 (0.93, 1.25) | 1.09 (0.91, 1.30) | 0.91 (0.80, 1.04) | 0.95 (0.84, 1.08) | 1.04 (0.88, 1.21) |
| Finance, insurance and related business administrative positions | 0.91 (0.63, 1.31) | 0.92 (0.69, 1.21) | 0.89 (0.68, 1.17) | 0.92 (0.66, 1.29) | 0.93 (0.63, 1.36) | 0.94 (0.71, 1.25) | 0.90 (0.68, 1.18) | 0.95 (0.67, 1.36) |
| Office support | 1.04 (0.82, 1.33) | 1.07 (0.89, 1.30) | 1.11 (0.91, 1.35) | 1.10 (0.87, 1.38) | 1.04 (0.81, 1.35) | 1.07 (0.88, 1.30) | 1.10 (0.91, 1.34) | 1.08 (0.85, 1.38) |
| Distribution, tracking and scheduling co-ordination | 1.06 (0.77, 1.47) | 0.95 (0.74, 1.22) | 1.09 (0.85, 1.42) | 1.07 (0.79, 1.43) | 1.00 (0.71, 1.41) | 0.89 (0.68, 1.15) | 1.02 (0.79, 1.32) | 0.99 (0.73, 1.35) |
| **Natural and applied sciences and related** | 0.98 (0.85, 1.12) | 1.01 (0.91, 1.12) | 0.98 (0.88, 1.08) | 0.99 (0.87, 1.12) | 1.03 (0.89, 1.18) | 1.07 (0.96, 1.19) | 1.02 (0.92, 1.14) | 1.05 (0.92, 1.19) |
| Professionals in natural and applied sciences | 0.92 (0.77, 1.10) | 0.98 (0.86, 1.12) | 0.95 (0.83, 1.08) | 0.92 (0.79, 1.08) | 0.97 (0.81, 1.17) | 1.05 (0.92, 1.20) | 1.00 (0.87, 1.14) | 0.99 (0.83, 1.17) |
| Technical positions related to natural and applied sciences | 1.07 (0.87, 1.32) | 1.05 (0.89, 1.23) | 1.02 (0.87, 1.20) | 1.10 (0.91, 1.33) | 1.10 (0.89, 1.38) | 1.09 (0.92, 1.29) | 1.06 (0.90, 1.24) | 1.13 (0.93, 1.38) |
| **Health** | 0.92 (0.80, 1.07) | 1.01 (0.91, 1.13) | 0.93 (0.84, 1.05) | 0.93 (0.82, 1.06) | 0.93 (0.80, 1.08) | 1.01 (0.90, 1.14) | 0.94 (0.84, 1.05) | 0.94 (0.81, 1.07) |
| Professionals in nursing | 1.12 (0.86, 1.45) | 1.28 (1.04, 1.57) | 1.13 (0.92, 1.39) | 1.18 (0.92, 1.51) | 1.08 (0.82, 1.42) | 1.24 (1.00, 1.53) | 1.09 (0.88, 1.35) | 1.14 (0.88, 1.48) |
| Professionals in health (except nursing) | 0.87 (0.67, 1.13) | 1.06 (0.87, 1.29) | 1.04 (0.85, 1.27) | 0.87 (0.68, 1.10) | 0.88 (0.67, 1.16) | 1.05 (0.86, 1.29) | 1.05 (0.86, 1.28) | 0.87 (0.68, 1.12) |
| Technical positions in health | 0.77 (0.59, 1.01) | 0.77 (0.63, 0.95) | 0.72 (0.59, 0.88) | 0.75 (0.59, 0.95) | 0.79 (0.60, 1.05) | 0.79 (0.64, 0.97) | 0.74 (0.60, 0.91) | 0.77 (0.60, 0.99) |
| Assisting in support of health services | 1.03 (0.74, 1.42) | 1.02 (0.79, 1.30) | 0.91 (0.71, 1.17) | 1.06 (0.79, 1.43) | 1.04 (0.74, 1.46) | 1.03 (0.80, 1.33) | 0.92 (0.72, 1.19) | 1.08 (0.79, 1.47) |
| **Education, law and social, community and government services** | 0.93 (0.83, 1.05) | 1.03 (0.94, 1.13) | 0.97 (0.89, 1.07) | 0.95 (0.85, 1.06) | 0.93 (0.82, 1.05) | 1.03 (0.94, 1.13) | 0.97 (0.89, 1.07) | 0.95 (0.84, 1.06) |
| Professionals in education services | 0.98 (0.83, 1.17) | 1.16 (1.01, 1.33) | 1.08 (0.94, 1.24) | 1.03 (0.87, 1.21) | 0.98 (0.82, 1.18) | 1.15 (1.00, 1.33) | 1.07 (0.94, 1.23) | 1.02 (0.86, 1.21) |
| Professionals in law and social, community and government services | 0.95 (0.76, 1.20) | 0.87 (0.73, 1.05) | 0.91 (0.76, 1.08) | 0.93 (0.75, 1.16) | 0.89 (0.70, 1.13) | 0.84 (0.70, 1.02) | 0.87 (0.73, 1.04) | 0.89 (0.71, 1.12) |
| Paraprofessionals in legal, social, community and education services | 0.84 (0.64, 1.09) | 1.18 (0.96, 1.46) | 1.08 (0.88, 1.33) | 0.88 (0.69, 1.12) | 0.85 (0.64, 1.12) | 1.18 (0.96, 1.46) | 1.08 (0.87, 1.33) | 0.87 (0.68, 1.13) |
| Front-line public protection services | 0.62 (0.39, 0.96) | 0.69 (0.48, 0.99) | 0.64 (0.45, 0.91) | 0.56 (0.37, 0.86) | 0.68 (0.42, 1.09) | 0.78 (0.54, 1.13) | 0.73 (0.51, 1.04) | 0.64 (0.41, 0.99) |
| Care providers and educational, legal and public protection support | 1.21 (0.84, 1.74) | 0.86 (0.65, 1.14) | 0.81 (0.60, 1.08) | 1.17 (0.84, 1.63) | 1.29 (0.88, 1.89) | 0.92 (0.69, 1.23) | 0.87 (0.65, 1.16) | 1.25 (0.88, 1.77) |
| **Art, culture, recreation and sport** | 0.99 (0.82, 1.21) | 0.99 (0.86, 1.15) | 1.00 (0.86, 1.16) | 0.99 (0.83, 1.18) | 1.03 (0.84, 1.27) | 1.05 (0.90, 1.22) | 1.05 (0.90, 1.22) | 1.03 (0.86, 1.24) |
| Professionals in art and culture | 1.10 (0.80, 1.52) | 0.95 (0.74, 1.21) | 0.96 (0.75, 1.23) | 1.11 (0.83, 1.48) | 1.12 (0.80, 1.57) | 0.99 (0.77, 1.28) | 1.00 (0.78, 1.28) | 1.14 (0.84, 1.55) |
| Technical positions in art, culture, recreation and sport | 0.94 (0.74, 1.20) | 1.02 (0.85, 1.23) | 1.02 (0.85, 1.23) | 0.93 (0.74, 1.15) | 0.98 (0.76, 1.27) | 1.08 (0.90, 1.30) | 1.08 (0.89, 1.29) | 0.97 (0.77, 1.22) |
| **Sales and service** | 1.00 (0.91, 1.10) | 0.99 (0.92, 1.06) | 0.97 (0.90, 1.05) | 0.99 (0.90, 1.08) | 1.01 (0.91, 1.12) | 1.00 (0.93, 1.08) | 0.99 (0.92, 1.07) | 1.00 (0.91, 1.10) |
| Retail sales supervisors and specialized sales | 0.88 (0.70, 1.12) | 0.95 (0.79, 1.14) | 1.01 (0.84, 1.22) | 0.84 (0.68, 1.04) | 0.86 (0.67, 1.10) | 0.92 (0.76, 1.11) | 0.99 (0.82, 1.19) | 0.81 (0.65, 1.02) |
| Service supervisors and specialized service | 0.91 (0.72, 1.16) | 0.99 (0.82, 1.20) | 1.02 (0.85, 1.24) | 0.93 (0.74, 1.15) | 0.95 (0.73, 1.22) | 1.04 (0.86, 1.26) | 1.07 (0.88, 1.29) | 0.97 (0.77, 1.22) |
| Sales representatives and salespersons - wholesale and retail trade | 1.05 (0.83, 1.32) | 0.96 (0.80, 1.14) | 0.95 (0.79, 1.13) | 1.03 (0.83, 1.27) | 1.07 (0.83, 1.37) | 0.98 (0.81, 1.18) | 0.97 (0.81, 1.17) | 1.06 (0.84, 1.32) |
| Service representatives and other customer and personal services | 0.99 (0.81, 1.19) | 0.90 (0.78, 1.04) | 0.86 (0.74, 1.00) | 0.97 (0.81, 1.16) | 1.03 (0.84, 1.26) | 0.93 (0.80, 1.09) | 0.89 (0.77, 1.04) | 1.01 (0.84, 1.21) |
| Sales support | 1.03 (0.77, 1.37) | 0.87 (0.69, 1.09) | 0.88 (0.70, 1.11) | 0.95 (0.73, 1.24) | 1.10 (0.81, 1.50) | 0.94 (0.75, 1.19) | 0.96 (0.77, 1.21) | 1.03 (0.78, 1.36) |
| Service support and other service positions | 1.12 (0.93, 1.35) | 1.20 (1.04, 1.39) | 1.12 (0.97, 1.30) | 1.17 (0.98, 1.38) | 1.06 (0.87, 1.29) | 1.14 (0.98, 1.33) | 1.08 (0.93, 1.25) | 1.11 (0.93, 1.33) |
| **Trades, transport and equipment operators and related** | 1.09 (0.97, 1.24) | 1.04 (0.95, 1.15) | 1.06 (0.96, 1.17) | 1.10 (0.98, 1.23) | 1.03 (0.91, 1.18) | 0.98 (0.89, 1.07) | 1.00 (0.90, 1.09) | 1.03 (0.92, 1.16) |
| Industrial, electrical and construction trades | 1.01 (0.84, 1.21) | 1.06 (0.93, 1.21) | 1.08 (0.94, 1.24) | 1.03 (0.87, 1.21) | 0.96 (0.80, 1.17) | 1.00 (0.87, 1.15) | 1.02 (0.88, 1.17) | 0.97 (0.82, 1.15) |
| Maintenance and equipment operation trades | 1.10 (0.86, 1.40) | 0.86 (0.71, 1.04) | 0.94 (0.78, 1.13) | 1.02 (0.82, 1.28) | 1.05 (0.81, 1.35) | 0.83 (0.68, 1.00) | 0.90 (0.75, 1.09) | 0.98 (0.78, 1.24) |
| Other installers, repairers and servicers and material handlers | 1.17 (0.76, 1.81) | 1.29 (0.93, 1.77) | 1.23 (0.88, 1.70) | 1.30 (0.87, 1.93) | 1.15 (0.73, 1.81) | 1.26 (0.91, 1.75) | 1.20 (0.87, 1.67) | 1.31 (0.86, 1.98) |
| Transport and heavy equipment operation and related maintenance | 1.08 (0.85, 1.37) | 0.99 (0.82, 1.19) | 1.04 (0.86, 1.25) | 1.08 (0.87, 1.35) | 1.00 (0.78, 1.29) | 0.89 (0.74, 1.08) | 0.95 (0.79, 1.14) | 0.99 (0.78, 1.24) |
| Trades helpers, construction labourers and related | 1.39 (0.89, 2.18) | 1.56 (1.10, 2.21) | 1.21 (0.85, 1.73) | 1.62 (1.08, 2.44) | 1.37 (0.85, 2.18) | 1.52 (1.06, 2.17) | 1.18 (0.83, 1.69) | 1.57 (1.03, 2.42) |
| **Natural resources, agriculture and related production** | 1.02 (0.77, 1.35) | 1.25 (1.01, 1.55) | 1.14 (0.92, 1.42) | 1.05 (0.82, 1.35) | 0.99 (0.74, 1.32) | 1.21 (0.97, 1.50) | 1.10 (0.89, 1.37) | 1.01 (0.78, 1.32) |
| Supervisors and technical positions in natural resources, agriculture and related production | 1.20 (0.75, 1.91) | 1.30 (0.90, 1.87) | 1.15 (0.80, 1.65) | 1.29 (0.84, 1.97) | 1.10 (0.68, 1.79) | 1.19 (0.82, 1.72) | 1.06 (0.74, 1.51) | 1.18 (0.75, 1.84) |
| Workers in natural resources, agriculture and related production | 0.99 (0.66, 1.47) | 1.21 (0.89, 1.65) | 1.19 (0.86, 1.63) | 0.97 (0.67, 1.39) | 0.97 (0.64, 1.48) | 1.19 (0.86, 1.63) | 1.16 (0.85, 1.60) | 0.95 (0.65, 1.39) |
| Harvesting, landscaping and natural resources labourers | 0.82 (0.44, 1.54) | 1.24 (0.77, 2.00) | 1.03 (0.63, 1.68) | 0.89 (0.51, 1.57) | 0.83 (0.43, 1.62) | 1.26 (0.77, 2.05) | 1.04 (0.64, 1.71) | 0.91 (0.51, 1.64) |
| **Manufacturing and utilities** | 1.05 (0.86, 1.29) | 1.00 (0.85, 1.17) | 1.01 (0.86, 1.19) | 1.03 (0.85, 1.24) | 0.97 (0.78, 1.20) | 0.90 (0.77, 1.06) | 0.93 (0.79, 1.09) | 0.93 (0.77, 1.14) |
| Processing, manufacturing and utilities supervisors and central control operators | 0.88 (0.63, 1.22) | 0.88 (0.68, 1.14) | 0.99 (0.77, 1.29) | 0.86 (0.63, 1.16) | 0.86 (0.61, 1.21) | 0.86 (0.66, 1.12) | 0.97 (0.75, 1.26) | 0.84 (0.61, 1.14) |
| Processing and manufacturing machine operators and related production workers | 0.93 (0.67, 1.30) | 1.05 (0.82, 1.35) | 0.90 (0.70, 1.17) | 0.95 (0.70, 1.28) | 0.86 (0.60, 1.21) | 0.95 (0.74, 1.23) | 0.82 (0.63, 1.06) | 0.86 (0.63, 1.18) |
| Assemblers in manufacturing | 1.64 (1.00, 2.69) | 1.21 (0.81, 1.78) | 1.34 (0.91, 1.96) | 1.55 (0.98, 2.45) | 1.31 (0.78, 2.20) | 0.93 (0.62, 1.39) | 1.06 (0.72, 1.56) | 1.19 (0.74, 1.93) |
| Labourers in processing, manufacturing and utilities | 1.71 (0.82, 3.54) | 0.90 (0.49, 1.65) | 1.03 (0.58, 1.81) | 1.61 (0.79, 3.27) | 1.65 (0.77, 3.54) | 0.84 (0.45, 1.56) | 1.00 (0.57, 1.76) | 1.49 (0.71, 3.12) |
| **Unemployed** | 1.03 (0.95, 1.13) | 1.00 (0.94, 1.07) | 1.01 (0.94, 1.08) | 1.02 (0.94, 1.10) | 1.04 (0.96, 1.14) | 1.01 (0.95, 1.08) | 1.01 (0.95, 1.08) | 1.03 (0.95, 1.12) |

Abbreviations: CHMS, Canadian Health Measures Survey; GMR, Geometric mean ratio; CI, Confidence interval; MEP, Mono-ethyl phthalate; MnBP, Mono-n-butyl phthalate; MEOHP, Mono-(2-ethyl-5-oxohexyl) phthalate; MEHHP, Mono-(2-ethyl-5-hydroxyhexyl) phthalate; MEHP, Mono-(2-ethylhexyl) phthalate; MCPP, Mono-(3-carboxypropyl) phthalate; MBzP, Mono-benzyl phthalate; MMP, Mono-methyl phthalate; MCHP, Mono-cyclohexyl phthalate; MiNP, Mono-isononyl phthalate; MOP, Mono-octyl phthalate; LMWP, Low molecular weight phthalate; HMWP, High molecular weight phthalate; DEHP, Di(2-ethylhexyl) phthalate.

^a^Based on geometric mean concentration units of nmol/g creatinine

^b^Based on geometric mean concentration units of nmol/L

∑LMWP = Molar sum of MMP + MEP + MnBP + MCHP.

∑HMWP = Molar sum of MEOHP + MEHHP + MEHP + MiNP + MOP + MCPP + MBzP.

∑DEHP = Molar sum of MEOHP + MEHHP + MEHP.

∑Total = Molar Sum of MMP + MEP + MnBP + MCHP + MEOHP + MEHHP + MEHP + MiNP + MOP + MCPP + MBzP.

^c^Least-squares GMRs computed by multivariable linear regression, using occupational terms as binary indicators, adjusting for: age (continuous), sex, CHMS cycle, ethnicity, household income, smoking status, six dietary consumptions groups (meat and alternatives, dairy, grains, fruits and vegetables, seafood, bottled/canned beverages), drinking water type, and physical activity.

Analytic sample reduced to Cycles 2, 5, and 6 (n = 3,072) due to limited availability of specific gravity measurements.

**Supplemental Table 11**. Association between sector-level industry with creatinine-corrected concentrations of phthalate metabolites (DF ≥70%) and summary groups, overall (n = 4,259) and by sex (n_male_ = 2,135, n_female_ = 2,124), CHMS, 2007-2019.

| **Industry of Employment**  **(Sector-Level)** | | **GMR**^c^ **(95% CI)** | | | | | | | | | | | |
| --- | --- | --- | --- | --- | --- | --- | --- | --- | --- | --- | --- | --- | --- |
|  |  | **MEP**^a^ | | **MnBP**^a^ | **MEOHP**^a^ | **MEHHP**^a^ | **MEHP**^a^ | **MCPP**^a^ | **MBzP**^a^ | **∑LMWP**^b^ | **∑HMWP^b^** | **∑DEHP**^b^ | **∑Total**^b^ |
|  |  | | **OVERALL (n = 4,259)** | | | | | | | | | | |
| Agriculture, forestry, fishing and hunting | | 0.81 (0.60, 1.11) | | 1.00 (0.84, 1.19) | 1.14 (0.93, 1.39) | 1.12 (0.91, 1.37) | 1.00 (0.79, 1.27) | 1.18 (0.93, 1.49) | 1.44 (1.13, 1.85) | 0.89 (0.70, 1.13) | 1.21 (1.00, 1.47) | 1.12 (0.91, 1.37) | 0.96 (0.78, 1.20) |
| Mining, quarrying, and oil and gas extraction | | 0.92 (0.63, 1.33) | | 0.94 (0.76, 1.16) | 1.04 (0.82, 1.33) | 1.04 (0.82, 1.33) | 1.03 (0.77, 1.37) | 0.95 (0.72, 1.27) | 1.15 (0.85, 1.55) | 0.90 (0.67, 1.21) | 1.10 (0.88, 1.38) | 1.04 (0.82, 1.33) | 1.03 (0.79, 1.35) |
| Utilities | | 1.14 (0.71, 1.82) | | 1.27 (0.97, 1.68) | 1.09 (0.80, 1.49) | 1.07 (0.78, 1.46) | 1.11 (0.76, 1.60) | 0.90 (0.62, 1.30) | 1.42 (0.98, 2.07) | 1.14 (0.79, 1.65) | 1.19 (0.88, 1.60) | 1.11 (0.81, 1.52) | 1.19 (0.85, 1.67) |
| Construction | | 1.04 (0.88, 1.23) | | 1.02 (0.93, 1.13) | 1.07 (0.96, 1.19) | 1.07 (0.95, 1.19) | 1.05 (0.92, 1.20) | 1.11 (0.97, 1.26) | 1.10 (0.96, 1.26) | 1.03 (0.90, 1.18) | 1.06 (0.95, 1.17) | 1.06 (0.95, 1.18) | 1.05 (0.94, 1.18) |
| Manufacturing | | 1.04 (0.90, 1.20) | | 0.92 (0.85, 1.00) | 0.91 (0.82, 0.99) | 0.92 (0.84, 1.01) | 0.98 (0.87, 1.09) | 0.95 (0.85, 1.06) | 0.90 (0.80, 1.01) | 1.01 (0.90, 1.13) | 0.91 (0.83, 0.99) | 0.92 (0.84, 1.01) | 0.97 (0.88, 1.07) |
| Wholesale trade | | 1.02 (0.82, 1.28) | | 0.93 (0.82, 1.06) | 1.09 (0.94, 1.26) | 1.10 (0.95, 1.28) | 1.09 (0.92, 1.30) | 0.88 (0.73, 1.05) | 1.06 (0.88, 1.27) | 0.99 (0.83, 1.19) | 1.09 (0.94, 1.25) | 1.10 (0.95, 1.28) | 1.05 (0.89, 1.23) |
| Retail trade | | 0.98 (0.85, 1.15) | | 0.96 (0.88, 1.04) | 0.98 (0.89, 1.08) | 1.00 (0.90, 1.10) | 0.93 (0.83, 1.04) | 0.97 (0.86, 1.09) | 1.03 (0.91, 1.16) | 0.97 (0.86, 1.09) | 0.99 (0.90, 1.08) | 0.98 (0.89, 1.08) | 0.97 (0.87, 1.08) |
| Transportation and warehousing | | 1.25 (1.03, 1.52) | | 0.99 (0.89, 1.11) | 0.96 (0.84, 1.09) | 0.96 (0.85, 1.09) | 0.90 (0.78, 1.05) | 0.98 (0.84, 1.14) | 0.92 (0.79, 1.07) | 1.14 (0.98, 1.33) | 0.91 (0.81, 1.03) | 0.96 (0.84, 1.09) | 1.06 (0.92, 1.22) |
| Information and cultural industries | | 1.17 (0.89, 1.54) | | 0.95 (0.81, 1.12) | 1.17 (0.98, 1.41) | 1.17 (0.98, 1.41) | 1.15 (0.93, 1.42) | 1.10 (0.89, 1.36) | 0.86 (0.69, 1.07) | 1.06 (0.86, 1.32) | 1.07 (0.90, 1.26) | 1.16 (0.97, 1.39) | 1.04 (0.86, 1.27) |
| Finance and insurance | | 1.00 (0.82, 1.22) | | 1.04 (0.93, 1.17) | 1.04 (0.92, 1.19) | 1.03 (0.90, 1.17) | 1.12 (0.96, 1.30) | 1.00 (0.86, 1.17) | 0.82 (0.70, 0.96) | 1.05 (0.90, 1.22) | 1.00 (0.88, 1.13) | 1.04 (0.91, 1.18) | 1.04 (0.90, 1.20) |
| Real estate and rental and leasing | | 0.92 (0.66, 1.30) | | 0.95 (0.78, 1.15) | 1.09 (0.87, 1.36) | 1.07 (0.85, 1.33) | 1.04 (0.80, 1.35) | 1.18 (0.91, 1.53) | 0.88 (0.67, 1.16) | 0.94 (0.72, 1.23) | 1.03 (0.84, 1.27) | 1.09 (0.87, 1.36) | 1.01 (0.80, 1.28) |
| Professional, scientific and technical services | | 0.88 (0.76, 1.02) | | 0.91 (0.83, 0.99) | 0.93 (0.85, 1.02) | 0.96 (0.87, 1.06) | 0.98 (0.88, 1.10) | 0.91 (0.81, 1.02) | 0.85 (0.75, 0.95) | 0.89 (0.79, 1.00) | 0.95 (0.87, 1.04) | 0.95 (0.87, 1.05) | 0.92 (0.83, 1.01) |
| Administrative and support, waste management and remediation services | | 1.21 (0.98, 1.48) | | 1.01 (0.90, 1.14) | 0.94 (0.82, 1.07) | 0.93 (0.81, 1.07) | 0.90 (0.77, 1.05) | 1.05 (0.90, 1.24) | 0.93 (0.79, 1.10) | 1.12 (0.96, 1.31) | 0.93 (0.82, 1.06) | 0.93 (0.81, 1.06) | 1.08 (0.94, 1.25) |
| Educational services | | 0.85 (0.73, 1.00) | | 1.12 (1.03, 1.23) | 1.05 (0.94, 1.16) | 1.04 (0.94, 1.15) | 1.08 (0.95, 1.22) | 1.04 (0.92, 1.18) | 1.16 (1.02, 1.31) | 0.95 (0.84, 1.08) | 1.07 (0.97, 1.18) | 1.05 (0.95, 1.17) | 0.98 (0.88, 1.10) |
| Health care and social assistance | | 1.05 (0.92, 1.19) | | 0.95 (0.88, 1.02) | 0.97 (0.89, 1.05) | 0.97 (0.89, 1.05) | 0.98 (0.89, 1.08) | 1.00 (0.90, 1.11) | 1.16 (1.04, 1.28) | 1.00 (0.90, 1.11) | 0.99 (0.92, 1.08) | 0.96 (0.88, 1.05) | 0.99 (0.90, 1.08) |
| Arts, entertainment and recreation | | 1.02 (0.78, 1.33) | | 1.03 (0.89, 1.20) | 1.06 (0.89, 1.26) | 1.05 (0.88, 1.25) | 1.03 (0.84, 1.27) | 1.15 (0.94, 1.41) | 1.09 (0.88, 1.35) | 1.02 (0.83, 1.25) | 1.09 (0.93, 1.29) | 1.06 (0.89, 1.26) | 1.05 (0.87, 1.26) |
| Accommodation and food services | | 0.95 (0.79, 1.15) | | 0.96 (0.86, 1.06) | 1.09 (0.97, 1.23) | 1.10 (0.98, 1.24) | 1.06 (0.92, 1.22) | 1.04 (0.90, 1.20) | 0.93 (0.81, 1.08) | 0.95 (0.83, 1.10) | 1.03 (0.93, 1.16) | 1.09 (0.97, 1.23) | 0.98 (0.87, 1.12) |
| Other services (except public administration) | | 1.03 (0.84, 1.28) | | 1.10 (0.97, 1.24) | 0.91 (0.80, 1.05) | 0.92 (0.80, 1.05) | 0.96 (0.82, 1.13) | 1.11 (0.95, 1.31) | 1.10 (0.93, 1.30) | 1.04 (0.88, 1.22) | 0.97 (0.85, 1.11) | 0.91 (0.79, 1.05) | 1.01 (0.87, 1.17) |
| Public administration | | 1.01 (0.85, 1.20) | | 0.98 (0.89, 1.09) | 0.97 (0.87, 1.09) | 0.96 (0.86, 1.08) | 0.99 (0.87, 1.13) | 0.96 (0.84, 1.11) | 1.01 (0.88, 1.16) | 1.01 (0.88, 1.15) | 1.00 (0.90, 1.11) | 0.97 (0.87, 1.09) | 0.99 (0.88, 1.12) |
| Unemployed | | 0.97 (0.88, 1.07) | | 1.09 (1.03, 1.16) | 1.01 (0.95, 1.08) | 1.00 (0.93, 1.06) | 0.99 (0.91, 1.07) | 0.97 (0.89, 1.05) | 0.97 (0.89, 1.05) | 1.01 (0.94, 1.10) | 1.00 (0.94, 1.07) | 1.00 (0.94, 1.07) | 1.00 (0.93, 1.07) |
|  |  | | **MALE (n = 2,135)** | | | | | | | | | | |
| Agriculture, forestry, fishing and hunting | | 0.86 (0.59, 1.25) | | 1.04 (0.85, 1.28) | 1.06 (0.83, 1.35) | 1.04 (0.81, 1.32) | 0.94 (0.70, 1.25) | 1.26 (0.96, 1.66) | 1.52 (1.14, 2.03) | 0.95 (0.71, 1.28) | 1.19 (0.95, 1.49) | 1.05 (0.82, 1.33) | 1.01 (0.77, 1.32) |
| Mining, quarrying, and oil and gas extraction | | 0.83 (0.55, 1.26) | | 0.96 (0.76, 1.21) | 1.06 (0.81, 1.39) | 1.07 (0.82, 1.40) | 1.05 (0.76, 1.43) | 1.00 (0.74, 1.35) | 1.11 (0.81, 1.53) | 0.89 (0.64, 1.23) | 1.12 (0.87, 1.44) | 1.06 (0.81, 1.39) | 1.05 (0.78, 1.40) |
| Utilities | | 1.34 (0.76, 2.36) | | 1.33 (0.96, 1.84) | 1.08 (0.75, 1.55) | 1.03 (0.72, 1.49) | 0.99 (0.63, 1.54) | 0.86 (0.57, 1.31) | 1.24 (0.80, 1.92) | 1.31 (0.83, 2.05) | 1.15 (0.81, 1.63) | 1.09 (0.75, 1.58) | 1.36 (0.91, 2.03) |
| Construction | | 0.99 (0.82, 1.19) | | 1.03 (0.93, 1.14) | 1.10 (0.98, 1.24) | 1.09 (0.97, 1.23) | 1.10 (0.95, 1.27) | 1.14 (0.99, 1.30) | 1.13 (0.98, 1.31) | 1.00 (0.87, 1.16) | 1.08 (0.97, 1.21) | 1.08 (0.96, 1.22) | 1.03 (0.90, 1.17) |
| Manufacturing | | 0.98 (0.82, 1.15) | | 0.96 (0.87, 1.05) | 0.94 (0.84, 1.05) | 0.96 (0.86, 1.07) | 1.03 (0.90, 1.17) | 0.96 (0.85, 1.09) | 0.93 (0.81, 1.05) | 1.00 (0.88, 1.14) | 0.94 (0.85, 1.04) | 0.96 (0.86, 1.07) | 0.98 (0.87, 1.10) |
| Wholesale trade | | 1.08 (0.82, 1.41) | | 0.90 (0.77, 1.05) | 1.10 (0.93, 1.31) | 1.11 (0.93, 1.33) | 1.08 (0.88, 1.34) | 0.88 (0.72, 1.08) | 1.14 (0.93, 1.41) | 1.02 (0.82, 1.26) | 1.10 (0.93, 1.30) | 1.11 (0.93, 1.33) | 1.10 (0.90, 1.34) |
| Retail trade | | 1.05 (0.83, 1.32) | | 0.95 (0.84, 1.09) | 0.99 (0.85, 1.15) | 1.01 (0.87, 1.17) | 0.94 (0.79, 1.13) | 0.99 (0.83, 1.18) | 1.08 (0.90, 1.29) | 1.00 (0.84, 1.21) | 1.01 (0.88, 1.17) | 0.99 (0.86, 1.16) | 1.03 (0.87, 1.22) |
| Transportation and warehousing | | 1.35 (1.06, 1.72) | | 1.04 (0.91, 1.19) | 0.94 (0.80, 1.10) | 0.93 (0.80, 1.09) | 0.85 (0.70, 1.02) | 1.01 (0.85, 1.21) | 1.00 (0.83, 1.20) | 1.22 (1.00, 1.47) | 0.92 (0.79, 1.07) | 0.93 (0.80, 1.09) | 1.11 (0.93, 1.32) |
| Information and cultural industries | | 1.18 (0.83, 1.68) | | 0.95 (0.78, 1.15) | 1.35 (1.08, 1.69) | 1.34 (1.06, 1.68) | 1.27 (0.97, 1.67) | 1.08 (0.83, 1.40) | 0.83 (0.63, 1.08) | 1.05 (0.79, 1.39) | 1.14 (0.92, 1.42) | 1.32 (1.05, 1.66) | 1.05 (0.81, 1.36) |
| Finance and insurance | | 0.91 (0.69, 1.20) | | 1.08 (0.92, 1.26) | 1.01 (0.84, 1.20) | 0.97 (0.81, 1.17) | 1.10 (0.88, 1.36) | 1.00 (0.82, 1.23) | 0.82 (0.66, 1.02) | 1.02 (0.82, 1.26) | 0.96 (0.81, 1.14) | 1.00 (0.83, 1.19) | 1.00 (0.82, 1.22) |
| Real estate and rental and leasing | | 0.92 (0.58, 1.45) | | 0.98 (0.76, 1.27) | 1.11 (0.83, 1.49) | 1.12 (0.84, 1.51) | 1.17 (0.83, 1.66) | 1.41 (1.01, 1.96) | 0.98 (0.69, 1.40) | 0.98 (0.69, 1.41) | 1.11 (0.85, 1.46) | 1.12 (0.84, 1.50) | 1.05 (0.76, 1.44) |
| Professional, scientific and technical services | | 0.85 (0.70, 1.04) | | 0.91 (0.82, 1.02) | 0.94 (0.83, 1.07) | 0.97 (0.85, 1.11) | 1.02 (0.87, 1.18) | 0.93 (0.80, 1.07) | 0.87 (0.75, 1.02) | 0.88 (0.75, 1.03) | 0.98 (0.87, 1.10) | 0.97 (0.86, 1.10) | 0.89 (0.78, 1.03) |
| Administrative and support, waste management and remediation services | | 1.25 (0.91, 1.71) | | 0.95 (0.80, 1.14) | 0.91 (0.74, 1.11) | 0.89 (0.72, 1.09) | 0.82 (0.65, 1.05) | 1.09 (0.87, 1.38) | 0.90 (0.70, 1.14) | 1.10 (0.86, 1.41) | 0.90 (0.74, 1.09) | 0.89 (0.72, 1.09) | 1.03 (0.83, 1.29) |
| Educational services | | 0.85 (0.64, 1.11) | | 0.95 (0.81, 1.10) | 0.96 (0.80, 1.14) | 0.96 (0.80, 1.14) | 1.00 (0.81, 1.23) | 0.94 (0.77, 1.15) | 1.07 (0.87, 1.32) | 0.90 (0.73, 1.11) | 0.98 (0.83, 1.15) | 0.97 (0.81, 1.15) | 0.93 (0.77, 1.12) |
| Health care and social assistance | | 1.14 (0.87, 1.50) | | 1.06 (0.91, 1.24) | 1.08 (0.90, 1.28) | 1.10 (0.92, 1.31) | 1.07 (0.87, 1.32) | 0.98 (0.80, 1.19) | 1.12 (0.91, 1.39) | 1.12 (0.91, 1.39) | 1.03 (0.88, 1.21) | 1.07 (0.90, 1.28) | 1.12 (0.92, 1.35) |
| Arts, entertainment and recreation | | 1.12 (0.77, 1.61) | | 0.96 (0.78, 1.17) | 0.94 (0.74, 1.19) | 0.98 (0.77, 1.25) | 0.84 (0.63, 1.13) | 1.02 (0.78, 1.34) | 1.00 (0.75, 1.33) | 1.02 (0.77, 1.36) | 0.99 (0.79, 1.24) | 0.97 (0.76, 1.23) | 1.02 (0.79, 1.32) |
| Accommodation and food services | | 0.94 (0.71, 1.24) | | 0.98 (0.84, 1.14) | 1.02 (0.85, 1.22) | 1.05 (0.87, 1.25) | 1.10 (0.89, 1.36) | 1.11 (0.90, 1.36) | 0.86 (0.70, 1.07) | 0.92 (0.74, 1.14) | 0.97 (0.82, 1.15) | 1.04 (0.87, 1.24) | 0.91 (0.75, 1.11) |
| Other services (except public administration) | | 0.85 (0.61, 1.19) | | 1.09 (0.90, 1.32) | 0.89 (0.72, 1.10) | 0.88 (0.71, 1.10) | 0.89 (0.68, 1.16) | 1.02 (0.79, 1.30) | 1.07 (0.83, 1.39) | 0.95 (0.73, 1.24) | 0.93 (0.75, 1.14) | 0.87 (0.70, 1.09) | 0.94 (0.74, 1.20) |
| Public administration | | 0.94 (0.73, 1.20) | | 1.00 (0.87, 1.15) | 0.83 (0.70, 0.97) | 0.83 (0.71, 0.98) | 0.83 (0.69, 1.01) | 0.98 (0.81, 1.18) | 1.07 (0.88, 1.30) | 0.98 (0.80, 1.19) | 0.93 (0.80, 1.09) | 0.83 (0.71, 0.98) | 0.94 (0.79, 1.12) |
| Unemployed | | 1.01 (0.86, 1.19) | | 1.09 (1.00, 1.20) | 1.04 (0.94, 1.16) | 1.01 (0.90, 1.12) | 0.99 (0.88, 1.13) | 0.92 (0.81, 1.03) | 0.95 (0.84, 1.08) | 1.00 (0.88, 1.14) | 1.00 (0.90, 1.10) | 1.01 (0.91, 1.13) | 0.98 (0.88, 1.10) |
|  |  | | **FEMALE (n = 2,124)** | | | | | | | | | | |
| Agriculture, forestry, fishing and hunting | | 0.70 (0.40, 1.24) | | 0.90 (0.64, 1.26) | 1.34 (0.92, 1.95) | 1.33 (0.91, 1.95) | 1.20 (0.77, 1.86) | 0.97 (0.61, 1.54) | 1.23 (0.76, 1.96) | 0.73 (0.47, 1.12) | 1.26 (0.89, 1.79) | 1.31 (0.90, 1.91) | 0.85 (0.58, 1.26) |
| Mining, quarrying, and oil and gas extraction | | 2.08 (0.80, 5.39) | | 0.79 (0.45, 1.39) | 1.08 (0.57, 2.04) | 1.03 (0.54, 1.95) | 1.07 (0.51, 2.24) | 0.85 (0.39, 1.85) | 1.59 (0.72, 3.52) | 1.22 (0.55, 2.71) | 1.12 (0.62, 2.03) | 1.05 (0.56, 1.98) | 1.18 (0.58, 2.40) |
| Utilities | | 0.92 (0.38, 2.22) | | 1.16 (0.69, 1.96) | 1.24 (0.69, 2.24) | 1.28 (0.71, 2.31) | 1.54 (0.77, 3.05) | 0.98 (0.45, 2.13) | 2.08 (1.00, 4.34) | 0.97 (0.49, 1.92) | 1.39 (0.77, 2.52) | 1.27 (0.71, 2.28) | 1.00 (0.52, 1.91) |
| Construction | | 1.16 (0.74, 1.81) | | 1.00 (0.77, 1.29) | 0.87 (0.65, 1.17) | 0.87 (0.65, 1.17) | 0.72 (0.51, 1.01) | 0.95 (0.66, 1.36) | 1.01 (0.70, 1.47) | 1.11 (0.79, 1.55) | 0.90 (0.69, 1.19) | 0.85 (0.64, 1.14) | 1.10 (0.82, 1.49) |
| Manufacturing | | 1.21 (0.91, 1.62) | | 0.83 (0.70, 0.98) | 0.81 (0.67, 0.99) | 0.79 (0.65, 0.96) | 0.81 (0.64, 1.02) | 0.95 (0.75, 1.21) | 0.86 (0.67, 1.09) | 1.02 (0.82, 1.28) | 0.81 (0.67, 0.98) | 0.80 (0.65, 0.97) | 0.94 (0.76, 1.15) |
| Wholesale trade | | 0.88 (0.57, 1.34) | | 0.98 (0.77, 1.26) | 1.06 (0.80, 1.40) | 1.09 (0.82, 1.45) | 1.14 (0.82, 1.58) | 0.88 (0.62, 1.24) | 0.84 (0.59, 1.21) | 0.93 (0.67, 1.29) | 1.03 (0.79, 1.34) | 1.08 (0.82, 1.43) | 0.92 (0.68, 1.23) |
| Retail trade | | 0.94 (0.77, 1.14) | | 0.97 (0.87, 1.10) | 0.97 (0.85, 1.10) | 0.98 (0.86, 1.12) | 0.92 (0.78, 1.07) | 0.96 (0.81, 1.12) | 0.98 (0.83, 1.16) | 0.94 (0.80, 1.09) | 0.97 (0.85, 1.10) | 0.97 (0.85, 1.10) | 0.93 (0.81, 1.07) |
| Transportation and warehousing | | 1.08 (0.78, 1.49) | | 0.91 (0.75, 1.10) | 1.00 (0.81, 1.25) | 1.03 (0.83, 1.28) | 1.01 (0.78, 1.30) | 0.93 (0.71, 1.22) | 0.78 (0.59, 1.02) | 1.00 (0.78, 1.29) | 0.90 (0.73, 1.11) | 1.01 (0.82, 1.26) | 0.96 (0.76, 1.21) |
| Information and cultural industries | | 1.20 (0.77, 1.88) | | 0.94 (0.73, 1.23) | 0.89 (0.66, 1.20) | 0.91 (0.68, 1.23) | 0.96 (0.68, 1.36) | 1.11 (0.77, 1.61) | 0.91 (0.63, 1.32) | 1.13 (0.80, 1.61) | 0.91 (0.69, 1.21) | 0.90 (0.67, 1.21) | 1.06 (0.78, 1.45) |
| Finance and insurance | | 1.14 (0.86, 1.52) | | 0.99 (0.84, 1.17) | 1.07 (0.88, 1.29) | 1.07 (0.88, 1.29) | 1.13 (0.91, 1.41) | 0.98 (0.78, 1.24) | 0.80 (0.63, 1.01) | 1.10 (0.88, 1.38) | 1.01 (0.85, 1.21) | 1.07 (0.89, 1.29) | 1.09 (0.90, 1.34) |
| Real estate and rental and leasing | | 0.94 (0.56, 1.56) | | 0.89 (0.66, 1.21) | 1.13 (0.80, 1.60) | 1.07 (0.76, 1.50) | 0.93 (0.62, 1.38) | 0.97 (0.64, 1.46) | 0.80 (0.52, 1.22) | 0.88 (0.59, 1.30) | 0.99 (0.72, 1.37) | 1.13 (0.80, 1.60) | 0.98 (0.69, 1.40) |
| Professional, scientific and technical services | | 0.94 (0.76, 1.17) | | 0.90 (0.79, 1.02) | 0.92 (0.80, 1.07) | 0.95 (0.82, 1.10) | 0.94 (0.80, 1.12) | 0.89 (0.74, 1.06) | 0.81 (0.67, 0.97) | 0.92 (0.78, 1.09) | 0.91 (0.79, 1.04) | 0.94 (0.81, 1.08) | 0.96 (0.82, 1.12) |
| Administrative and support, waste management and remediation services | | 1.15 (0.88, 1.50) | | 1.05 (0.90, 1.23) | 0.97 (0.81, 1.16) | 0.98 (0.82, 1.17) | 0.95 (0.77, 1.18) | 1.01 (0.81, 1.26) | 0.96 (0.77, 1.20) | 1.11 (0.90, 1.36) | 0.96 (0.81, 1.14) | 0.98 (0.82, 1.17) | 1.10 (0.91, 1.33) |
| Educational services | | 0.86 (0.71, 1.04) | | 1.23 (1.10, 1.38) | 1.10 (0.97, 1.25) | 1.09 (0.96, 1.24) | 1.12 (0.96, 1.30) | 1.09 (0.93, 1.28) | 1.19 (1.02, 1.40) | 0.99 (0.85, 1.14) | 1.12 (0.99, 1.26) | 1.11 (0.98, 1.26) | 1.01 (0.88, 1.16) |
| Health care and social assistance | | 1.01 (0.88, 1.17) | | 0.91 (0.84, 0.99) | 0.93 (0.85, 1.03) | 0.93 (0.84, 1.03) | 0.96 (0.85, 1.07) | 1.00 (0.89, 1.12) | 1.16 (1.03, 1.31) | 0.96 (0.86, 1.07) | 0.98 (0.90, 1.08) | 0.93 (0.84, 1.02) | 0.95 (0.85, 1.05) |
| Arts, entertainment and recreation | | 0.94 (0.64, 1.39) | | 1.13 (0.90, 1.41) | 1.20 (0.93, 1.56) | 1.14 (0.88, 1.48) | 1.32 (0.97, 1.79) | 1.30 (0.95, 1.78) | 1.18 (0.86, 1.63) | 1.03 (0.77, 1.39) | 1.22 (0.96, 1.55) | 1.17 (0.91, 1.52) | 1.10 (0.84, 1.43) |
| Accommodation and food services | | 0.99 (0.78, 1.27) | | 0.93 (0.81, 1.08) | 1.16 (0.99, 1.36) | 1.16 (0.98, 1.36) | 1.02 (0.84, 1.24) | 0.99 (0.81, 1.20) | 0.99 (0.81, 1.21) | 1.00 (0.83, 1.21) | 1.09 (0.93, 1.27) | 1.15 (0.97, 1.35) | 1.06 (0.90, 1.26) |
| Other services (except public administration) | | 1.16 (0.89, 1.51) | | 1.10 (0.94, 1.28) | 0.93 (0.78, 1.11) | 0.95 (0.79, 1.13) | 1.01 (0.82, 1.25) | 1.22 (0.98, 1.51) | 1.13 (0.90, 1.40) | 1.09 (0.88, 1.33) | 1.01 (0.85, 1.20) | 0.94 (0.79, 1.13) | 1.05 (0.87, 1.27) |
| Public administration | | 1.10 (0.86, 1.40) | | 0.96 (0.83, 1.10) | 1.15 (0.98, 1.35) | 1.12 (0.95, 1.31) | 1.17 (0.97, 1.41) | 0.95 (0.77, 1.16) | 0.96 (0.79, 1.18) | 1.04 (0.86, 1.26) | 1.07 (0.92, 1.25) | 1.13 (0.96, 1.32) | 1.05 (0.89, 1.25) |
| Unemployed | | 0.94 (0.83, 1.07) | | 1.09 (1.01, 1.18) | 0.99 (0.91, 1.08) | 0.98 (0.90, 1.07) | 0.98 (0.89, 1.09) | 1.01 (0.91, 1.12) | 0.98 (0.88, 1.09) | 1.03 (0.93, 1.13) | 1.00 (0.92, 1.09) | 0.99 (0.91, 1.08) | 1.01 (0.92, 1.11) |

Abbreviations: CHMS, Canadian Health Measures Survey; GMR, Geometric mean ratio; CI, Confidence interval; DF, Detection frequency; MEP, Mono-ethyl phthalate; MnBP, Mono-n-butyl phthalate; MEOHP, Mono-(2-ethyl-5-oxohexyl) phthalate; MEHHP, Mono-(2-ethyl-5-hydroxyhexyl) phthalate; MEHP, Mono-(2-ethylhexyl) phthalate; MCPP, Mono-(3-carboxypropyl) phthalate; MBzP, Mono-benzyl phthalate; MMP, Mono-methyl phthalate; MCHP, Mono-cyclohexyl phthalate; MiNP, Mono-isononyl phthalate; MOP, Mono-octyl phthalate; LMWP, Low molecular weight phthalate; HMWP, High molecular weight phthalate; DEHP, Di(2-ethylhexyl) phthalate.

^a^Based on geometric mean concentration units of μg/g creatinine.

^b^Based on geometric mean concentration units of nmol/g creatinine.

∑LMWP = Molar sum of MMP + MEP + MnBP + MCHP.

∑HMWP = Molar sum of MEOHP + MEHHP + MEHP + MiNP + MOP + MCPP + MBzP.

∑DEHP = Molar sum of MEOHP + MEHHP + MEHP.

∑Total = Molar Sum of MMP + MEP + MnBP + MCHP + MEOHP + MEHHP + MEHP + MiNP + MOP + MCPP + MBzP.

^c^Least-squares GMRs computed by multivariable linear regression, using sector-level industry of employment terms as binary indicators, adjusting for: age (continuous), sex (in ‘overall’ but not sex-stratified models), CHMS cycle, ethnicity, household income, smoking status, six dietary consumptions groups (meat and alternatives, dairy, grains, fruits and vegetables, seafood, bottled/canned beverages), drinking water type, and physical activity.

**References**

1. Liu H, Wang Y, Kannan K, Liu M, Zhu H, Chen Y, et al. Determinants of Phthalate Ex posures in Pregnant Women in New York City. Environ Res. 2022 Sep;212(Pt A):113203. doi:10.1016/j.envres.2022.113203 PubMed PMID: 35358547; PubMed Central PMCID: PMC9232940.

2. Pullella K. Elucidating the Relationship between Arsenic Exposure and Cancer Risk in Canada [Internet]. 2024 [cited 2026 Mar 20]. Available from: http://hdl.handle.net/1807/140492

3. Hosseini Z, Whiting SJ, Vatanparast H. Canadians’ Dietary Intake from 2007 to 2011 and across Different Sociodemographic/Lifestyle Factors Using the Canadian Health Measures Survey Cycles 1 and 2. J Nutr Metab. 2019;2019:2831969. doi:10.1155/2019/2831969 PubMed PMID: 30867963; PubMed Central PMCID: PMC6379838.
